# Dual-Capped Helical Interface Mimics

**DOI:** 10.1021/jacs.3c11717

**Published:** 2024-04-04

**Authors:** Tianxiong Mi, Zhe Gao, Zeynep Mituta, Kevin Burgess

**Affiliations:** † Department of Chemistry, Texas A & M University, Box 30012, College Station, Texas 77842, United States; ‡ ZentriForce Pharma Research GmbH, Carl-Friedrich-Gauss-Ring 5, 69124 Heidelberg, Germany

## Abstract

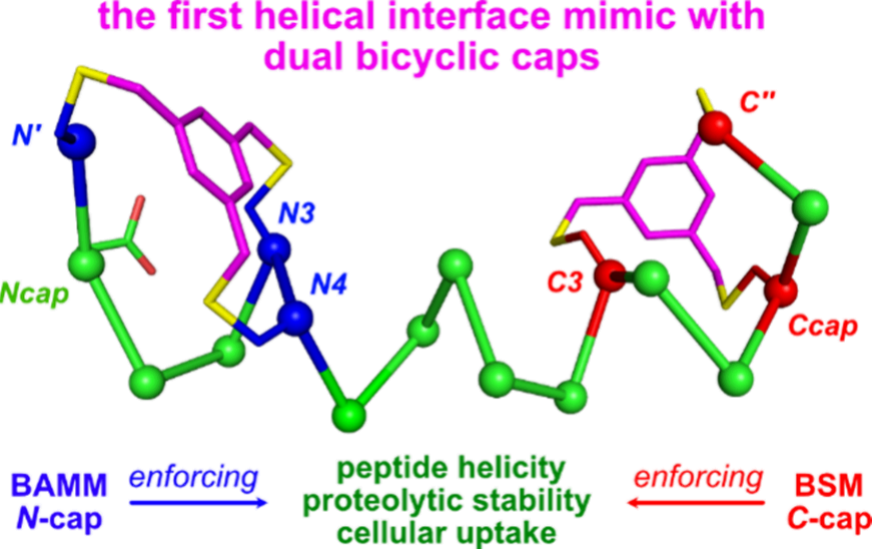

Disruption of protein–protein interactions is medicinally important. Interface helices may be mimicked in helical probes featuring enhanced rigidities, binding to protein targets, stabilities in serum, and cell uptake. This form of mimicry is dominated by stapling between side chains of helical residues: there has been less progress on helical *N*-caps, and there were no generalizable *C*-caps. Conversely, in natural proteins, helicities are stabilized and terminated by *C*- and *N-*caps but not staples. Bicyclic caps previously introduced by us enable interface helical mimicry featuring rigid synthetic caps at both termini in this work. An unambiguously helical dual-capped system proved to be conformationally stable, binding cyclins A and E, and showed impressive cellular uptake. In addition, the dual-capped mimic was completely resistant to proteolysis in serum over an extended period when compared with “gold standard” hydrocarbon-stapled controls. Dual-capped peptidomimetics are a new, generalizable paradigm for helical interface probe design.

## Introduction

Disruption of protein–protein interactions (PPIs)^1^ using synthetic molecules resembling interface segments is valuable for generation of probes and pharmaceutical leads.^2−8^ “Helical interface mimicry” features helical interface regions.^9−11^ It is useful to regard proteins with the interface helices as *protein ligands*, binding to their *protein receptors*. Stabilities of helical conformations in the absence of protein and affinities for the protein receptor are indicators of the effectiveness of helical mimics. The next challenge in probe development is serum stability; robust mimics are expected to have higher effective concentrations, reaching the target *in vivo*. Consequently, protein receptor binding and serum stabilities are two early stage benchmarks to compare probes.

**Figure 1:**
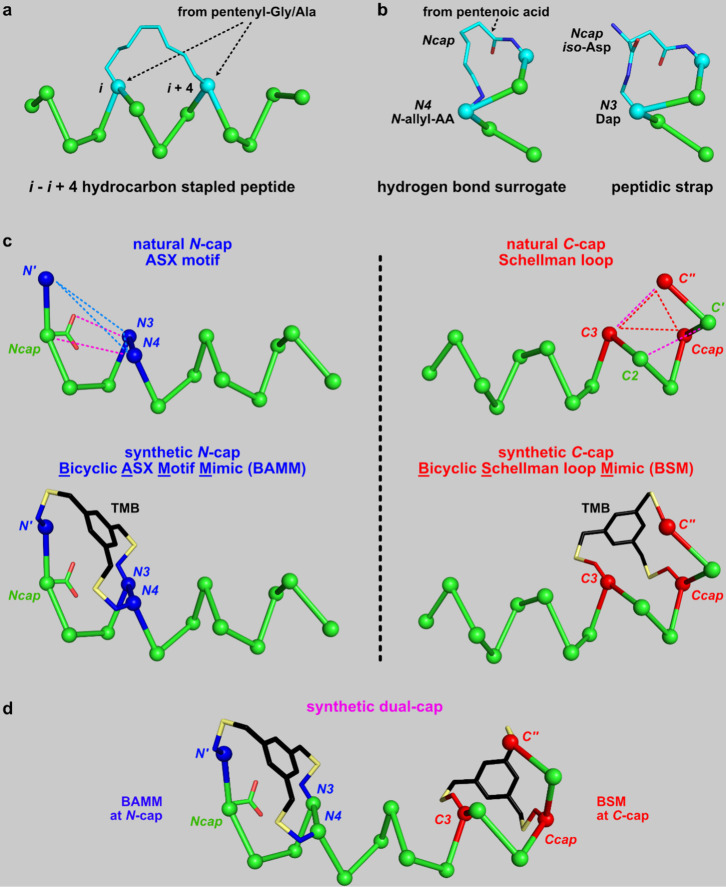
Typical: (a) hydrocarbon staple (PDB 4MZK); (b) synthetic *N*-caps (PDB 4MZL and 5GS4); (c) natural caps with hydrophobic triangles (upper row) and corresponding synthetic bicyclic caps (bottom row);^46,50^ and (d) a dual cap comprising a BAMM and BSM.

Staples are characterized by joining side chains of at least two amino acids in the helical region, *i.e.*, having α-helical φ,ψ dihedral angles of ∼−60,–40°. Stapling of helices (*e.g.*, Figure 1a) prevails in helical mimicry. The field began with staples comprising amides linked by natural amino acids with carboxylic acid and amine side chains,^12^ including single sequences with two overlapping^13^ or three distinct staples.^14,15^ Now it has expanded to include many different compositions.^16−19^ A milestone was formation of hydrocarbon staples^20−26^ via alkene metathesis,^27−31^ typically (Figure 1a) via pentenyl-Gly and -Ala derivatives. This is probably the most widely applied stapling method; it is arguably the current gold standard for helical mimicry.

Synthetic *capping* motifs are distinct from staples.^32^ They comprise modifications to amino acids immediately outside the helical motif, *i.e.*, ones for which φ,ψ deviates significantly from −60,–40°. Capping methodologies have been employed significantly less often than stapling. Development of synthetic caps has been almost^33,34^ exclusively for *N*-termini.^35−37^ Contemporary methods for *N*-capping involve amino acid side chains joined via amide bonds (*e.g.*, Figure 1b right: an *iso*-Asp amide linked to diaminopropionic acid, Dap),^38−40^ or *N*-allyl amino acid substrates for alkene metathesis to give alkenes in place of hydrogen bonds, *i*.*e*., *H*-bond surrogates (Figure 1b left: *N*-allyl amino acid and *N*-terminal 4-pentenoic acid joined).^41−43^

Progress on capped helical interface mimics is retarded by (i) lack of generalizable and helix-inducing peptidomimetics capped at the *C*-terminus; (ii) capped mimics comprising only one monocyclic ring resulting in suboptimal helical rigidities; and (iii) inconvenient requirements for modified amino acid building blocks (*e.g.*, *N*-allyl protected amino acids {*H*-bond surrogates}). Consequently, the literature to date emphasizes new stapling methods and applications of hydrocarbon staples in helical mimicry. There is less research on synthetic *N*-caps and hardly anything on *C*-caps. Studies featuring side-by-side comparisons of helical interface mimics^44,45^ are rare.

We entered this area by using bioinformatics to design synthetic *bicyclic* caps. Bicyclic Schellman loop Mimics (BSMs) enforce helicities and reorient the *C*-terminus,^46^ and Bicyclic ASX Motif Mimics (BAMMs) in the press function similarly at the *N*-terminus.^53^ Consequently, we are potentially able to “dual cap” peptides at both termini. We hypothesized mimics with *two bicyclic* caps would be exceptionally biased to helical conformations; hence, they could be superior binders to protein receptor targets. We also anticipated capping at both ends would impart stability in serum, and, possibly, cell uptake. This would be significant because optimization of helicity, protein binding, stability in serum, and cell uptake are the chemical challenges in this area, after which the focus turns to pharmacokinetics. The specific aims of this research were to explore those issues by comparing a dual-capped peptide (“dual”) with the corresponding wild type (“linear”), *C*- and *N*-capped (“BSM” and “BAMM”), and two hydrocarbon staple controls (“staples 1 and 2”). We strove to demonstrate generalizable bicyclic dual capping methodology for the first time and compare it to these gold standard helical staple systems.

Tens of thousands of PPIs contain helical interface segments.^10,11,47^ No one helical mimic design would work uniformly well in each case, and it is logistically impossible to research a statistically significant sample, so we chose one interesting PPI. The wild-type *C*-helix sequence of CDK2 binding to cyclin E was selected because: (i) helical mimicry for CDK2·cyclin E is new; (ii) it is illustrative of PPIs which lead to phosphorylation of retinoblastoma protein (Rb, phosphorylation of which to pRb “lifts the suppression handbrake” and leads to uncontrolled cell cycling and growth in many tumor types^48,49^); and (iii) the 13-residue CDK2 *C*-helix system is not long enough to fold into a helix in aqueous solution but has sufficient residues to accommodate two capping motifs. Staples 1 and 2 are typical *i*–*i*+4 systems. We modeled the positioning of their hydrocarbon linkers so they would not impede binding to cyclin E.

## Results and Discussion

### Molecular Dynamics (MD) to Explore Dual Capping Feasibility

**Figure 2:**
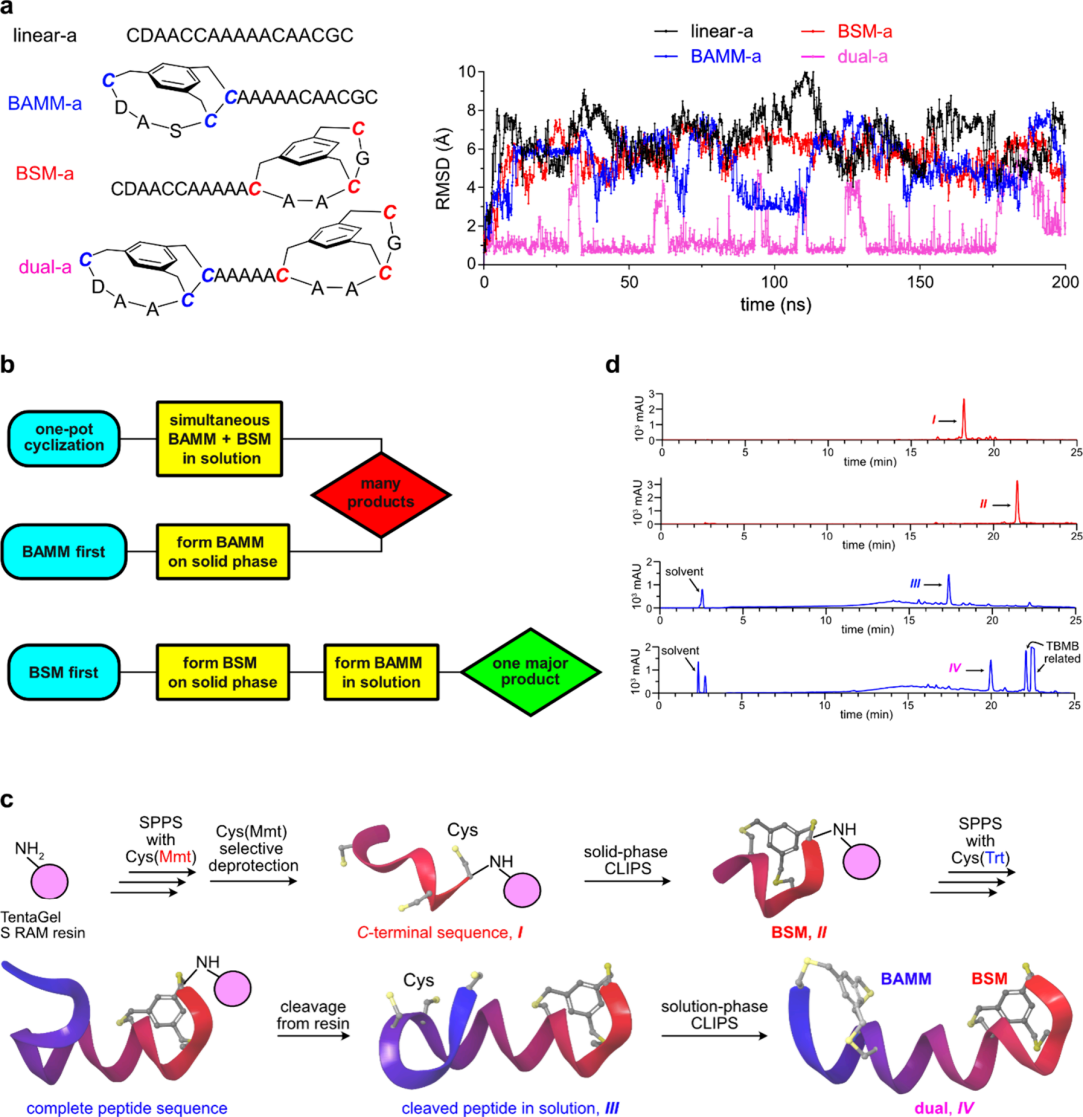
(a) Compounds were simulated using MD over an extended time (200 ns). (b) Attempts to synthesize dual culminating in strategy 3 “BSM first” (c). (d) Crude purity of dual by anal-HPLC: construction of the BSM first (red trace, I–II in part c) and then the BAMM fragments (blue, III, and purple, IV).

Figure 2a shows Ala-rich sequences (“linear-a”, “BAMM-a”, “BSM-a”, and “dual-a”, all 17-mers; left), assembled into ideal α-helical conformations and then subjected to MD (explicit water, 300 K, using Desmond^51^ over 200 ns; right). Resistance to unfolding in these experiments is an indicator of helical stability. Root mean square deviations (RMSD) from starting helical conformations during MD as a function of time are representative of helical robustness; low RMSDs indicate stabilized helical conformations. Observations were: (i) rapid, irreversible, loss of helicity from *N*- and *C*-termini for linear-a; (ii) loss of helicity at uncapped termini for BAMM-a and BSM-a; and (iii) persistent helicity for dual-a. Dual-a only occasionally, and reversibly, lost helicity until ∼175 ns, when all the systems were already relatively disordered (Figure 2a right; movie clips of the MD run in SI materials). Another three peptides with previously known *N*-caps or a hydrocarbon staple were also studied by MD simulations (Figure S10); they were also affected by loss of helicity from the unconstrained termini and hence were less helix-stabilizing than dual-a.

### Synthesis of a Dual-Capped System

An efficient synthesis route for dual-capped peptidomimetics was critical. Initially, the most directly conceivable approach (*one-pot cyclization*; Figure 2b top) was attempted. A peptide precursor was made on a solid phase, deprotected, and cleaved into solution to give a hexa-Cys system then reacted with 1,3,5-tri(bromomethylene)benzene (TBMB); many products were formed (Figure S1). Next explored was strategy 2 featuring early BAMM fragment construction on a solid phase (Figure 2b *BAMM first*; middle), but this also gave many products (Figure S2b). Fortunately, strategy 3, *BSM first* worked well (Figures 2b bottom, 2c, and S2a). Thus, the *C*-terminal sequence of the peptidomimetic was first assembled on the solid phase to give the BSM fragment after selective removal of Cys(*S*-Mmt) (monomethoxy trityl) protection then reaction with TBMB by solid phase CLIPS (Chemical Linkage of Peptides onto Scaffolds) reaction; formation of one predominant product was verified by HPLC. Solid phase synthesis was then continued, three Cys(*S*-Trt) were incorporated, and the whole system was simultaneously deprotected, cleaved from the resin, and then finally reacted again with TBMB but in solution to form the BAMM fragment. This successful strategy separates the formation of two bicyclic caps into noncompeting stages and gives crude material comprising only one major peptide product (Figure 2d), which can be conveniently purified via prep HPLC.

### Helicities

**Figure 3:**
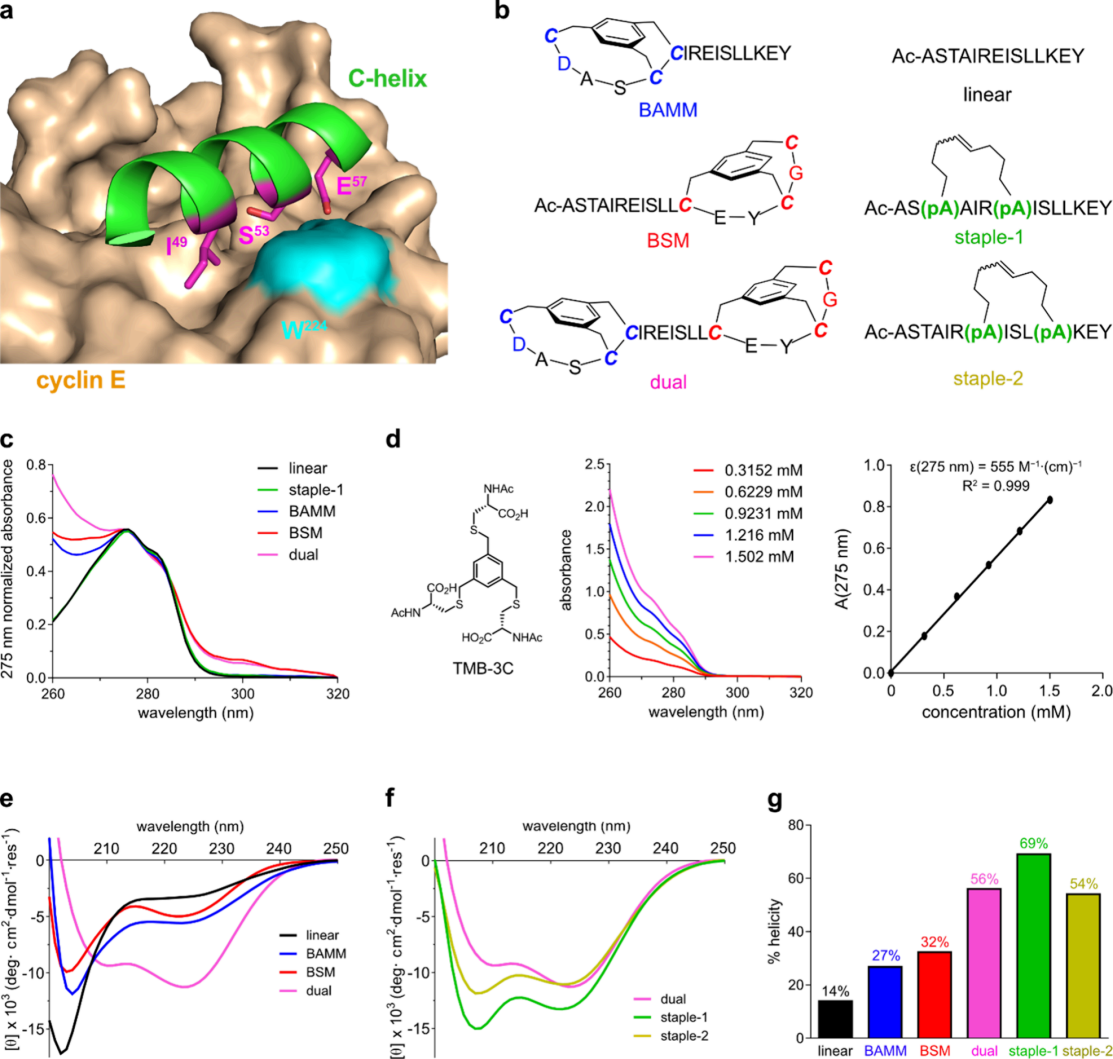
(a) *C*-helix binding cyclin E (1W98). Cyclin E hot spots are in magenta, and tryptophan (W, cyan) is used for FQ. (b) Sequences of the test compounds. (c) UV absorbance spectra (260–320 nm) of tested peptides normalized by absorbance intensities at 275 nm. (d) Calibration of the UV absorbance coefficient at 275 nm of trithiol-alkylated 1,3,5-trimethylbenzene (TMB) by Beer–Lambert Law. (e) CD spectra for linear, BAMM, BSM, and dual in PBS pH 7.4, 25 °C. (f) Dual as well as staples 1 and 2 under the same conditions. (g) Experimental percent helicity of the tested peptides.

Figure 3a,b shows CDK2·cyclin E (PDB entry 1W98) and how sequences of linear, BSM, BAMM, dual, and staples 1/2 relate to the CDK2 *C*-helix (SI section F). Pink residues in Figure 3a (I,^49^ S,^53^ E^57^) are hot spots from CDK2 *C*-helix binding cyclin E as determined by solvent accessible surface area (SASA)^52,53^ calculations. All six systems in Figure 3b encompass these hot spots.

BAMMs and BSMs are not simply *pseudo*-mirror images; they carefully parallel ASX motifs and Schellman loops in natural helical caps of proteins.^46,50^ BAMM units, ***C***DXX***CC*** (-*N′*-*Ncap*-*N1*-*N2*-*N3*-*N4*-, where the helix starts at *N1*, X is any amino acid), incorporate two nonhelical residues (***C***D···, -*N′*-*Ncap*-; throughout “***C***” represents Cys capped with TMB), while BSM units, ***C***XX***C***G***C*** (-*C3*-*C2*-*C1*-*Ccap*-*C′*-*C″*-, where the helix stops at *C1*) incorporate three (···***C***G***C***, -*Ccap*-*C′*-*C″*-). We expect all residues in the linear sequence to be helical, so BAMM is longer than linear by *two* residues (the *N*-terminal ***C*** and D), BSM is longer by *three* (the *C*-terminal ***C***G***C*** sequence), and dual is longer by a total of *five* (***C***D ··· ***C***G***C***). Stapled peptides-1/2 have the same number of amino acids as the linear control.

Accurate concentration measurements are important when comparing the peptide and peptidomimetic helicities. Thus, a Tyr (Y; not present in the CDK2 *C*-helix) was included so concentrations of tested peptides could be calculated by UV absorbance at 275 nm. However, UV spectra of prepared peptides showed linear and stapled peptides had similar shapes but bicyclic capped peptides were different: their UV intensities increased dramatically before 270 nm (Figure 3c). This suggested the additional trithiol-alkylated TMB group could significantly contribute to the absorbance around that area, so a calibration for this module was required. Consequently, TMB-3C was prepared for this purpose, and its UV spectra, as well as extinction coefficient at 275 nm, were quantified by the Beer–Lambert law (Figure 3d). This gives a value of 555 (M^–1^·cm^–1^), more than one-third of the value for Tyr: 1455 (M^–1^·cm^–1^); hence, it is not negligible.

Concentration dependent circular dichroism (CD) studies were conducted for dual and stapled peptides in PBS. Their mean-residue ellipticities at 222 nm gradually increased since 5 μM and finally reached stable states at higher concentrations: dual, 13 μM; staple-1, 17 μM; staple-2, 22 μM (Figure S4). These concentrations were used to obtain their CD spectra as shown in Figure 3e,f. The observation suggested a helix-initiation process for these peptides, probably by aggregation or formation of oligomeric coiled coils. Similar phenomena were not observed for linear and monocapped peptides which were less helical.

All six systems were also studied in different ratios of trifluoroethanol:PBS (up to 60% for linear and 50% for the others, Figure S6) to reach experimentally maximal helical states (as other have^54,55^) and obtain their maximal 222 nm ellipticities [θ]_max_; thus, the experimental percent helicity can be calculated by [θ]_222 in PBS_/[θ]_max_ (Figure 3g and SI section C).

In PBS at 25 °C, BSM (red) and BAMM (blue) were similarly helical and more so than linear, which has little helicity (222:208 ratios, and 222 nm ellipticities are relevant;^41^Figure 3e and Table 1). This suggested one cap was enough to measurably induce helicity. Dual, however, had more than 20% improvement of percent helicities over BAMM and BSM. Compared with two staple controls, dual had higher percent helicity than staple-2 but lower than staple-1 (Figure 3f,g). CD shape is also relevant; the 222/208 ratios indicate dual could be helical, but this is not definitive due to the potential influence of TMB on CD shapes.

**Table 1:** Helical Propensities, Binding Affinities, and Serum Stabilities for the Featured Peptides

label	θ_222_/θ_208_ (PBS)	MRE^a^ θ_222_ (PBS)	MRE θ_222_ (50 or 60% TFE/PBS)	% helicity	*K*_d_ (μM) from FP	*K*_d_ (μM) from FQ	half-life (h)^b^
linear	0.19	–3312	–23 827	14	>5	3.96 ± 0.86	0.8 (0.7–0.9)
BAMM	0.47	–5586	–20 944	27	4.65 ± 2.46	0.51 ± 0.10	2.1 (1.6–3.2)
BSM	0.51	–5002	–15 468	32	4.92 ± 1.79	1.61 ± 0.44	2.6 (2.0–3.7)
dual	1.19	–11 273	–20 083	56	0.35 ± 0.06	0.07 ± 0.03	39.5 (>19.3)
staple-1	0.88	–13 179	–19 228	69	0.13 ± 0.04	0.10 ± 0.03	5.7 (4.8–7.0)
staple-2	0.93	–11 053	–20 473	54	0.24 ± 0.10	0.14 ± 0.05	7.0 (5.2–10.9)

a MRE: mean-residue ellipticity.

b 95% confidence interval in parentheses.

Aggregation of dual peaks was observed via CD as described earlier. Its multimeric state at 0.4 mM was confirmed by analytical ultracentrifugation (AUC) in 20% DMSO/PBS (DMSO cosolvent for solubilization; concentrations are necessarily high in this technique). Other work indicates native structures are not perceivably impacted at similar DMSO concentrations.^56−59^ In 20% DMSO/PBS, 90% dual oligomerized into a mixture of dimer, tetramer, and hexamer (1:4:3) (Table S2). Dimer/tetramer and trimer/hexamer peaks were also observed in the MS spectra of the dual samples (Figure S27 and characterization).

### Conformation of Dual by NMR

NMR data for dual unambiguously showed it adopts one predominant helical conformation in 35% TFE/H_2_O. That solvent ratio was chosen by (i) titrating TFE into a PBS solution of 17 μM dual to establish the minimum TFE to eliminate aggregation (Figure S5) and (ii) comparing peak separations in 1D ^1^H–^1^H spectra of 4 mM dual in 20–35% TFE/H_2_O (Figure S12). These experiments point to a threshold of 30% TFE, so 35% TFE was used for the NMR experiments to allow for potential deviations in these measurements.

**Figure 4:**
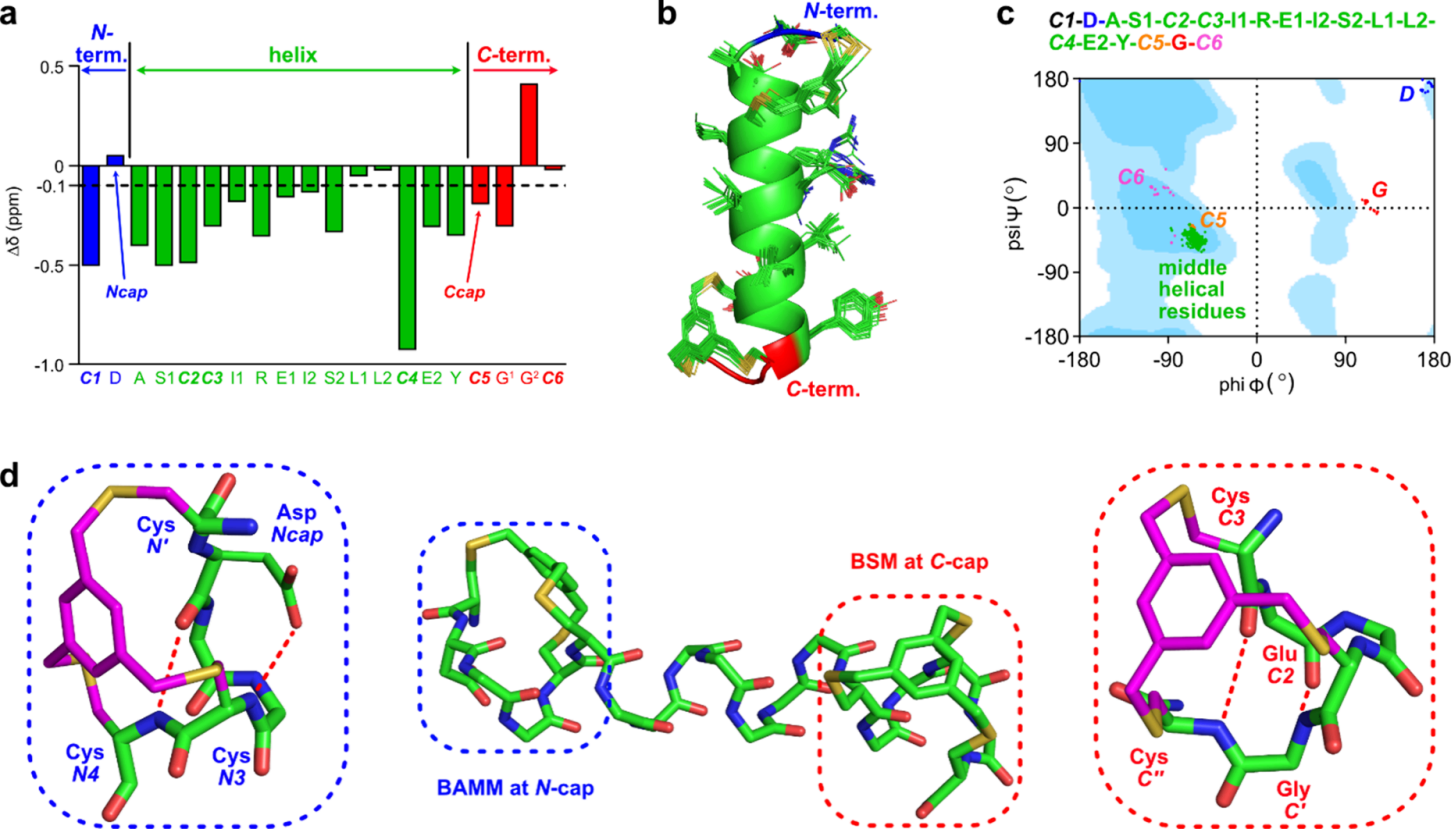
(a) CSIs of dual *C*-helix, showing transitions between the *N*-cap (blue) to helix (green) to *C*-cap (red). (b) 31 low-energy conformers of dual. (c) Ramachandran plot of backbone dihedrals in dual low-energy conformers (favored and allowed φ,ψ in all protein conformations (excluding Pro and Gly) shown in deep and light blue backgrounds). (d) Lowest-energy conformer with BAMM *N*-cap (blue dotted boxes) and BSM *C*-cap (red) in the dual peptide.

Helical residues are recognized by chemical shift index (CSI) studies.^60^ Dual was robustly helical by this metric because nearly all the putative helical residues (A–Y in green) have Δδ values <−0.1 ppm (Figure 4a), the only exception being L1 and L2 (leucines have been noted to give high Δδs in reported helical peptides/peptidomimetics^41,61^). Stark differences were observed between Δδs for the putative helical region in comparison to the nonhelical residues beyond the *N-* and *C-*caps (blue and red, respectively, in Figure 4a). Residues ***C1*** and ***C4*** had high negative Δδs, consistent with shielding of their *CαH̲* atoms in the TMB anisotropic field in BSMs^46^ and BAMMs^53^ (Figure S23).

Dual was also shown to be unambiguously helical after conformational sampling constrained by distances deduced by 2D NMR. Only one cluster was observed within 3 kcal·mol^–1^ of the global minimum and all conformers under this threshold overlaid tightly (Figure 4b). A Ramachandran presentation (Figure 4c) shows putative helical residues are in the ideal {right-handed} α-helix region (A–Y in green), while the nonhelical ones are outside it, as expected (*N*-terminal D {blue}, *C*-terminal G {red}, and *C***6** {magenta}). By definition, the peptide fragment projecting from the *C*-terminus must “turn back” on the helix in Schellman loops; consistent with this, the *C′* residues (Gly in this case) are uniformly in the *left-handed* region on Ramachandran plots.

Figure 4d shows the lowest-energy conformation of dual. Expansions show a natural *N*-cap (ASX motif) and a natural *C*-cap (Schellman loop) are constrained within the BAMM and BSM bicycles. These serve as two helix-inducing nuclei at both termini to constantly enforce helicity for the peptidomimetics.

### Binding to Cyclins

**Figure 5:**
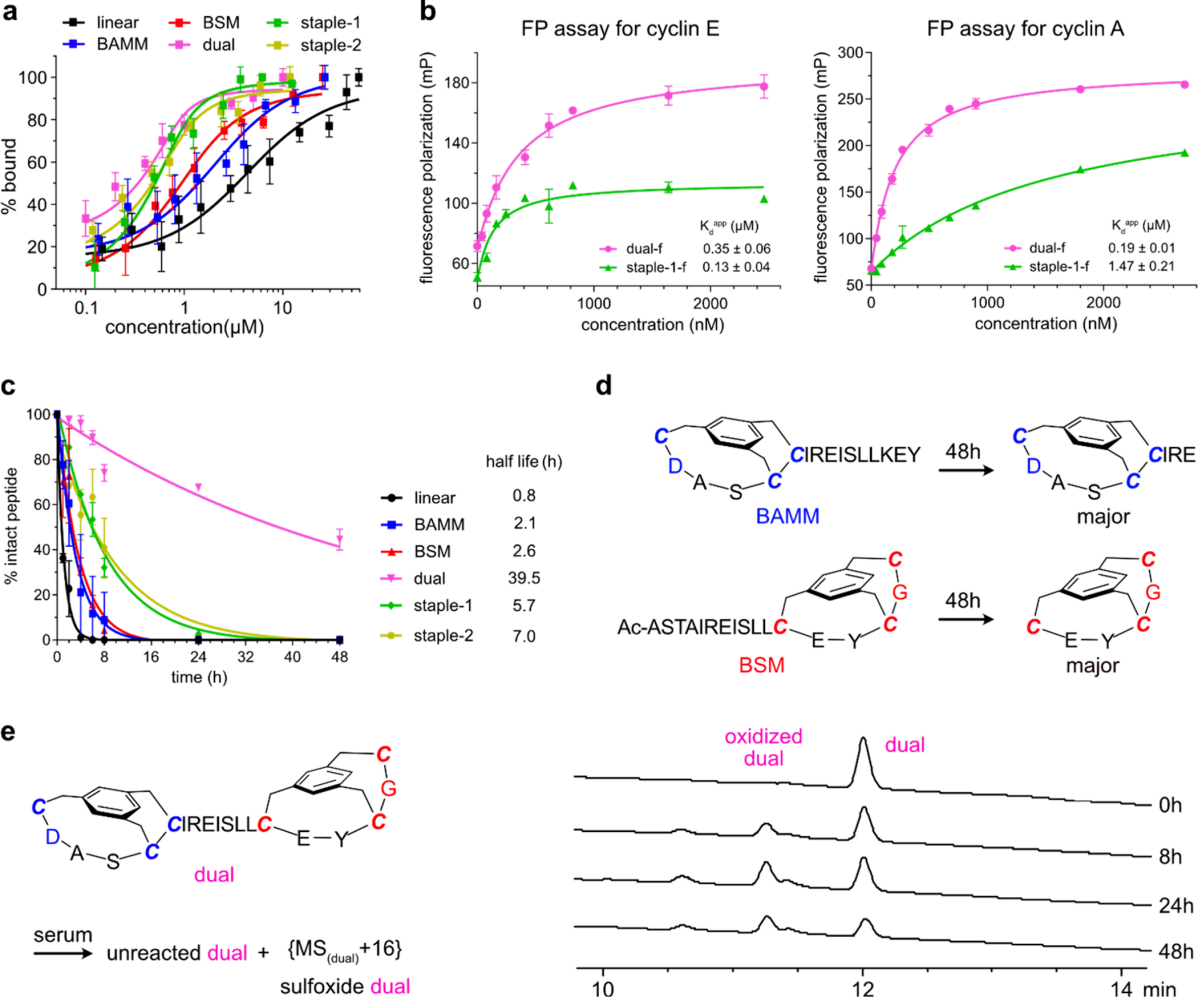
(a) FQ binding data for six unlabeled peptides/peptidomimetics with cyclin E. (b) FP binding of dual-f and staple-1-f by cyclins E and A1. (c) Mimic degradation profiles in 25% human serum at 37 °C. (d) Major degradation products of BAMM and BSM-capped peptides after 48 h. (e) Degradation process of dual-capped *C*-helix in 48 h and corresponding analytical HPLC traces at 0, 8, 24, and 48 h.

Fluorescence polarization and quenching assays for binding of the featured compounds with cyclin E (FP and FQ, Figures 5a,b and S30) showed the same trends. Linear bound weakly, BAMM, and BSM had lower *K*_d_ (3.96, 0.51, 1.61 μM, respectively, FQ), while staples 1, 2 and dual had the best affinities (0.10, 0.14, 0.07, μM, respectively, FQ). CDK2 is known to bind predominantly to cyclins A and E.^62,63^ Cyclin A1, unlike E, does not have an interface Trp, so analogous FQ assays are not possible, but FP using fluorescein labeled peptides (dual-f, *etc.*) was performed. In these FP experiments, dual showed similar affinities for cyclins E (center) and A1 (right). Staple 1 bound cyclin E with higher affinity and A1 with 10-fold lower affinities (Figure 5b).

### Serum Stabilities

The test compounds were separately incubated with 25% human serum at 37 °C; residual starting materials were monitored as a function of time, and degradation products were characterized using LC-MS (Figure 5c). Linear rapidly degraded to its constituent amino acids, whereas BSM and BAMM hydrolyzed more slowly and formed stable byproducts comprising the BSM bicycle or the BAMM bicycle with three residues (Figure 5d). Staples 1 and 2 had overall higher stabilities but lesser protection than BAMM at the *N*- and BSM at the *C*-terminus. This is proved by their identified metabolic products where proteolysis happened at both termini. Besides terminal degradation, we also observed ring-opening products by hydrolysis of amide bonds within the hydrocarbon macrocycles in two stapled peptides (Figures S28, S29). This suggested that the hydrocarbon macrocycles had some flexibility to adapt to the cleavage sites of proteases and could be cut from the middle.

Dual unambiguously was the most stable in the series: it had the longest half-life, *t*_1/2_ > 49× the linear control, and >5.6× that of the next most stable peptidomimetic, staple-2. Ultimately, oxidation (sulfoxides, M^+^+16) but not proteolysis was observed (LC-MS) in these experiments, probably due to oxygen or reactive oxidative species (Figure 5e). These dual-staple differences are important because drug approval often depends on characterization and toxicity assays for dominant metabolites; this would be less arduous for dual metabolites than staples.

### Cell Uptake

**Figure 6:**
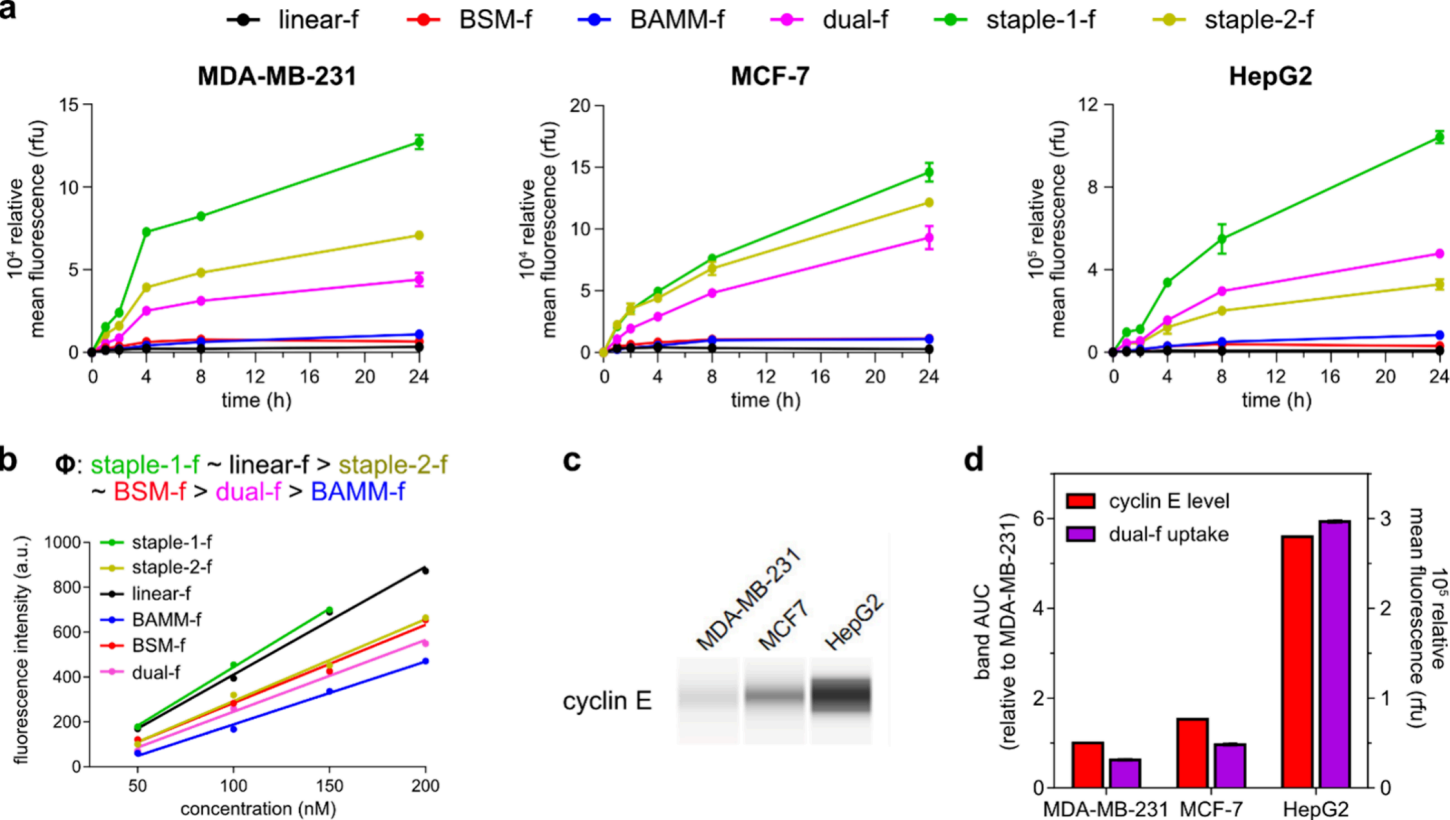
(a) Cell uptake into three cell lines (MDA-MB-231, triple negative breast cancer; MCF-7, ER^+^ breast cancer; HepG2, liver cancer) after incubation of 1 μM test compounds at 37 °C, as a function of time. (b) Fluorescence spectra as a function of concentrations indicate a difference in quantum yields corresponding to variable fluorescein environments. (c) Western blot of cyclin E shows relative levels of expression in those three cell lines. (d) Comparison of levels of cyclin E expression with uptake of the dual peptide for the three cell lines.

FITC-conjugated derivatives of the six peptides were prepared, and relative levels of uptake of these compounds into three cell lines were measured via fluorescence activated cell sorting (FACS; Figure 6a). Linear (black line) showed minimal uptake, while BAMM (blue) and BSM (red) permeated slightly better. However, staple-1 (green), -2 (gold), and dual (pink) were taken up significantly more. Based on the fluorescence intensity in Figure 6a, it appears staple-1 was uptaken ∼2× better than dual, but the actual difference is less considering their difference in quantum yields shown in Figure 6b. Relative levels of cyclin E expression in the three cell lines correlated to the observed uptake levels (Figure 6c,d). This is a possible indicator of protein-target driven cell uptake; *i.e.*, cyclin E tends to hold the compounds in the cells.

## Conclusions

**Figure 7:**
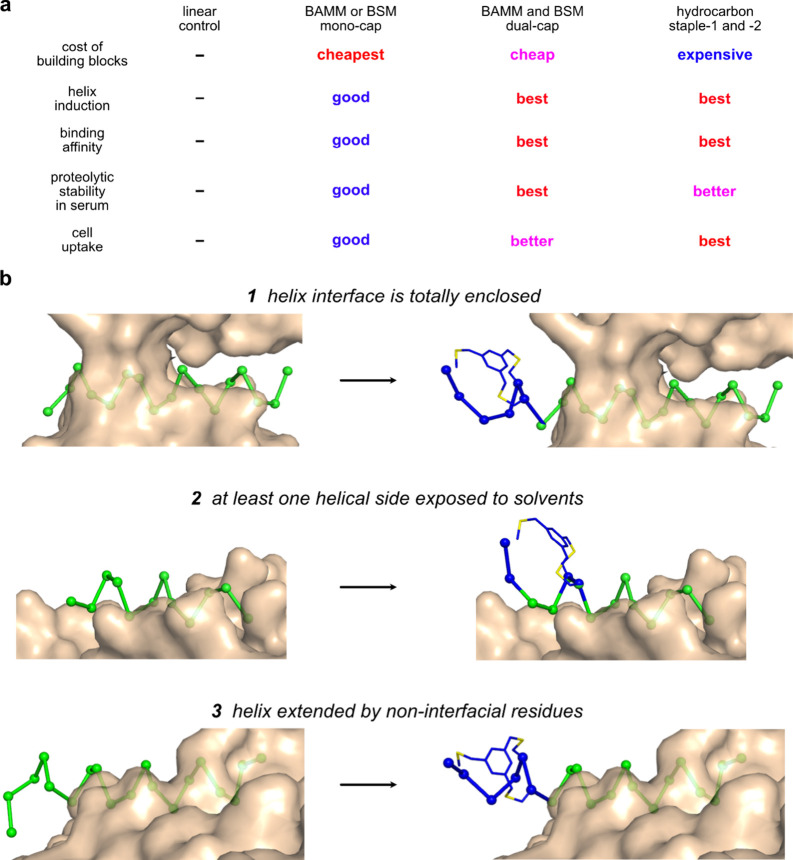
(a) Comparisons between linear, BAMM or BSM monocapped, BAMM/BSM dual-capped, and hydrocarbon-stapled peptides in terms of cost and biophysical properties. (b) Helices in protein ligands can be totally enclosed by the receptor, exposed on one flank, or extended beyond the receptor. BAMM *N*-caps, for instance, can be used in all three situations, as described in the text. Green for native helices; blue for added or mutated residues or fragments to install BAMM *N*-caps.

We had correctly hypothesized that dual would have better helicity, serum stability, cyclin E binding, and cell uptake when compared with the monocapped systems BAMM and BSM. NMR studies showed the dual to be unambiguously helical in a TFE/H_2_O medium (Figure 4). Figure 7a graphically summarizes findings when dual was also compared to two gold standard stapled helices; all three gave good helical inductions, binding affinities to cyclin E, and cell uptake, and differences within that series were relatively small. However, proteolytic stabilities in serum were conspicuously higher for dual than staples 1 and 2.

Dual-capped helical peptidomimetics have no constraint on amino acid compositions or peptide length. They are more conveniently prepared than hydrocarbon-stapled peptides, because only cheap, orthogonally protected Cys as building blocks are required. Further, they should not be contaminated with heavy metal residues such as ruthenium (used to staple or cap via metathesis reactions). Consequently, if no other modifications were made, dual peptidomimetic would be the first-choice peptidomimetic for helix mimicry. Moreover, dual-capping and staples are orthogonal helix-mimicry strategies, so it is possible to apply both on long and complicated sequences.

Figure 7b shows three situations of interface helices binding their receptors (left) and how to install BAMM *N*-caps on these helices, respectively (right, blue for added or mutated fragments). Helices in protein ligands can (1) be totally enclosed by the receptor, (2) be exposed to solvent on at least one side, or (3) extend beyond the receptor. Situations in (1) are rare; the design strategy for these might be to install an additional BAMM *N*-cap at the *N*-terminus of the helix. More commonly, ligand helices bind to receptors using one face and leave the other exposed as in (2); then two *N*-terminal, solvent-exposed residues at *N3* and *N4* may be mutated to Cys. Posthelical residues *N′* (Cys) and *Ncap* (Asp) could be added at the *N*-terminus. The three Cys at *N′*, *N3*, and *N4* could then react with TBMB to form a BAMM *N*-cap. In situations such as *(3)*, the natural overhangs may be directly replaced by BAMM *N*-caps. Similar situations may happen at peptide *C*-termini, and BSM *C*-caps could be installed via similar designs; hence, there are ways to incorporate BAMM and BSM cassettes to nearly all interface helices.

In summary, we assert it is now possible to dual cap helical mimics, and the advantages of dual capping as presented are numerous. To be clear, dual-capped mimics to perturb some PPIs may work better than others but no methodology of this type is uniformly applicable. Tests of BAMM/BSM dual capping on different sequences are encouraged to further evaluate the strengths and weaknesses of this methodology. A potential problem is aggregation or undesirable hydrophobic interactions derived from the TMB group. Other capping groups with the same symmetry but greater hydrophilicity could be investigated to mitigate hydrophobicity or a BSM could be used in conjunction with an *N*-cap mimic (Figure 1b) other than a BAMM. Studies of this kind are underway in our laboratory.

## Supporting Information Available

Details of peptide syntheses in section B; CD experiments in section C; SV-AUC (sedimentation velocity analytical ultracentrifugation) experiments in section D; MD simulations in section E; hot spot analyses in section F; NMR experiments with spectra and distance constraints in section G; serum stability assays in section H; fluorescence polarization assays in section I; fluorescence quenching assays in section J; cellular experiments in section K; characterization of peptides in section l (PDF)
Peptide movie clips (BAMM17, BSM17, Dual17, L17) (ZIP)
Peptide movie clips (HBS17, staple17, strap17) (ZIP)
NMR solution ensembles of dual C-helix (PDB)

## Supplementary Material

ja3c11717_si_003.zip

ja3c11717_si_004.pdb
