# Supplementary material for: Dual-Capped Helical Interface Mimics
Source: J Am Chem Soc. 2024 Apr 4;146(15):10331–41. doi: 10.1021/jacs.3c11717 (PMC11027154; doi:10.1021/jacs.3c11717)
Supplement: Supplementary file 1 — ja3c11717_si_001.pdf [file ja3c11717_si_001.pdf]

Dual Capped Helical Interface Mimics

Tianxiong Mi[1], Zhe Gao[1], Zeynep Mituta[2] and Kevin Burgess[1]*

[1]Department of Chemistry, Texas A & M University, Box 30012, College Station, TX 77842, USA

[2]ZentriForce Pharma Research GmbH, Carl-Friedrich-Gauss-Ring 5, 69124 Heidelberg, Germany

Email: burgess@tamu.edu

Table of Contents

A. Abbreviations **3**
B. Syntheses of Peptides **4**
Linear Peptides 4
Bicyclic *C*-Capped Peptides 5
Bicyclic *N*-Capped Peptides 6
i – *i*+4 Hydrocarbon Stapled Peptides 7
Fluorescently Labeled Peptides 7
BAMM/BSM Dual Capped Peptides 8
One-Pot Reaction in Solution Phase 8
Solid Phase CLIPS 8
Dual-capped Peptide Synthesis: BSM on Solid, BAMM in Solution 10
C. Circular Dichroism (CD) Experiments **12**
CD Spectra of Helical C-helix Peptides in PBS at Different Concentrations 12
CD Spectra of C-helix Dual in Varying Ratios of TFE/PBS Solution 13
CD Spectra of C-helix Peptides at Maximal Helical States in TFE/PBS Buffer 14
Calculation of Percent Helicity 15
D. Sedimentation Velocity Analytical Ultra-centrifugation (SV-AUC) Experiments on C-helix Dual **16**
SV-AUC measurement results 17
E. Molecular Dynamics (MD) on Modeled Ala-rich 17-mers **18**
F. Solvent Accessible Surface Area (SASA) Analysis on C-helix in CDK2 - cyclin E **20**
G. NMR Experiments **21**
1D and 2D NMR Spectroscopy 21
C-helix Dual 21
Best Solvents for NMR Experiments 21
NMR Spectra 22
Peak Assignment 30
Chemical Shift Index (CSI) Calculation 31
Distance and Dihedral Constraints 31
NOE Summary 38
Ramachandran Plot of (θ, ψ) Angles 39
The Impacts of Shielding Effect from TMB to Nearby Hs 40
H. In Vitro Peptide Stability in Human Serum **41**
RP-HPLC Spectra and Potential Degradation Products of C-helix Peptides 41
I. Fluorescence Polarization Assay **47**
The Procedure of Direct FP assay to Measure K_d of Fluorescent Peptides 47
J. Fluorescence Quenching Assay **49**
K. Cell Culture and Cellular Uptake Assay **50**
Cell Culture 50
Cellular Uptake Assay 50
Confocal Imaging 50
Blotting Experiments for Cyclin E 52
Fluorescence Spectra of Tested Peptides from 50 to 200 nM 53
L. Characterization of Purified Peptides **54**
M. References **72**

A. Abbreviations

TLC: thin layer chromatography

prepHPLC: preparation high performance liquid chromatography

MeCN: acetonitrile

analyHPLC: analytical high performance liquid chromatography

ESI-MS: electrospray ionization mass spectrometry

Fmoc: fluorenylmethoxycarbonyl

DIPEA: N,N-diisopropylethylamine

DMF: dimethylformamide

Oxyma: ethyl cyanohydroxyiminoacetate

DIC: N,N'-diisopropylcarbodiimide

HOBt: hydroxybenzotriazole

NMM: N-methylmorpholine

TFA: trifluoroacetic acid

TFE: 2,2,2-trifluoroethanol

TIPS: triisopropylsilane

DMSO: dimethyl sulfoxide

PBS: phosphate-buffered saline

MCMM: Monte Carlo Multiple Minimum

TBMB: 1,3,5-tris(bromomethyl)benzene

TMB: 1,3,5-trimethylbenzene

pA: (S)-2-(4-pentenyl)Ala

AA：amino acid

DCE: 1,2-dichloroethane

B. Syntheses of Peptides

Linear Peptides

The linear control peptides were synthesized using standard Fmoc peptide synthesis protocols (Scheme S1) on TentaGel S RAM resin (capacity = 0.22 mmol/g) on a LibertyBlue peptide synthesizer. Couplings were carried out using 5 equivalents (equiv) of Fmoc-protected amino acid, 10 equiv of Oxyma, 10 equiv of DIC. Coupling reactions were allowed to proceed for 2 minutes in DMF at 90 ℃, after which Fmoc deprotection was carried out with 20% (vol/vol) piperidine in DMF at 90 ℃ for 1 minutes. Upon completion of the peptide sequence assembly on resin and deprotection of the final Fmoc group, the *N*-terminal amine was acetylated by treatment with 25% (vol) acetic anhydride and 3.5% (vol) DIPEA in DMF. Cleavage and deprotection from the solid support was carried out using TFA/H2O/TIPS (95/2.5/2.5 vol/vol) for 3 hours at room temperature. The resin was then filtered and washed with excess TFA. The isolated TFA solution was then evaporated, and the peptide was precipitated by adding cold diethyl ether. The peptide suspension in ether was centrifuged to pellet followed by decanting the ether. The pellet was then washed twice with cold diethyl ether. Purification of the resulting peptides was achieved by high performance liquid chromatography (HPLC) on a reversed-phase C8 column to yield peptide with purity >95%. Purity and identity were assessed using ESI-MS and analytical HPLC on a C18 column.

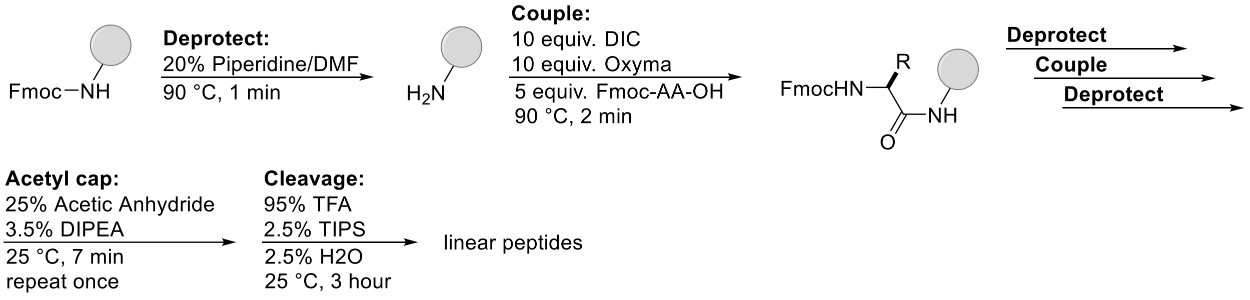

Scheme S1 Synthesis route for linear peptides.

Bicyclic *C*-Capped Peptides

The linear peptides were synthesized using standard Fmoc peptide synthesis protocols (Scheme S1) on TentaGel S RAM resin (capacity = 0.22 mmol/g) on a LibertyBlue peptide synthesizer. The three cystines were placed at *C3*, *Ccap* and *C''* near the *C*-terminus. Couplings were carried out using 5 equivalents (equiv) of Fmoc-protected amino acid, 10 equiv of Oxyma, 10 equiv of DIC. Coupling reactions were allowed to proceed for 2 minutes in DMF at 90 ℃, after which Fmoc deprotection was carried out with 20% (vol/vol) piperidine in DMF at 90 ℃ for 1 minutes. Upon completion of the peptide sequence assembly on resin and deprotection of the final Fmoc group, the N-terminal amine was acetylated by treatment with 25% (vol) acetic anhydride and 3.5% (vol) DIPEA in DMF. Cleavage and deprotection from the solid support was carried out using TFA/H2O/TIPS (95/2.5/2.5 vol/vol) for 3 hours at room temperature. The resin was then filtered and washed with excess TFA. The isolated TFA solution was then evaporated, and the peptide was precipitated by adding cold diethyl ether. The peptide suspension in ether was centrifuged to pellet followed by decanting the ether. The pellet was then washed twice with cold diethyl ether and dissolved in 1:1 mixture of aqueous buffer (20mM NH_4HCO_3) and ACN to make final concentration around 1 mM. 1.5 equiv of TBMB was added and the solution was stirred for 15 min at room temperature. Purification of the resulting peptides was achieved by high performance liquid chromatography (HPLC) on a reversed-phase C8 column to yield peptide with purity >95%. Purity and identity were assessed using ESI-MS and analytical HPLC on a C18 column.

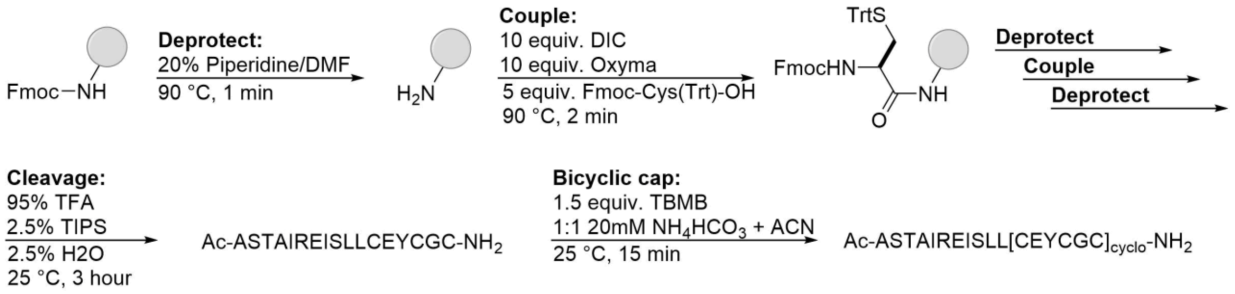

Scheme S2 Synthesis route for bicyclic *C*-capped peptides.

Bicyclic *N*-Capped Peptides

The linear peptides were synthesized using standard Fmoc peptide synthesis protocols (Scheme S1) on TentaGel S RAM resin (capacity = 0.22 mmol/g) on a LibertyBlue peptide synthesizer. The three cystines were placed at *N'*, *N3* and *N4* at the *N*-terminus. Couplings were carried out using 5 equivalents (equiv) of Fmoc-protected amino acid, 10 equiv of Oxyma, 10 equiv of DIC. Coupling reactions were allowed to proceed for 2 minutes in DMF at 90 °C under microwave, after which Fmoc deprotection was carried out with 20% (vol/vol) piperidine in DMF at 90 °C for 1 minutes. Upon completion of the peptide sequence assembly on resin and deprotection of the final Fmoc group, cleavage and deprotection from the solid support was carried out using TFA/H_2O/TIPS (95/2.5/2.5 vol/vol) for 3 hours at room temperature (longer time is required for peptides with Arg). The resin was then filtered and washed with excess TFA. The isolated TFA solution was then evaporated, and the peptide was precipitated by adding cold diethyl ether. The peptide suspension in ether was centrifuged to pellet followed by decanting the ether. The pellet was then washed twice with cold diethyl ether and dissolved in 1:1 mixture of aqueous buffer (20mM NH_4HCO_3) and ACN to make final concentration around 1 mM. 1.5 equiv of TBMB was added and the solution was stirred for 15 min at room temperature. Purification of the resulting peptides was achieved by high performance liquid chromatography (HPLC) on a reversed-phase C18 column to yield peptide with purity >95%. Purity and identity were assessed using ESI-MS and analytical HPLC on a C18 column.

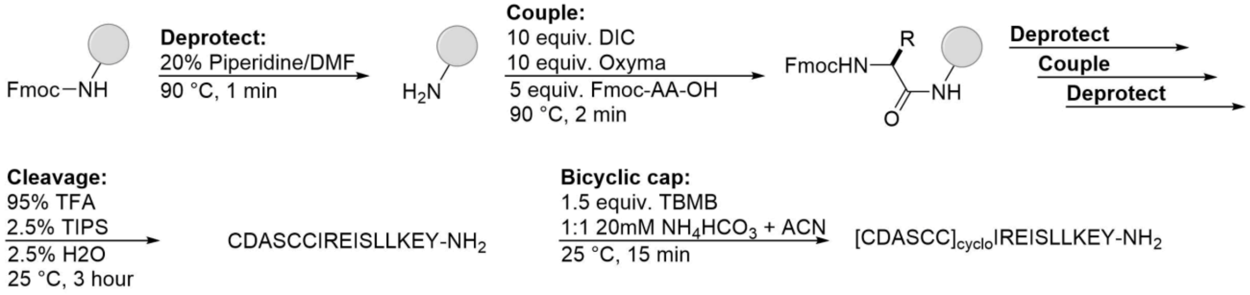

Scheme S3 Synthesis route of bicyclic *N*-capped peptides.

i – *i*+4 Hydrocarbon Stapled Peptides

The linear peptides were synthesized using standard Fmoc peptide synthesis protocols (Scheme S1) on TentaGel S RAM resin (capacity = 0.22 mmol/g) on a LibertyBlue peptide synthesizer. (S)-2-(4-pentenyl)Ala (pA) was used at chosen *i* and *i*+4 positions to mutate original non-interactive amino acids. Couplings were carried out using 5 equivalents (equiv) of Fmoc-protected amino acid, 10 equiv of Oxyma, 10 equiv of DIC. Coupling reactions were allowed to proceed for 2 minutes in DMF at 90 ℃ under microwave, after which Fmoc deprotection was carried out with 20% (vol/vol) piperidine in DMF at 90 ℃ for 1 minutes. Double coupling was conducted for the coupling of (S)-2-(4-pentenyl)Ala (pA) and the next residue of it. Upon completion of the peptide sequence assembly on resin, olefin metathesis were done following the published protocol[1]. Cleavage and deprotection from the solid support was carried out using TFA/H_2O/TIPS (95/2.5/2.5 vol/vol) for 3 hours at room temperature (longer time is required for peptides with Arg). The resin was then filtered and washed with excess TFA. The isolated TFA solution was then evaporated, and the peptide was precipitated by adding cold diethyl ether. The peptide suspension in ether was centrifuged to pellet followed by decanting the ether. Purification of the resulting peptides was achieved by high performance liquid chromatography (HPLC) on a reversed-phase C18 column to yield peptide with purity >95%. Purity and identity were assessed using ESI-MS and analytical HPLC on a C18 column.

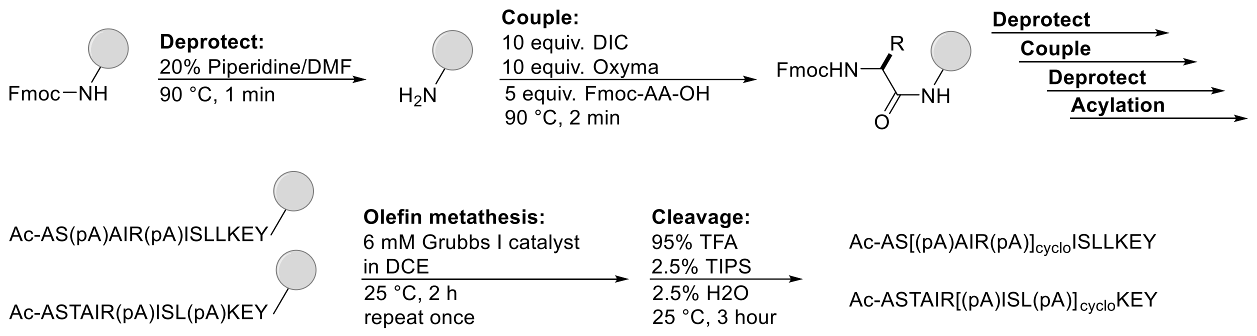

Scheme S4 Synthesis route of hydrocarbon stapled peptides.

Fluorescently Labeled Peptides

FITC was conjugated with peptides to impart fluorescence. After deprotection of last Fmoc on solid phase and thorough wash with DMF and DCM, a 3 mL DMF solution with 7 eq. FITC and 14 eq. DIPEA was added to the syringe at room temperature for 16 hours. After another round of thorough wash with DMF and DCM, FITC-peptides were cleaved from the resin by TFA/H_2O/TIPS (95/2.5/2.5 vol/vol) for 3 hours at room temperature. Following protocols are the same as the above ones.

BAMM/BSM Dual Capped Peptides

One-Pot Reaction in Solution Phase

Our first attempt to make the dual-capped peptide is to cyclize the linear peptide with six free Cys simultaneously in solution. The linear peptide bearing six Cys was synthesized following the protocols above. After cleavage from resin, it was immediately dissolved in a 1:1 mixture of aqueous buffer (20mM NH_4HCO_3) and ACN, and then 3 eq. TBMB was added to the solution. After 15 min, the reaction mixture was checked by LCMS, and analytical HPLC (25 min from 10% acetonitrile {0.1% TFA} + 90% H_2O {0.1% TFA} to 90% acetonitrile {0.1% TFA} + 10% H_2O {0.1% TFA}, plus 5 min wash at 10% acetonitrile {0.1% TFA} + 90% H_2O {0.1% TFA}, same conditions for analytical HPLC traces in this section). The mixtures showed multiple peaks. This observation suggests the intra-cap cyclization was probably not fast enough to cleanly form two isolated bicyclic rings for this tested sequence.

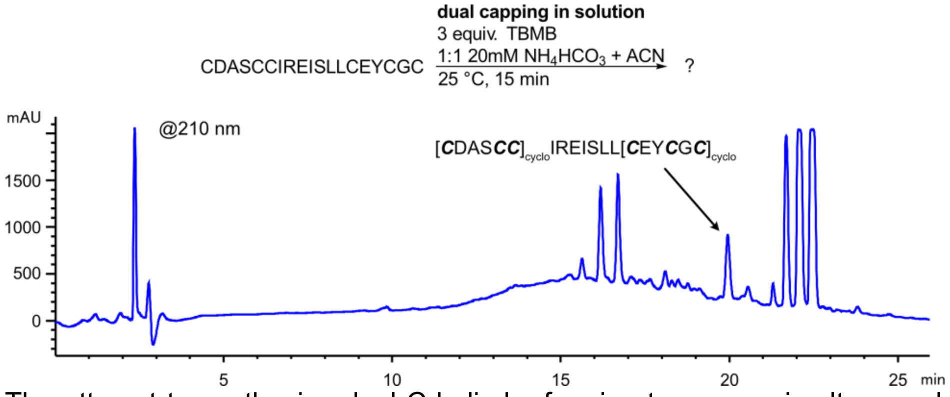

Figure S1 The attempt to synthesize dual C-helix by forming two caps simultaneously in solution. Bottom shows analytical HPLC trace of the reaction mixtures after 15 min.

Solid Phase CLIPS

CLIPS reactions were frequently done in solution. We only found one reference with a vague protocol of solid phase CLIPS [2]. Instead of Cys(Trt), Cys(Mmt) was used to facilitate selective on-bead deprotection. We followed published protocols to selectively deprotect Mmt on resin[3], but optimized some specific ratios and amounts to make it best suitable for our system. Specifically, a solution of 1% TFA/DCM was prepared. 2 mL solution was added to the syringe, shaking for 2 min, then drained. This procedure was repeated, and the solutions normally would change from yellow, to orange, to red, then back to orange, and finally to light yellow after around 10 to 15 rounds of deprotections. After that, the beads are washed with several rounds of DCM and DMF. For solid phase cyclization, a 3 mL DMF solution with 2 eq. TBMB and 10 eq. DIPEA are prepared, then added to the syringe, and reacted for 1 hour, followed by thorough wash with DMF and DCM before the next step.

This protocol was applied on the syntheses of solid-phase *C*-cap BSM and *N*-cap BAMM, respectively, to evaluate CLIPS efficiency on different helical termini. Analytical HPLC traces of crude peptides (after cleaved off the resin) were shown in the diagrams below. We found relatively clean transitions from the linear to bicyclic capped peptide for the *C*-terminal CLIPS, but not so for *N*-terminal CLIPS. This may be rationalized below. The peptide sequences were linked to resin on their *C*-termini, so *C*-terminal Cys are less flexible, hence hard to contact Cys of

another chain to cause inter-chain cross-linking. On the other hand, the *N*-terminal Cys are away from the resin, more flexible, so they are easier to contact Cys of another chain, resulting in messy cross-linking products upon addition of CLIPS reagents (TBMB). This is shown in Fig S2**c**.

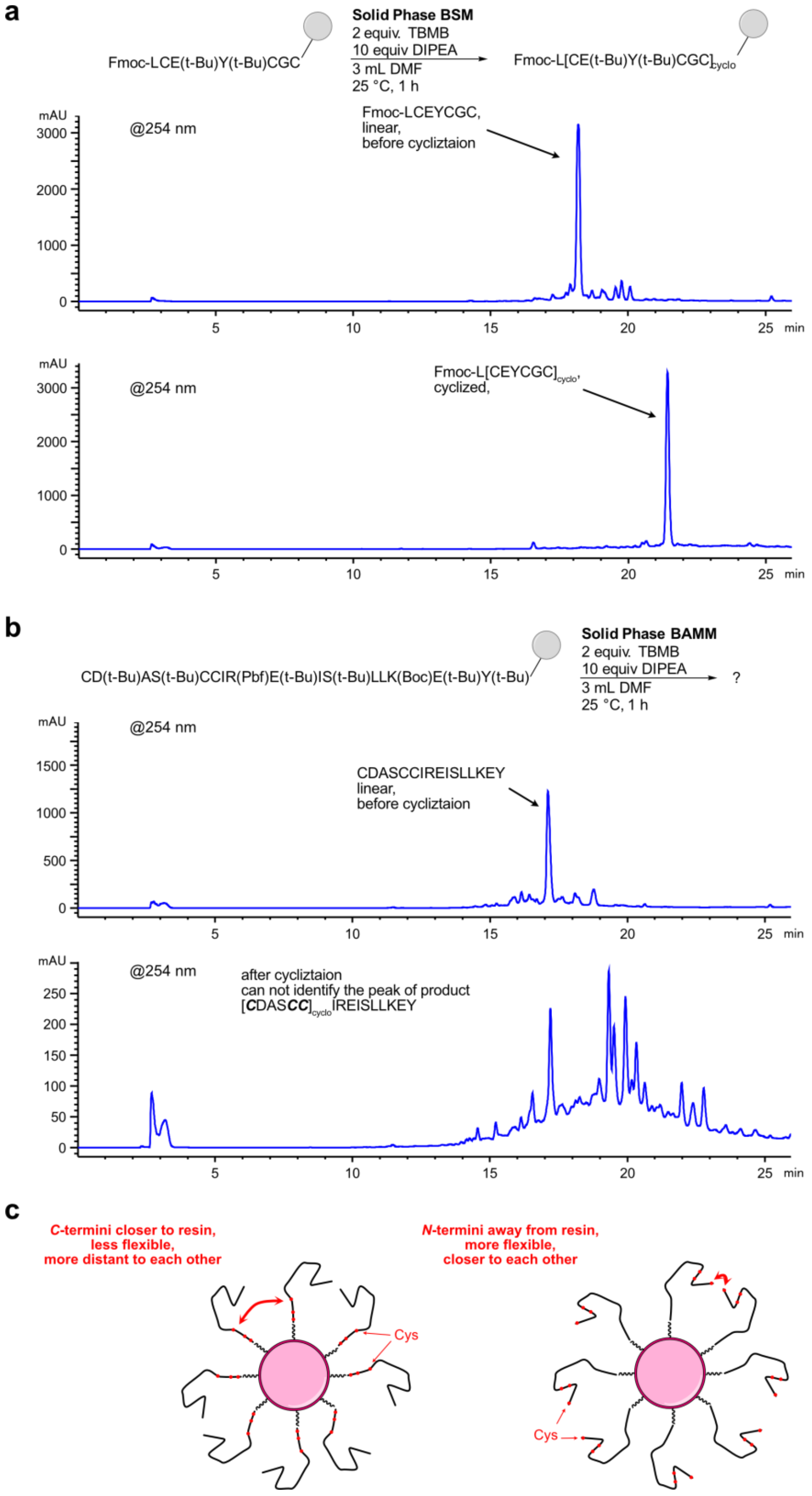

Figure S2 Formation of **a** BSM *C*-cap and **b** BAMM *N*-cap on solid phase with crude analytical HPLC traces of starting linear peptides and reaction mixtures after cyclization; **c** Cartoon graphics showing potential difference of resin-linked *C*- and *N*-terminal Cys groups.

Dual-capped Peptide Synthesis: BSM on Solid, BAMM in Solution

The experience above naturally guided us to a route which synthesized the two bicyclic caps at different stages. More specifically, the bicyclic *C-cap*, BSM, was first synthesized on solid phase, while the bicyclic *N*-cap, BAMM, was later synthesized in solution.

Here are detailed procedures. The seven residues at the *C*-termini, including six residues forming BSM and the one before it, were first coupled on the resin, either manually or automatically using peptide synthesizer. Here Cys were in the form of Cys(Mmt). The Fmoc group was intact for the last residue, and then selective deprotection of Mmt followed by solid phase CLIPS was conducted to form BSM *C*-cap on solid phase. After thorough wash by DCM and DMF, solid phase peptide synthesis was continued to grow the rest amino acids on the short *C*-terminal sequence. Cys at the *N*-termini were in the form of Cys(Trt). After final cleavage from resin and precipitation in ether, peptides were dissolved in solution for solution phase CLIPS to build BAMM *N*-cap. Purification of the resulting peptides was achieved by high performance liquid chromatography (HPLC) on a reversed-phase C18 column to yield peptide with purity >95%. Purity and identity were assessed using ESI-MS and analytical HPLC on a C18 column.

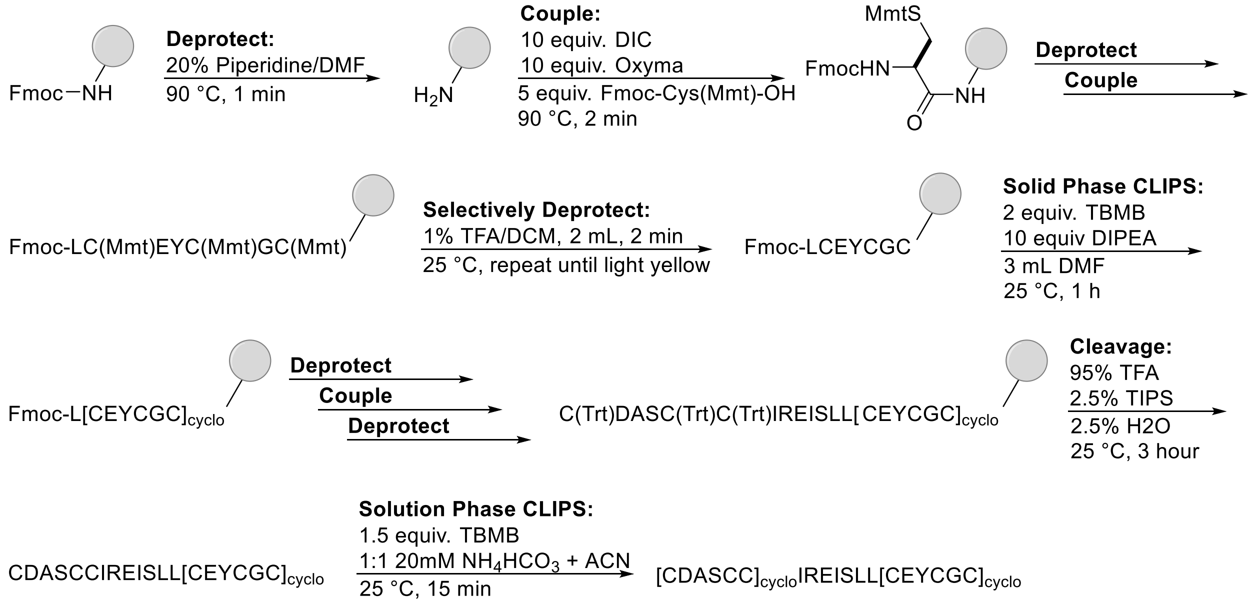

Scheme S5 Synthesis route of dual C-helix; residues E, Y, D, S, R were in the form of E(t-Bu), Y(t-Bu), D(t-Bu), S(t-Bu), R(Pbf) before cleavage in 95% TFA solution.

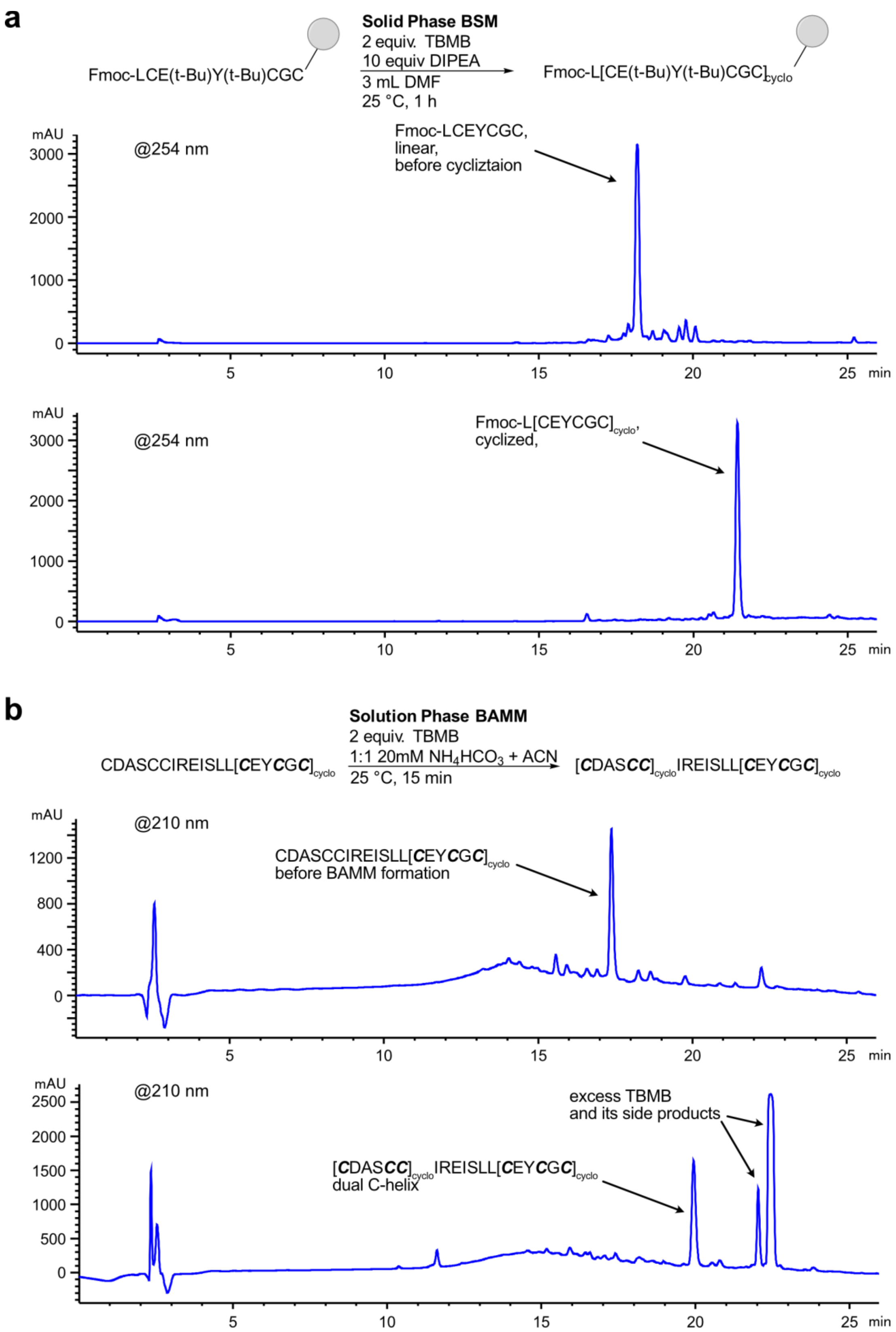

Figure S3 Formation of **a** BSM *C*-cap on solid phase and **b** BAMM *N*-cap in solution phase for the synthesis of dual C-helix. Crude analytical HPLC traces of starting peptides and reaction mixtures were shown under the reaction schemes.

C. Circular Dichroism (CD) Experiments

Concentrations of the stock solution were determined by 275 nm absorption of Tyr with ε = 1455 ($M^{-1}cm^{-1}$) for linear and hydrocarbon stapled peptides, (1455+555 =) 2010 ($M^{-1}cm^{-1}$) for BAMM and BSM mono-capped peptides, and (1455+555*2 =) 2565 ($M^{-1}cm^{-1}$) for the dual-capped peptide; 555 ($M^{-1}cm^{-1}$) is the 275 nm absorbance coefficient of thiol-alkylated TMB as shown in main text Figure 3. Each sample was dissolved in PBS (pH 7.4) or TFE/PBS with the final concentration in the range of 5 to 30 μM. CD spectra were acquired using circular dichroism spectrometer (Chirascan) equipped with a temperature controller using 1 mm cell at a scan speed of 0.5 nm/sec at indicated temperature. Each sample was scanned three times and the averaged spectrum was smoothed.

CD Spectra of Helical C-helix Peptides in PBS at Different Concentrations

To test if helical peptides, including dual, staple-1 and staple-2, aggregated within concentrations we used in this study, concentration-dependent CD studies were facilitated. Each peptide was dissolved in PBS buffer to prepare samples with concentrations in the range of 5 to 30 μM. Samples were left for 1 h at 25 °C and then tested in CD experiments, and their mean-residue molar $\theta_{[222]}$ were plotted against their concentrations to evaluate oligomeric behaviors.

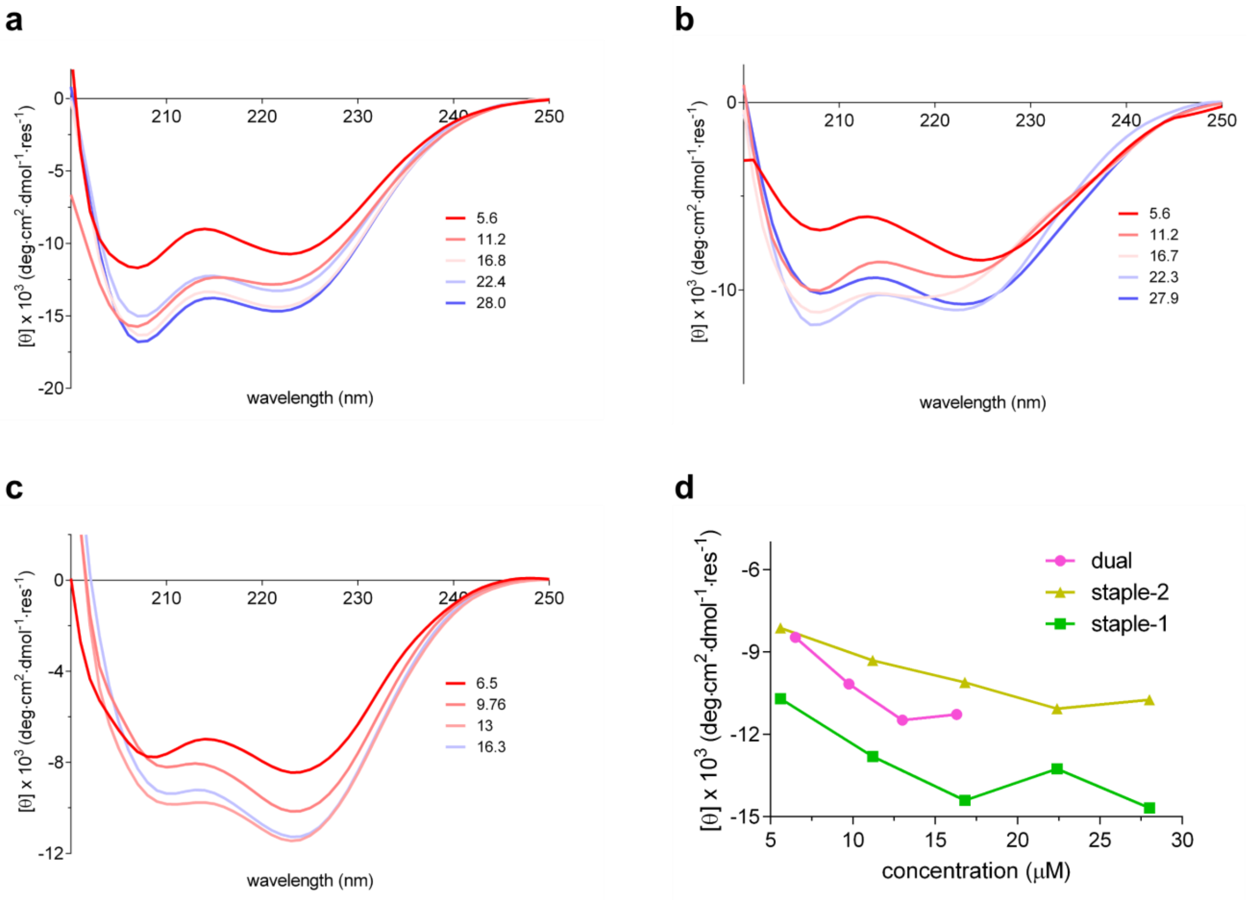

Figure S4 CD curves of **a** dual C-helix, **b** staple-1, and **c** staple-2 at concentrations in the range of 5 to 30 μM in PBS buffer; **d** variations of molar $\theta_{[222]}$ against various concentrations.

The result shows all tested helical peptides have tendency to aggregate when concentrations increase, but they tend to reach more stable states after particular concentration points because their $\theta_{[222]}$ became more constant. For staple-1 that concentration is 16.8 μM, for staple-2 it is 22.3 μM, and for dual it is 13 μM.

CD Spectra of C-helix Dual in Varying Ratios of TFE/PBS Solution

The CD spectra of the dual peptide in varying concentrations suggested that dual formed a more stable multimeric state after 13 μM, so here 16.8 μM was selected. TFE is known to induce the dissociations of oligomers into monomers, and induce helicity of peptides, so a series of solutions of 16.8 μM dual in x% TFE/PBS (x = 10, 20, 30, 40 and 50) were prepared to monitor the dissociation of oligomers.

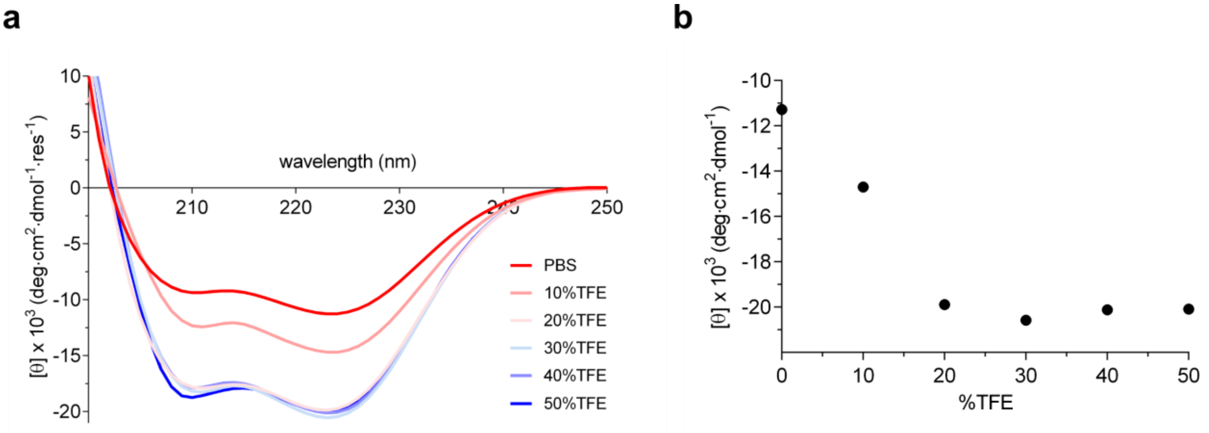

Figure S5 **a** smoothed CD curves of dual C-helix in 0 – 50% TFE/PBS solution; **b** variations of molar ellipticity $\theta_{[223]}$ with increase of %TFE.

The result shows the helicity of dual C-helix gradually increases upon addition of %TFE, and finally reached a plateau when TFE ratios are more than 20%. This plateau indicates maximum helicity and dissociations of oligomers.

CD Spectra of C-helix Peptides at Maximal Helical States in TFE/PBS Buffer

Peptides were induced to reach their maximal helical states in TFE/PBS buffers. Maximal helical states mean no significant improvement of $\theta_{[222]}$ upon increasing the ratios of TFE. The maximal mean-residue molar $\theta_{[222]}$ were used in calculation of experimental percent helicity. The $\theta_{[222]}$ for linear C-helix was obtained from 60% TFE/PBS, and for the others from 50% TFE/PBS.

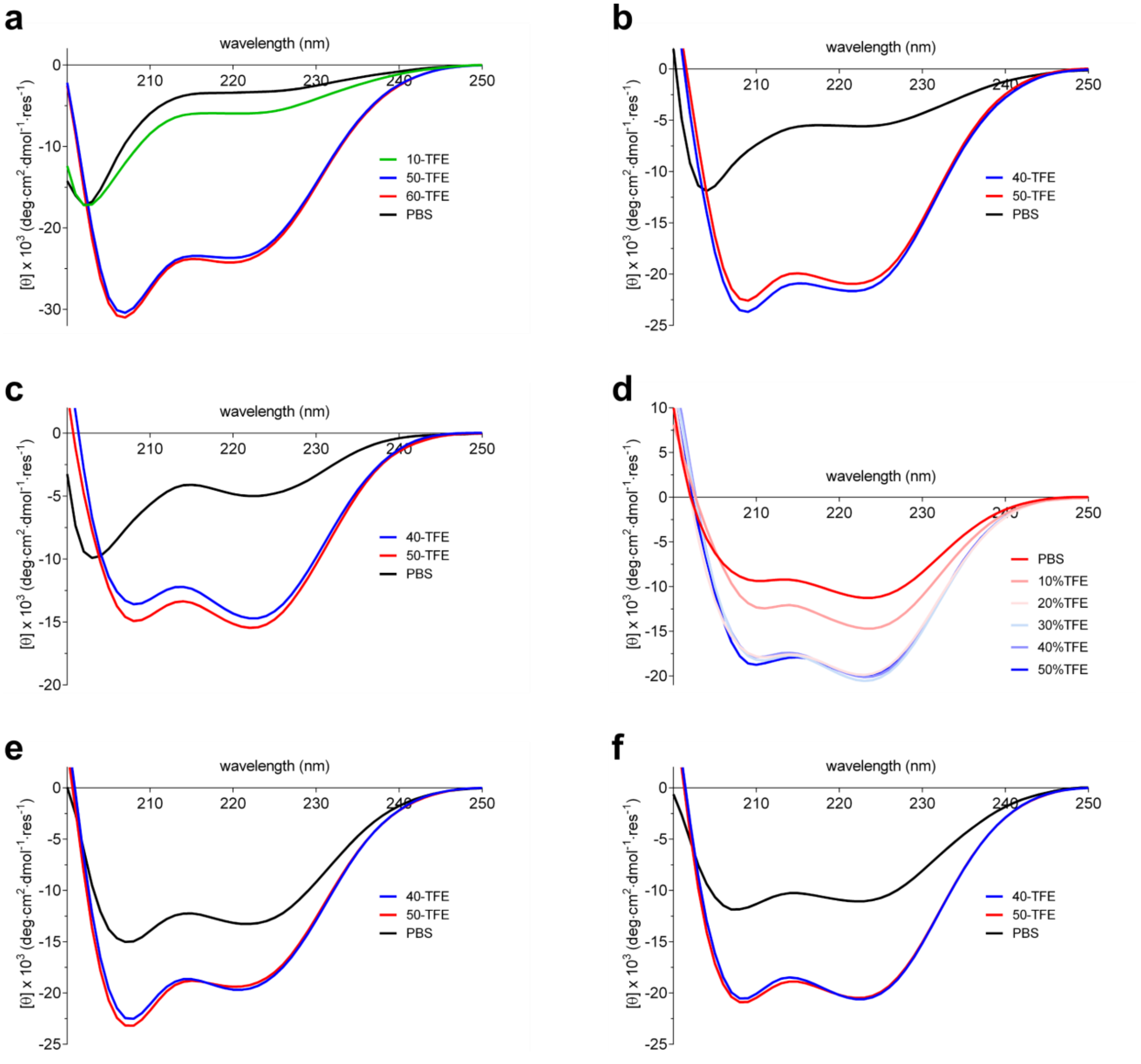

Figure S6 Smoothed CD curves of tested peptides in TFE/PBS solutions. **a** Linear C-helix in PBS, 10%, 50% and 60% TFE/PBS; **b** BAMM C-helix in PBS, 40% and 50% TFE/PBS; **c** BSM C-helix in PBS, 40% and 50 % TFE/PBS; **d** dual C-helix in PBS and 10% - 50 % TFE/PBS; **e** staple-1 in PBS, 40% and 50 % TFE/PBS; **f** staple-2 in PBS, 40% and 50 % TFE/PBS

Calculation of Percent Helicity

Percent helicity of linear peptides were usually calculated by empirical equations: helicity % = $[\theta]_{222}/[\theta]_{max}$, where $[\theta]_{max} = (-44000 + 250T)(1 - k/n)]$ (n = number of amino acids in the peptide, T = 25.0 °C). According to Baldwin's work,[4,5] k refers to 'the number of non-*H*-bonded peptide carbonyls in a carboxyamidated peptide when it is completely helical', hence for linear peptide, k was 3. It has been used for linear and staple peptides, but has *not* been validated for bicyclic capped ones because they have intentionally non-helical residues at both *N*- and *C*-termini to form the caps.

Another way to calculate percent helicity is to enforce peptides to reach maximally helical states by dissolving them in helix-inducing solvent, such as TFE and ACN.[6] In this study, we assume experimentally maximal helicity would arrive for tested peptides when dissolved in high-ratio TFE/PBS mixtures. This has been presented in the previous section and Figure S6. Given the maximal $[\theta]_{222}$ in high-ratio TFE/PBS solutions, experimental % helicity can be calculated: $[\theta]_{222}$ (PBS) / $[\theta]_{222}$ (maximal, in 50% or 60% TFE/PBS).

This manuscript uses the second method to calculate % helicity of all tested peptides because the involvement of non-helical capping residues may alter the theoretical $[\theta]_{max}$ in the first method, and there are no equations to quantify such deviation.

D. Sedimentation Velocity Analytical Ultra-centrifugation (SV-AUC) Experiments on C-helix Dual

C-helix dual peptide was reconstituted in a formulation buffer consisting of 80% PBS and 20% DMSO and subsequently diluted to a solution with Absorbance 0.8 at 280 nm (c = 0.8/1400*1000 = 0.57 mM). SV-AUC measurements were conducted on an Optima Analytical Ultracentrifuge (Beckman Coulter). The rotor used for the measurements was an AN60Ti 4-hole rotor. The measurement cells were Beckmann analytical ultracentrifugation cells with sapphire windows assembled with standard 12 mm pathlength Epon centerpieces. The cell alignment was made with the ZentriForce Pharma optical cell alignment tool. The adequate performance of the instrument regarding its intended application was verified via a system suitability test (SST) prior to sample measurement. The temperature was equilibrated for 1 hour at 20 °C. The time-dependent change of the radial concentration distribution was monitored via absorbance (280 nm) optics at an angular velocity of 60 krpm. Data evaluation was performed with the software Ultrascan.[7] The hydrodynamic parameters (e.g. sedimentation coefficient and molecular weight) were determined through finite element fitting by PCSA-DS-MC (100 Monte Carlo iterations). A partial specific volume of 0.7333 ml/g (standard value for proteins/peptides), a formulation viscosity of 1.48798 cP (simulated from buffer composition within the Ultrascan software) and a formulation density of 1.02404 g/ml (simulated from buffer composition within the Ultrascan software) were assumed for data evaluation.
Measurement results were evaluated by application of fixed integration ranges. The integration ranges are listed in Table S1 and depicted in Figure S7.

Table S1 Integration ranges applied for SV-AUC analysis

Sample	Integration range	
	**Start (S)**	**End (S)**
Species 1	0.150	0.800
Species 2	0.801	1.450
Species 3	1.451	2.700
Species 4	2.710	19.900

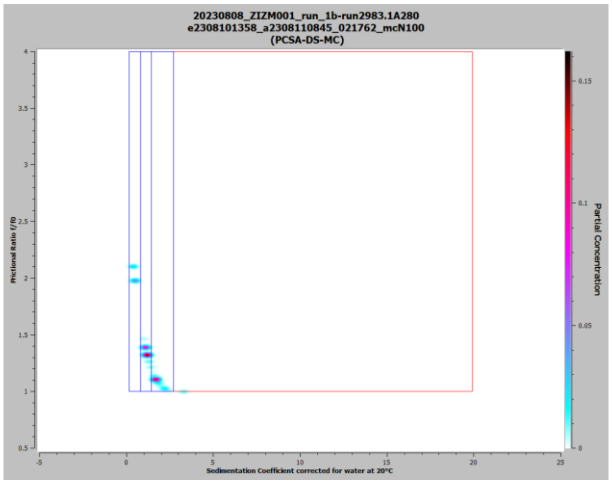

Figure S7 Integration ranges used for SV-AUC data evaluation

SV-AUC measurement results

The results are summarized in Table S2, and graphically depicted in Fig S8.

Four species were found in C-helix dual samples.

Species 1 was characterized by a sedimentation coefficient (s20,w) of 0.46 S, a molecular weight of 4 kDa and a relative content of 11.9%. The molecular weight as well as the sedimentation coefficient indicate that the Species 1 signal corresponds to the C-helix Dual dimer.

Species 2 was characterized by a sedimentation coefficient (s20,w) of 1.17 S, a molecular weight of 9 kDa and a relative content of 45.2%.
The molecular weight as well as the sedimentation coefficient indicate that the Species 2 signal corresponds to the C-helix Dual tetramer.

Species 3 was characterized by a sedimentation coefficient (s20,w) of 1.79 S, a molecular weight of 13 kDa and a relative content of 33.9%.
The molecular weight as well as the sedimentation coefficient indicate that the Species 3 signal corresponds to the C-helix Dual hexamer.

Species 4 was characterized by a sedimentation coefficient (s20,w) of 2.71 S, a molecular weight of 22 kDa and a relative content of 9.0%.
The molecular weight as well as the sedimentation coefficient indicate that the Species 4 signal corresponds to larger oligomers.

Table S2 SV-AUC measurement results of C-helix dual

Sample	Species	Absorbance Optics 280 nm			f/f0 Species 2	RMSD
		s(S)	Mw (kDa)	Amount (%)		
C-helix dual replicate 1	Species 1	0.46	4	12.2	1.34	0.00411
	Species 2	1.17	9	43.4		
	Species 3	1.73	12	32.0		
	Species 4	2.49	19	12.5		
C-helix dual replicate 2	Species 1	0.45	4	11.6	1.35	0.00404
	Species 2	1.16	9	42.7		
	Species 3	1.74	12	35.5		
	Species 4	2.60	20	10.2		
C-helix dual replicate 3	Species 1	0.46	4	11.9	1.33	0.00493
	Species 2	1.19	9	49.6		
	Species 3	1.89	13	34.0		
	Species 4	3.04	25	4.5		
Mean and std	Species 1	0.46 ± 0.01	4 ± 0	11.9 ± 0.3	1.34 ± 0.01	0.00436 ± 0.00050
	Species 2	1.17 ± 0.02	9 ± 0	45.2 ± 3.8		
	Species 3	1.79 ± 0.09	13 ± 1	33.9 ± 1.8		
	Species 4	2.71 ± 0.29	22 ± 3	9.0 ± 4.1		

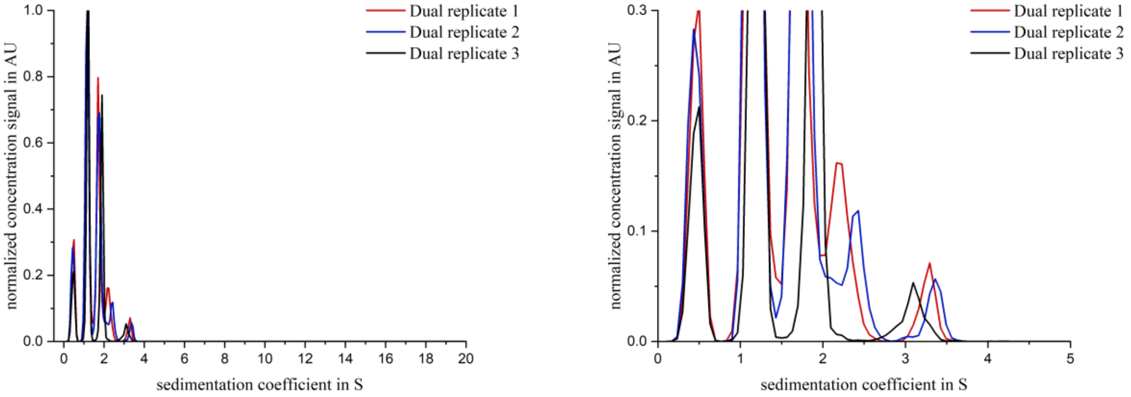

Figure S8 Normalized sedimentation coefficient distribution of C-helix dual as full view (left) and zoom-in (right).

E. Molecular Dynamics (MD) on Modeled Ala-rich 17-mers

Seven Ala-rich helical peptides were prepared virtually for MD simulations. Linear-a, a linear 17-mer, was prepared from an α-helical structure from PDB. Its side chains were manually modified to make its sequence as Ac-CDAACCAAAAACAACGC; here C represents free Cys. BAMM-a, a BAMM *N*-capped 17-mer, was prepared from combination of a linear helical fragment with a NMR-derived BAMM 12-mer (PDB 8UTX). Its sequence is ***C***DAA***CC***AAAAACAACGC; ***C*** represents thioether linked Cys. BSM-a, a BSM *C*-capped 17-mer, was prepared from combination of a linear helical fragment with a NMR-derived BSM 12-mer.[8] Its sequence is Ac-CDAACCAAAAA***C***AA***C***G***C***. Dual-a, a bicyclic dual-capped 17-mer, was a combination of BAMM-a and BSM-a. Its sequence is ***C***DAA***CC***AAAAA***C***AA***C***G***C***. The sequences of these four peptides were kept the same to avoid impacts to helicity from different amino acids.

We also prepared another three 17-mers featuring previously known *N*-caps, *ie* hydrogen bond surrogate (HBS) and amide strap, and a hydrocarbon staple. These peptides necessarily includes unnatural amino acids to form their macrocycles, so the sequences are different from the above four. HBS-a, a HBS *N*-capped 17-mer, was modified from a crystal structure from PDB 4MZL. Its sequence is [XAAA*]$_{cyclo}$AAAAAAAAAAAAAA; X is pent-4-enoic acid and A* is *N*-allyl Ala before metathesis. Strap-a, an amide-strapped *N*-capped 17-mer, was prepared from combination of a linear helical fragment with crystal structure of an amide-strapped *N*-capped peptide (PDB 5GS4). Its sequence is Ac-[^{iso}DAA(Dap)]$_{cyc}$AAAAAAAAAAAAA; ^{iso}D is L-iso-aspartic acid and Dap is (S)-2,3-diaminopropionic acid. Staple-a, a hydrocarbon-stapled 17-mer, was modified from a crystal structure from PDB 4MZK. Its sequence is Ac-AAAAAAA[ZAAAZ]AAAAA; Z is (S)-4'-pentenyl-glycine before metathesis.

All peptides were in helical states (except non-helical capping regions for capped peptides) at the beginning of MD simulations. Below gives their starting conformations.

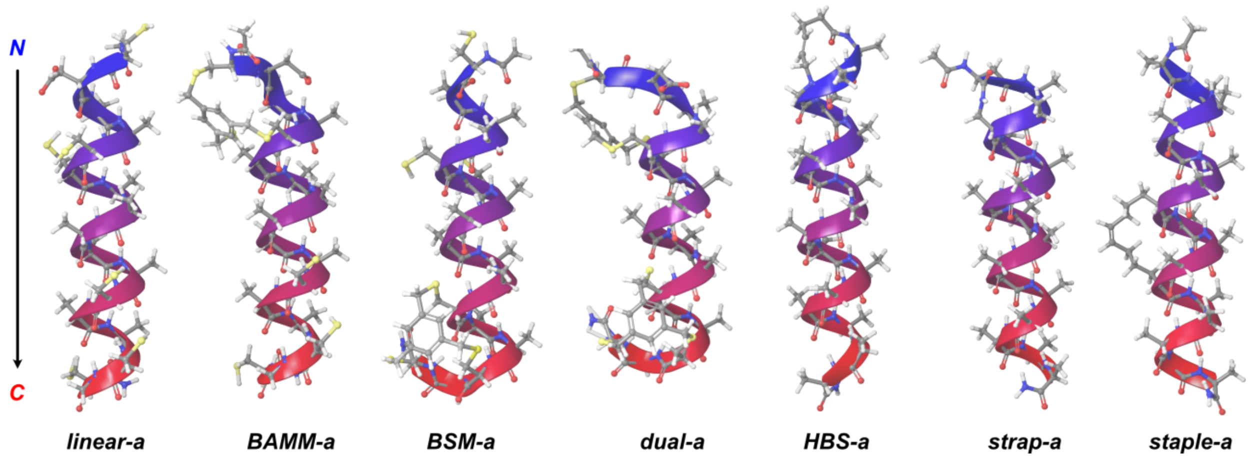

Figure S9 Starting conformations of seven virtual peptides in MD simulations.

The calculation was made in *Desmond*. The peptide was first solvated using *System Builder*, and then put into *Molecular Dynamics* for 200 ns and 2000 frames were kept during the simulation. For each frame, the $C\alpha$ of the 17 residues were overlaid with the input $C\alpha$ atoms giving an RMSD value. The RMSD set of 2000 frames over 200 ns indicated how conformations of the peptide deviated from the starting helical structures during the simulation period.

The main text showed comparison between linear and bicyclic capped 17-mers (Figure 2a). Here gives the comparison among dual-a, HBS-a, strap-a and staple-a. Overall, the bicyclic dual-capped helical mimics most rigidified the helical conformations proved by lowest RMSD deviations (pink line in Figure S10). As expected, mono-*N*-capped peptides were less helix stabilizing (black and red lines). Though their *N*-termini attempted to remain helical, such effect hardly reached their *C*-termini which quickly lost helicity as the simulation started. As for the hydrocarbon-stapled peptide, its less-constrained termini also lost helicity as MD started, but the constrained helical turn in the middle of the peptide managed to induce the termini back to helical conformations later. Such phenomenon periodically occurred for the flexible termini in the stapled peptide (green line), which eventually allows the peptide to remain helical for a significant amount of time, but may also give chance for proteases to trim terminal residues when they temporarily lose helicity.

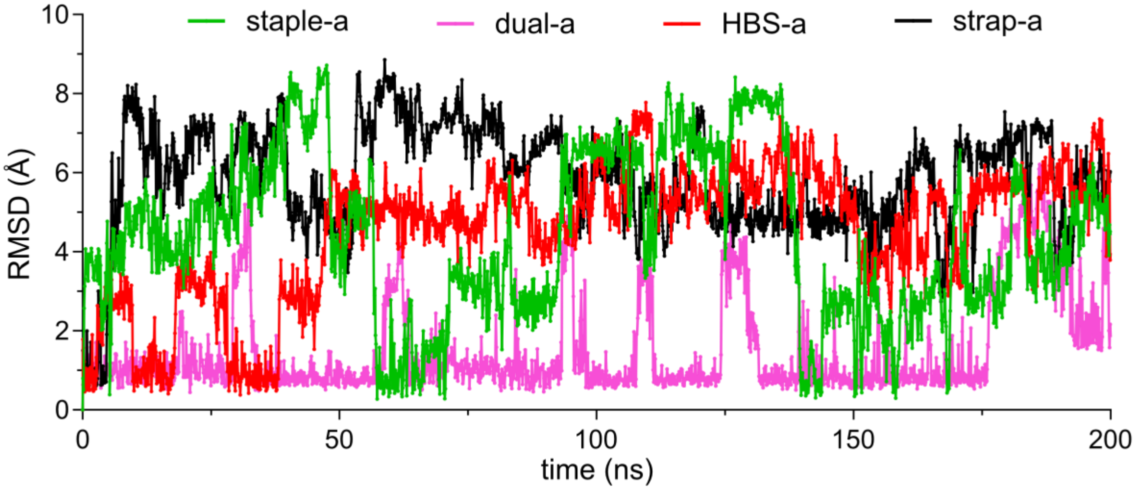

Figure S10 Comparison of bicyclic dual-capped peptide with previously known helix mimics in MD simulations (200 ns).

F. Solvent Accessible Surface Area (SASA) Analysis on C-helix in CDK2 - cyclin E

Hot spot analysis were conducted using solvent accessible surface area (SASA) algorithm.[9,10] The following parameters were calculated for residue 45 – 58 in C-helix that binds cyclin E in PDB 1W98:

ΔSASA(X): The change of solvent accessible surface area for residue X before and after binding the receptor. The parameter can be regarded as the area of X that binds to the receptor. Each residue was assessed for ***hot residue*** by following criteria:

i. *ΔSASA(residue).*
For a hot spot, we required the absolute value to be higher than 50 Å^2.

ii. $\frac{\Delta SASA(residue)}{SASA(residue\ in\ free\ ligand)}$.
For a hot spot, we required the ratio to be higher than 50%, so that the residue is locked inside receptor pocket with less flexibility.

a

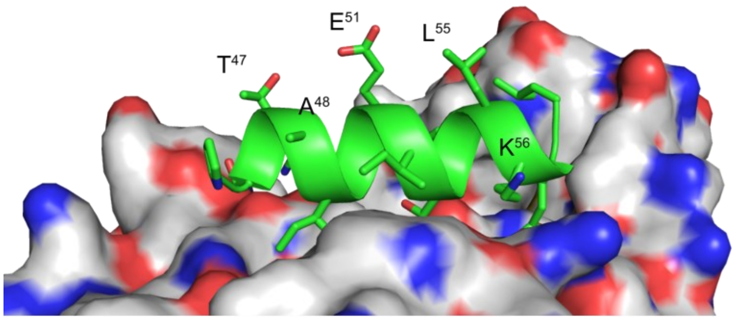

b

ID	amino acid	%buried	ΔSASA (Å^2)
45	P	0.011912856	0.795372009
46	S	0.442728932	33.74054718
47	T	0	0
48	A	0	0
49	I	1	92.86399078
50	R	0.404453225	64.25679016
51	E	0	0
52	I	0.408813088	42.20650482
53	S	0.958784496	60.63493395
54	L	0.118456167	12.79369354
55	L	0	0
56	K	0.328245326	58.39189148
57	E	0.622206958	97.74882126
58	L	0	0

Figure S11 **a** Binding diagram of C-helix (green) with cyclin E (white); Labeled residues are used in mutation to form bicyclic caps or staples. **b** SASA analysis for residue 45 – 58 in C-helix showing % buried and ΔSASA for each residue.

Based on the criteria, Ile49, Ser53 and Glu57 were identified as hot spots. They all fall into the cavity of cyclin E. Following mutations were made for forming caps or staples, or for the functions of peptides, based on crystal structure and SASA result in Figure S11.

(i) Pro45 did not have contacts (< 1 Å^2) with receptor surface, so was mutated to Ala throughout the mimics for improvement of helicity, or formation of BAMM *N*-cap.

(ii) Leu58 did not have contacts with receptor surface, so was mutated to Tyr for quantification of peptide concentrations using absorbance.

(iii) Thr47 and Ala48 did not have contacts with receptor surface, so was mutated to two Cys to form BAMM *N*-cap.

(iv) Lys56 has some contacts with the receptor surface, but mainly by alkyl methylene fragments, so it could be mutated to amino acid with Cβ methylene group. Lys56 was mutated to Cys to form BSM *C*-cap.

(v) Thr47 and Glu51 point to solution and did not have contact or clash with the receptor surface, so they were mutated to (S)-2-(4-pentenyl)Ala for the formation of Staple-1.

(vi) Glu51 and Leu55 point to solution and did not have contact or clash with the receptor surface, so they were mutated to (S)-2-(4-pentenyl)Ala for the formation of Staple-2.

G. NMR Experiments

1D and 2D NMR Spectroscopy

Spectra of 4 mM C-helix dual in TFE-d_3/D_2O/H_2O mixture were recorded on a Bruker AVANCE III 400 at 27 °C. Water suppression was achieved by water gate pulse sequence with gradients for 1D ^{1}H spectrum and by presaturation during relaxation delay for 2D ^{1}H-^{1}H spectra. 2D ^{1}H-^{1}H TOCSY spectrum was acquired with a mixing time of 80 ms, and NOESY spectrum with a mixing time of 200 ms. The spectra were acquired with 12 ppm spectra width and 2048 × 256 complex points. TOCSY spectrum was processed by TopSpin® to 2048 × 2048 complex points, COSY spectrum to 4096 x 4096 complex points, and NOESY spectrum to 2048 × 1024 complex points.

C-helix Dual

Best Solvents for NMR Experiments

4 mM C-helix dual were unable to completely dissolve in H_2O. Gradual titration of TFE-d_3 reveals at least 15% TFE-d_3 were required to help the peptide dissolve. Three solutions of 4 mM C-helix dual in: 1) 20% TFE/H_2O (30 μL D_2O, 120 μL TFE-d_3 and 450 μL H_2O), 2) 30% TFE/H_2O (30 μL D_2O, 180 μL TFE-d_3 and 390 μL H_2O), and 3) 35% TFE/H_2O (30 μL D_2O, 210 μL TFE-d_3 and 360 μL H_2O) were prepared. The fingerprint regions (6.5 – 9.0 ppm) in these 1D H-H spectra were compared.

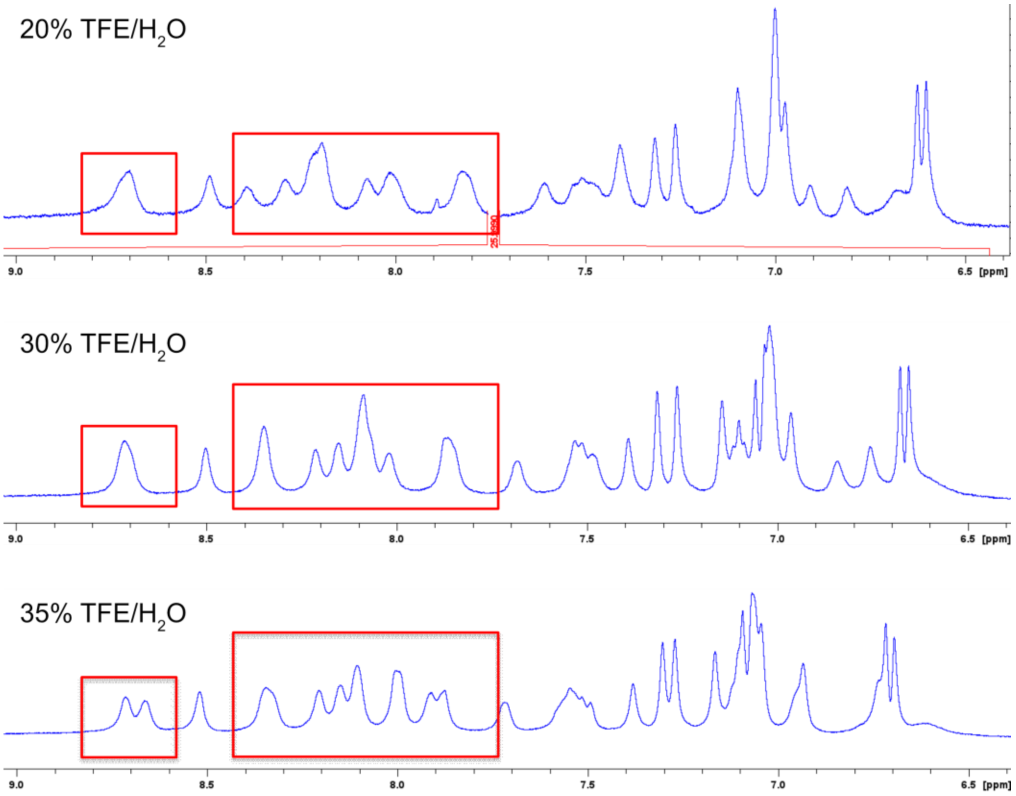

Figure S12 Fingerprint regions (6.5 – 9.0 ppm) of 1D H-H spectra for C-helix dual in aqueous solutions with different ratios of TFE-d_3.

Considering peaks in red boxes of the three spectra, they were more and more separated as the ratios of TFE-d_3 increases, suggesting a gradual dissociation of oligomers. This is consistent with the observation in Figure S5, where 20 μM dual formed a stable composition in solutions with at least 20% TFE. Based on these data, 35% TFE-d_3/H2O was used for further NMR experiments.

NMR Spectra

[C1-D-A-S1-C2-C3]$_{cyclo}$-I1-R-E1-I2-S2-L1-L2-[C4-E2-Y-C5-G-C6]$_{cyclo}$

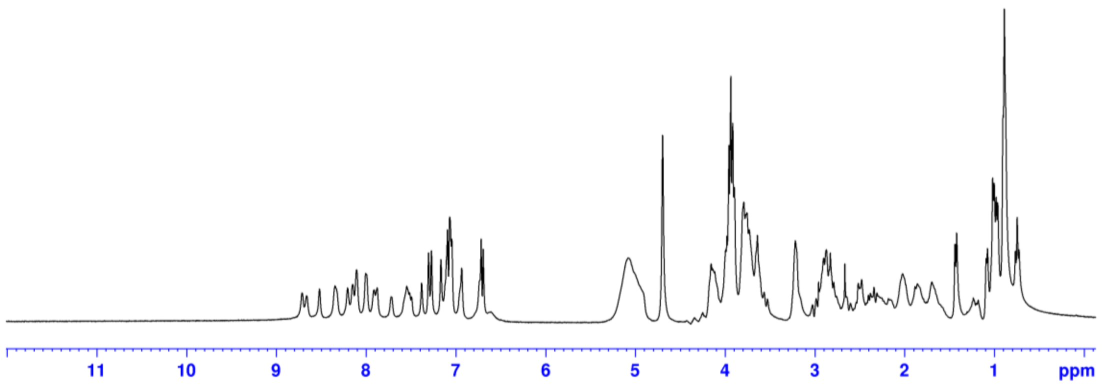

Figure S13 1D H^1 NMR spectrum of C-helix dual.

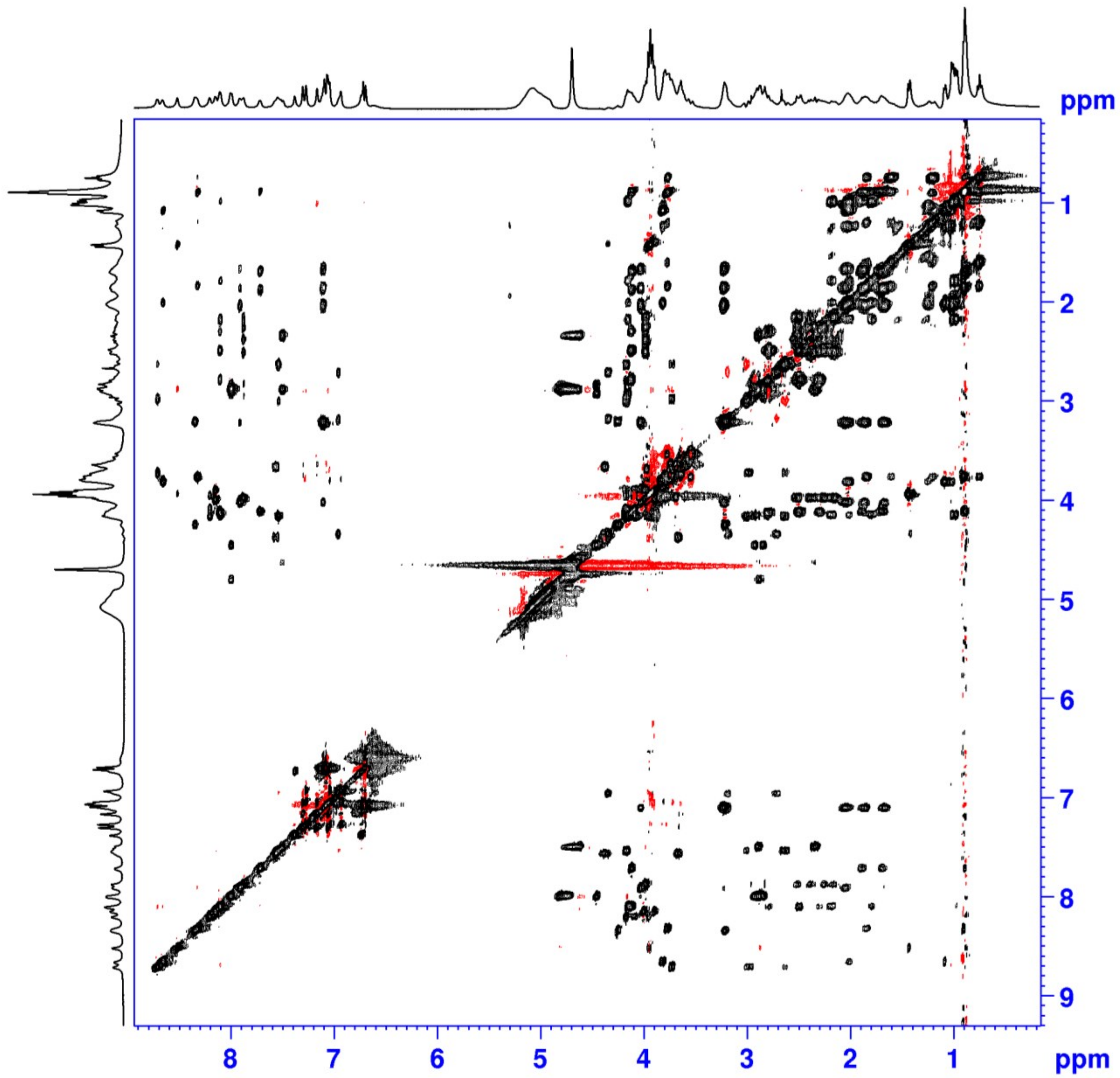

Figure S14 2D H[1]-H[1] TOCSY spectrum of C-helix Dual.

TOCSY fingerprint region

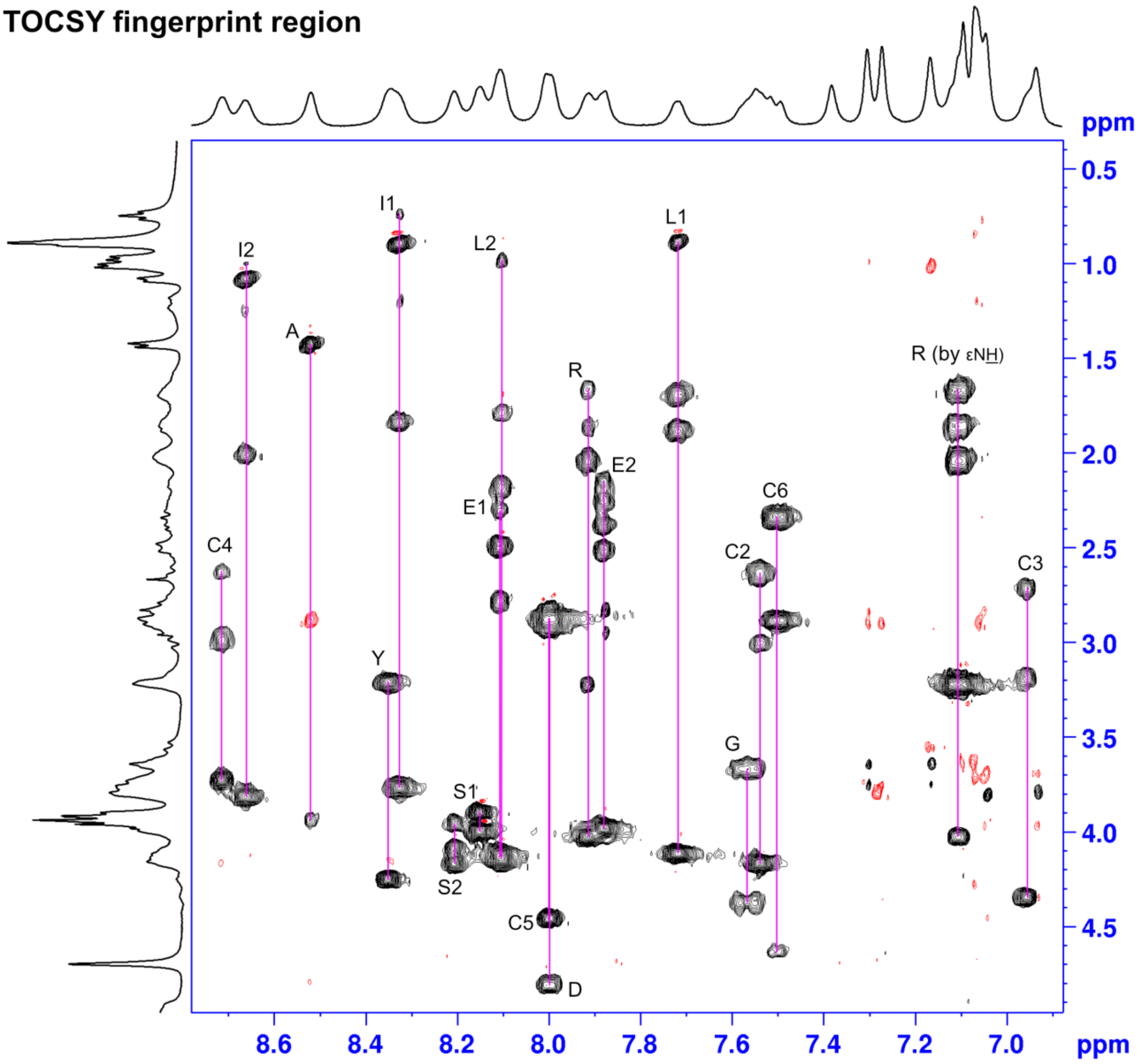

Figure S15 Fingerprint region of TOCSY spectrum

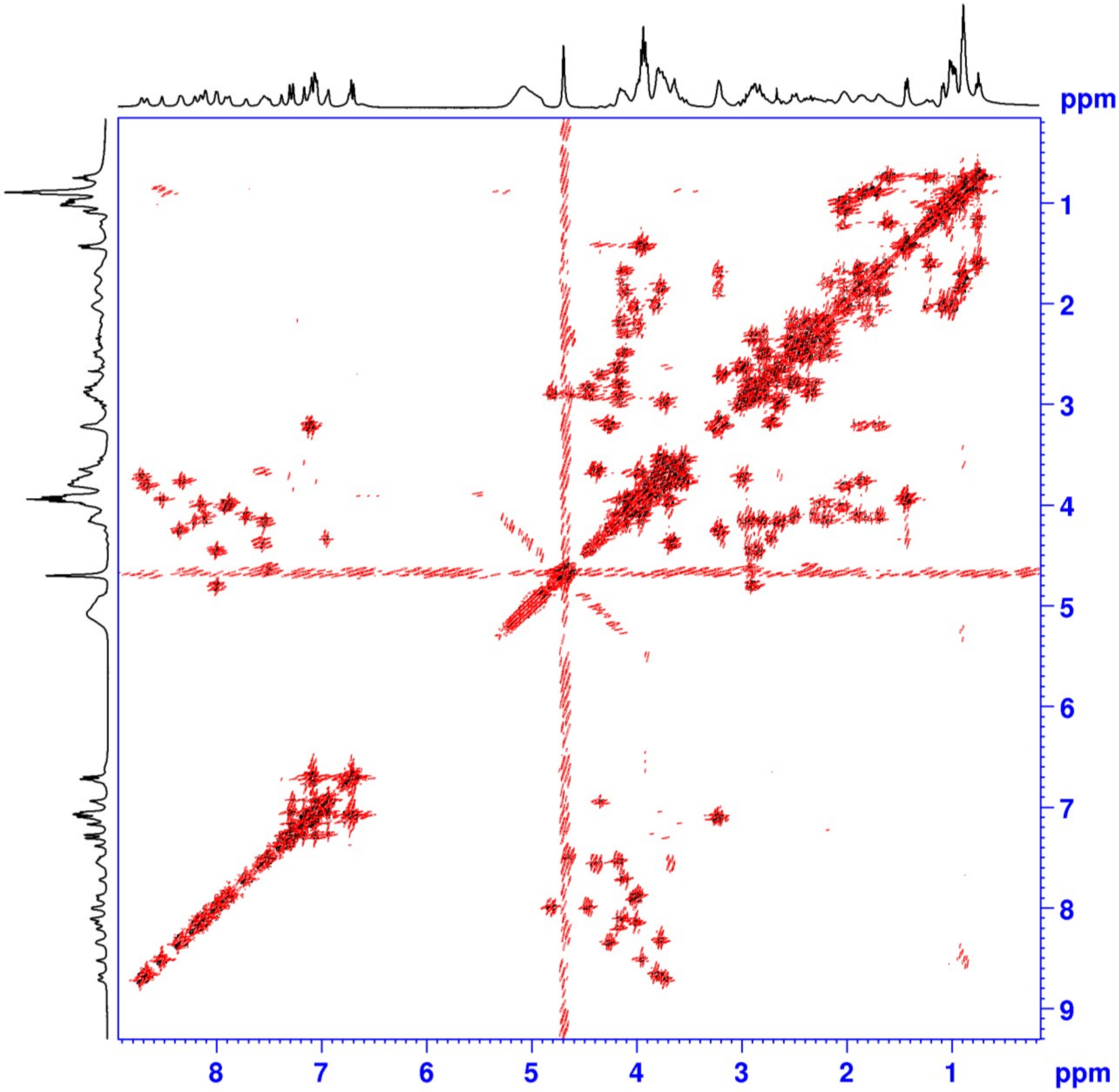

Figure S16 2D H^1-H^1 COSY spectrum of C-helix dual

COSY fingerprint region

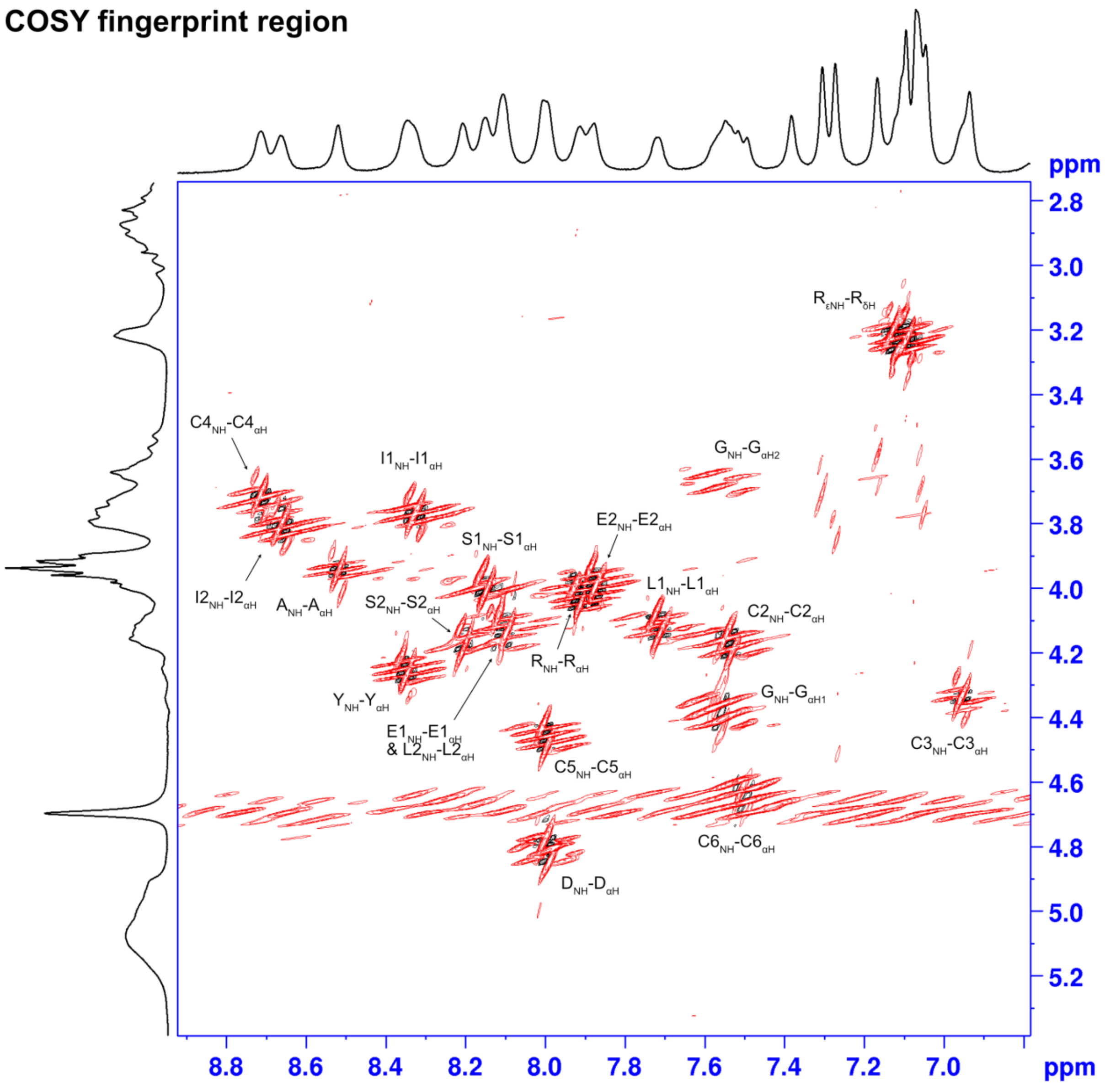

Figure S17 Fingerprint region of COSY spectrum

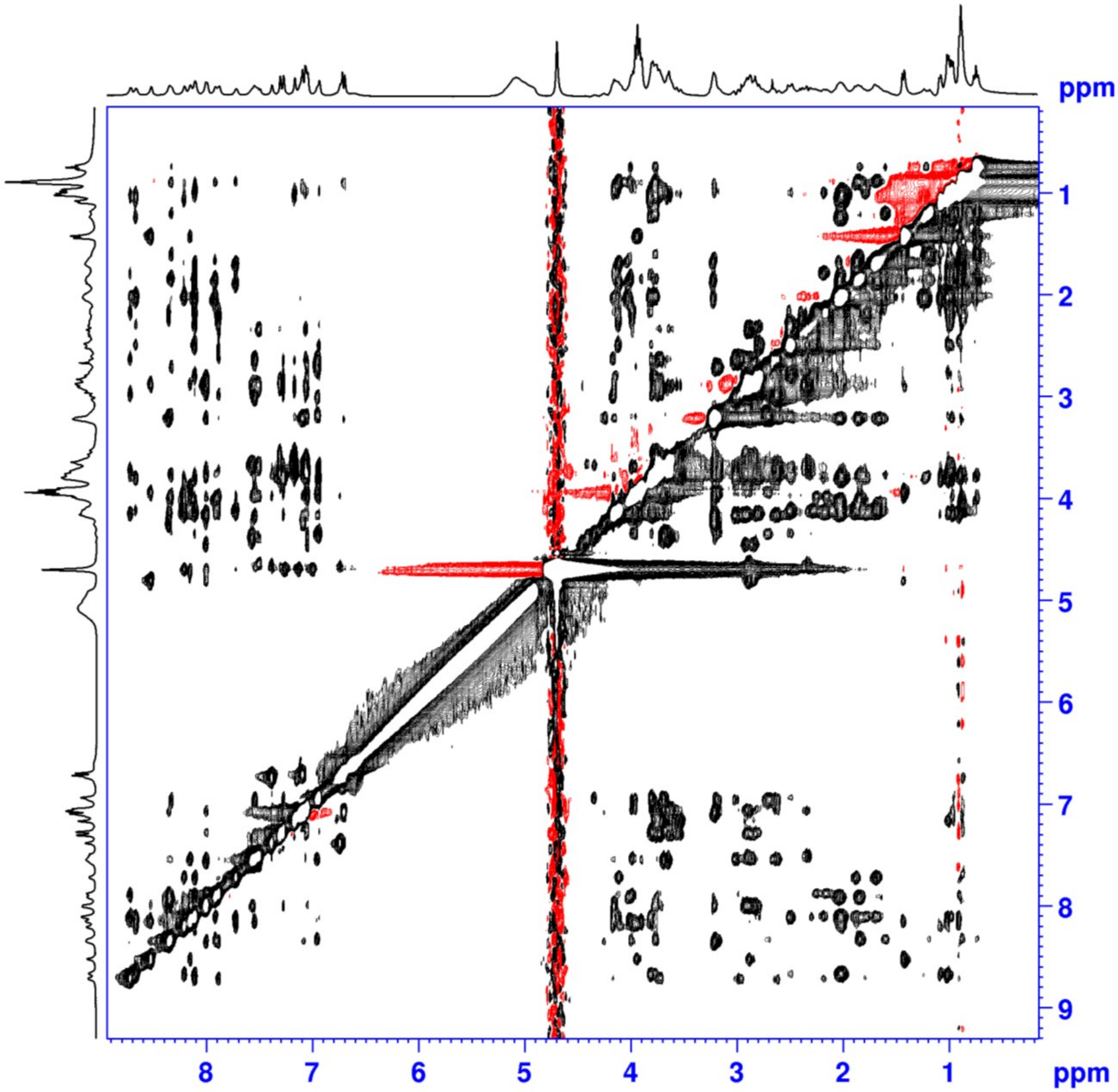

Figure S18 2D H[1]-H[1] NOESY spectrum of C-helix dual

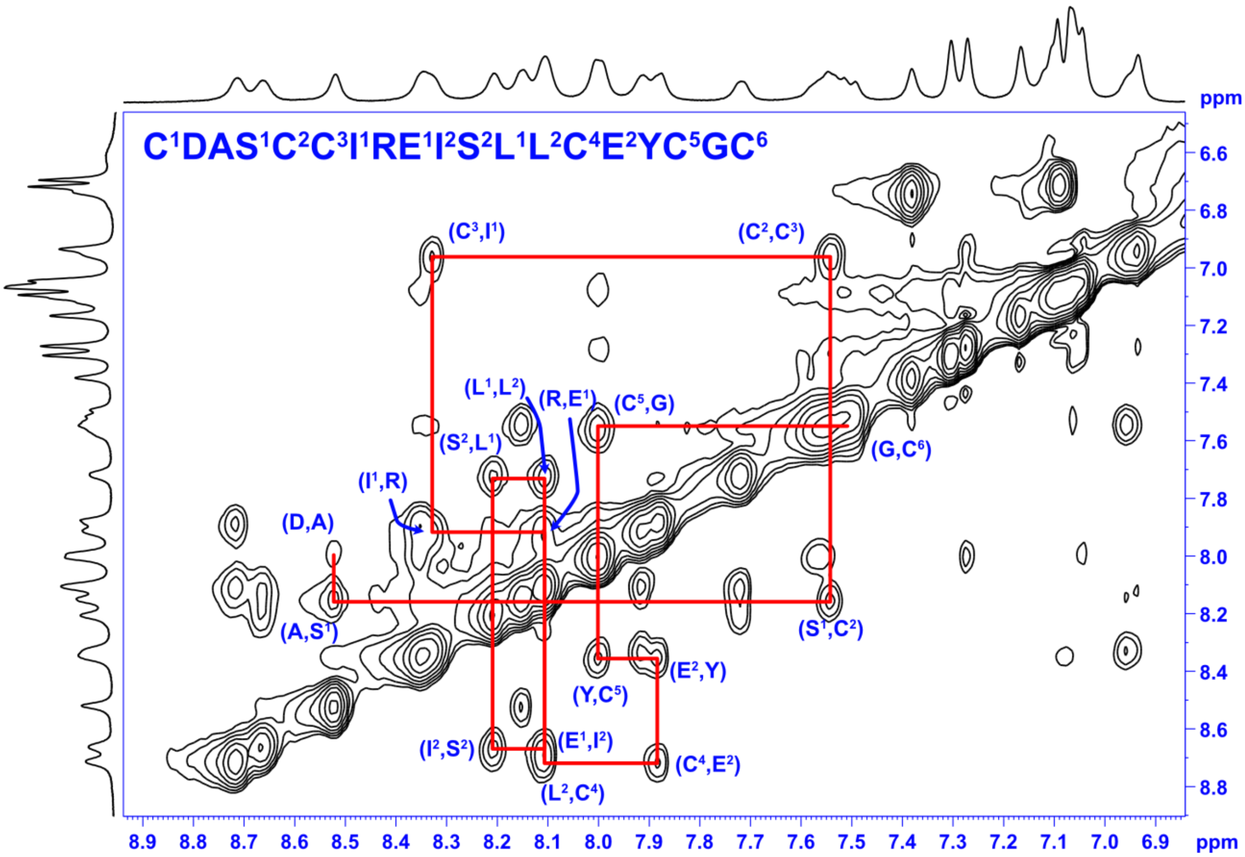

Figure S19 magnified *NH-NH* fingerprints regions of c-helix dual from the NOESY spectrum.

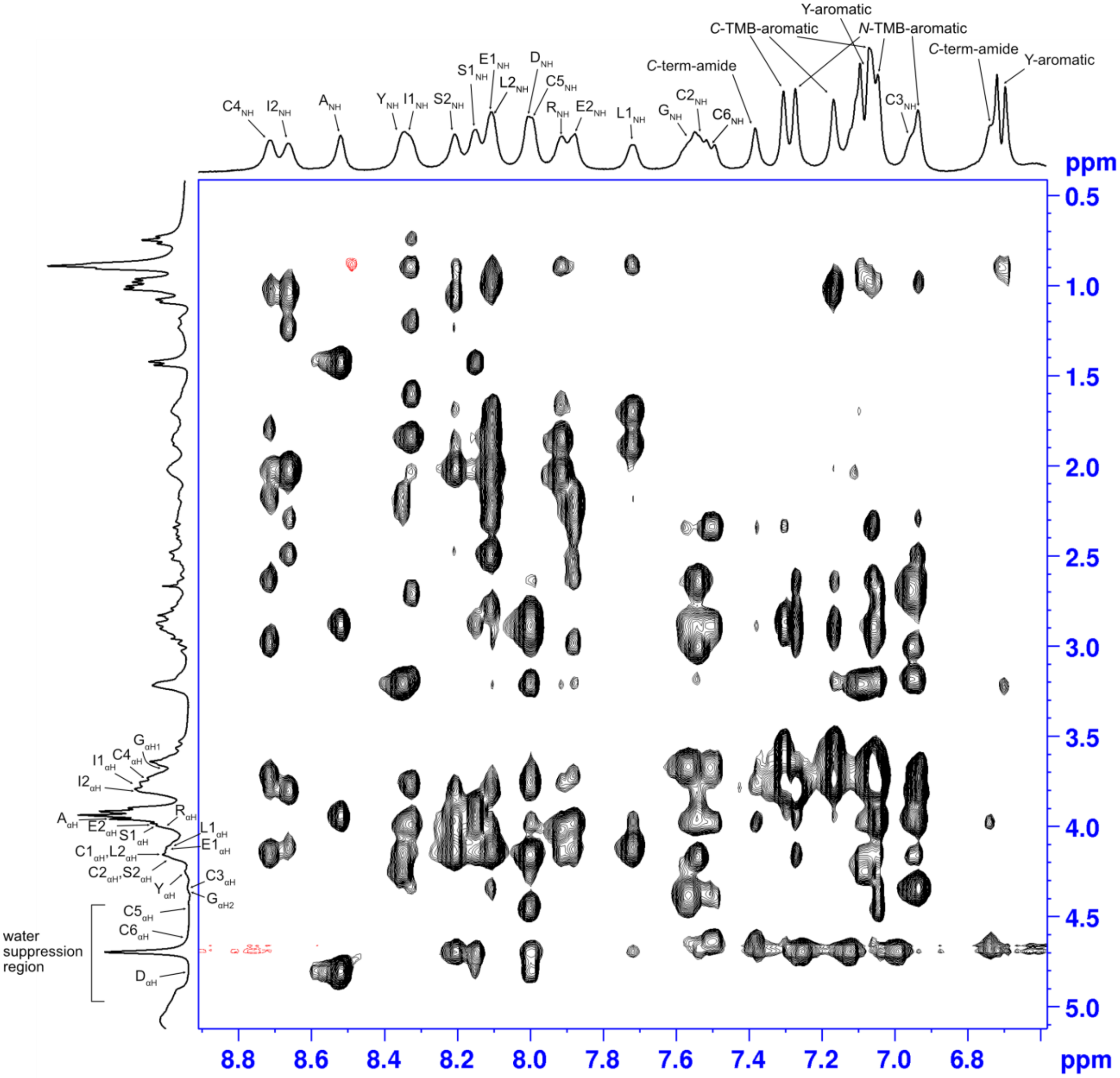

Figure S20 magnified *NH-$C\alpha H$* fingerprints regions of c-helix dual from the NOESY spectrum.

Peak Assignment

[C1-D-A-S1-C2-C3]$_{cyclo}$-I1-R-E1-I2-S2-L1-L2-[C4-E2-Y-C5-G-C6]$_{cyclo}$

Table S3 Peak assignments for all *H* in C-helix dual

Residue	αH	βH	γH	δH	εNH	NH	Aromatic
C1	4.15	2.79, 2.92					
D	4.81	2.88				8.00	
A	3.95	1.43				8.52	
S1	4.00	3.90				8.15	
C2	4.17	2.64, 3.01				7.54	
C3	4.35	2.71, 3.19				6.96	
I1	3.77	1.84	γCH_2 (1.20, 1.60); γCH_3 (0.90)	0.75		8.33	
R	4.03	2.04	1.66, 1.87	3.23	7.11	7.91	
E1	4.13	2.30, 2.50	2.79			8.11	
I2	3.82	2.01	γCH_2 (1.02, 1.26); γCH_3 (1.08)	0.97		8.66	
S2	4.17	3.96, 4.09				8.20	
L1	4.12	1.70, 1.88	1.70	0.89		7.72	
L2	4.15	2.18	1.79	0.99		8.10	
C4	3.73	2.63, 2.98				8.71	
E2	3.98	2.16, 2.25	2.38, 2.51			7.88	
Y	4.25	3.2				8.35	6.71, 7.08
C5	4.46	2.84, 2.93				8.00	
G	3.67, 4.38					7.57	
C6	4.63	2.34, 2.89				7.50	
-NH_2	6.74, 7.38						
N-TMB	C1C2-αH (3.77, 3.79), C2C3-αH (3.72, 3.80), C1C3-αH (3.69, 3.97)						C1C2 (7.27), C2C3 (6.94), C1C3 (7.04)
C-TMB	C4C5-αH (3.64, 3.74), C5C6-αH (3.54, 3.77), C4C6-αH (3.62, 3.74)						C4C5 (7.17), C5C6 (7.30), C4C6 (7.06)

Chemical Shift Index (CSI) Calculation

CSI is a qualitative indicator of peptide/protein secondary structures. By comparing experimental αH chemical shifts with CSI residue-specific random coil αH shifts,[11] the CSI for any peptide can be calculated: $\Delta\delta = \delta_{\alpha H}$ (experimental) - $\delta_{\alpha H}$ (random). Consecutive αH upfield shifts ($\Delta\delta < -0.1$ ppm) is a sign of α-helices; consecutive αH downfield shifts ($\Delta\delta > 0.1$ ppm) indicate β-strand. CSI of dual C-helix indicates a potential α-helical structure since Ala at *N1*.

Distance and Dihedral Constraints

NOEs in the NOESY spectrum were assigned to generate distance restraints as described.[12] Seriously overlapped NOEs were not used in the simulation, but still shown below. A total of 179 distance restraints were then utilized for structure calculation using Macromodel package in *Schrödinger*. An optimized conformational sampling method, Enhanced MCMM,[13] was used to exhaust possible conformations of the input structure and those unmatched conformations were filtered out by the constraints. 31 conformers within 3 kcal/mol (12.6 kJ/mol) to the lowest-energy conformer were then interpreted as the solution structural ensemble.

Table S4 Distance constraints from NOESY spectrum

δ1 [ppm]	δ2 [ppm]	overlapped?	H1 & H2	lower limit	upper limit
**within residue**					
**C1**					
2.92	4.16		C1-βH2 & C1-αH	2.1	3.1
2.80	4.14	overlapped	C1-βH1 & C1-αH; E1-γH & E1-αH	2.0	3.0
**D**					
8.00	4.82		D-NH & D-αH	2.4	3.6
2.88	4.81		D-βH & D-αH	2.0	3.0
**A**					
8.53	3.93		A-NH & A-αH	2.1	3.2
8.54	1.43		A-NH & A-βH	1.9	2.8
1.42	3.95		A-βH & A-αH	1.8	2.7
**S1**					
3.89	8.15		S1-βH & S1-NH	2.2	3.3
4.00	8.16		S1-αH & S1-NH	2.2	3.3
**C2**					
3.01	4.16		C2-βH1 & C2-αH	1.9	2.9
3.00	2.64		C2-βH2 & C2-βH1	1.6	2.4
2.62	4.17	overlapped	C2-βH2 & C2-αH; C4-βH1 & S2-αH	2.1	3.1
7.55	4.17		C2-NH & C2-αH	2.2	3.3
2.64	7.54		C2-βH2 & C2-NH	2.1	3.1
3.00	7.54		C2-βH1 & C2-NH	2.2	3.2
**C3**					
2.70	4.34		C3-βH2 & C3-αH	2.4	3.6

6.95	3.17	overlapped	C3-NH & C3-βH1; N-TMB-C2C3-αroH & C3-βH1	2.1	3.2
6.95	4.35	overlapped	C3-NH & C3-αH; N-TMB-C2C3-αroH & C3-αH	1.8	2.6
3.18	6.95		C3-βH1 & C3-NH	2.2	3.4
2.71	6.95		C3-βH2 & C3-NH	2.0	3.1
3.19	2.71		C3-βH1 & C3-βH2	1.8	2.7
**I1**					
1.19	1.60		I1-γH1 & I1-γH2	1.8	2.6
0.90	3.78		I1-γH(CH3) & I1-αH	2.0	2.9
0.74	3.77		I1-δH & I1-αH	2.3	3.4
0.74	1.83		I1-δH & I1-βH	2.0	3.0
0.74	1.62		I1-δH & I1-γH2	1.9	2.9
0.74	1.20		I1-δH & I1-γH1	1.9	2.9
8.34	3.76		I1-NH & I1-αH	2.2	3.3
8.32	1.85		I1-NH & I1-βH	2.0	3.1
8.33	1.60		I1-NH & I1-γH2	2.3	3.5
8.33	1.20		I1-NH & I1-γH1	2.5	3.7
8.33	0.90		I1-NH & I1-γH(CH3)	2.4	3.6
8.33	0.74		I1-NH & I1-δH	2.9	4.4
**R**					
7.92	4.00	overlapped	R-NH & R-αH; R-NH & S1-αH	2.1	3.2
7.92	3.22		R-NH & R-δH	3.1	4.7
2.02	7.91		R-βH & R-NH	1.9	2.8
1.83	7.92	overlapped	R-γH2 & R-NH; I1-βH & R-NH	2.0	2.9
7.92	1.65		R-NH & R-γH1	2.6	3.9
3.23	2.05		R-δH & R-βH	2.2	3.3
3.23	1.86		R-δH & R-γH2	2.1	3.2
3.22	1.67		R-δH & R-γH1	2.1	3.1
2.04	4.00		R-βH & R-αH	2.1	3.1
**E1**					
8.10	4.11	overlapped	E1-NH & E1-αH; E1-NH & R-αH	1.8	2.7
8.11	2.78		E1-NH & E1-γH	2.3	3.5
2.50	8.09		E1-βH2 & E1-NH	2.2	3.3
2.29	8.10		E1-βH1 & E1-NH	2.6	4.0
2.49	2.79		E1-βH2 & E1-γH	1.6	2.5
2.49	4.14		E1-βH2 & E1-αH	1.9	2.8
2.29	4.11		E1-βH1 & E1-αH	2.0	2.9
**I2**					
2.01	3.79	overlapped	I2-βH & I2-αH; I2-βH & I1-αH	1.8	2.7
1.08	3.79		I2-γH(CH3) & I2-αH	2.0	3.0
1.08	2.02		I2-γH(CH3) & I2-βH	1.8	2.7
8.66	3.80	overlapped	I2-NH & I2-αH; I2-NH & I1-αH	2.2	3.3
8.67	1.23		I2-NH & I2-γH2	2.4	3.7
0.96	8.68		I2-δH & I2-NH	2.7	4.1
1.01	8.67		I2-γH1 & I2-NH	2.2	3.2
1.08	8.67		I2-γH(CH3) & I2-NH	2.3	3.5

2.01	8.67		I2-βH & I2-NH	1.8	2.7
2.03	1.24		I2-βH & I2-γH2	1.9	2.8
**S2**					
8.21	4.13	overlapped	S2-NH & S2-βH2; S2-NH & S2-αH	1.8	2.7
8.21	3.96		S2-NH & S2-βH1	2.1	3.1
**L1**					
1.69	0.89		L1-γH & L1-δH	2.1	3.1
1.69	4.12	overlapped	L1-βH1 & L1-αH; L1-γH & L1-αH	1.8	2.7
1.88	4.13	overlapped	L1-βH2 & L1-αH; L1-βH2 & E1-αH	2.0	3.0
0.88	4.12		L1-δH & L1-αH	1.8	2.6
7.73	4.13	overlapped	L1-NH & L1-αH; L1-NH & S2-αH	1.9	2.8
7.73	1.89		L1-NH & L1-βH2	2.1	3.1
7.73	0.87		L1-NH & L1-δH	2.6	3.9
**L2**					
2.17	4.15	overlapped	L2-βH & L2-αH; E2-βH & L1-αH	2.1	3.1
0.99	2.20		L2-δH & L2-βH	1.9	2.9
0.99	1.79		L2-δH & L2-γH	1.9	2.8
2.18	8.09		L2-βH & L2-NH	2.3	3.4
1.79	8.10		L2-γH & L2-NH	2.2	3.3
1.00	8.10		L2-δH & L2-NH	2.4	3.5
**C4**					
8.72	3.75		C4-NH & C4-αH	2.2	3.3
8.71	2.96		C4-NH & C4-βH2	2.2	3.4
8.72	2.63		C4-NH & C4-βH1	2.4	3.6
**E2**					
7.88	3.97		E2-NH & E2-αH	2.2	3.2
7.88	2.52		E2-NH & E2-γH2	2.8	4.2
2.38	7.86		E2-γH1 & E2-NH	2.9	4.3
2.15	7.88		E2-βH2 & E2-NH	2.3	3.5
2.24	7.88		E2-βH1 & E2-NH	2.3	3.5
2.25	3.97		E2-βH1 & E2-αH	2.6	3.9
2.15	3.98		E2-βH2 & E2-αH	2.3	3.5
**Y**					
4.25	8.35		Y-αH & Y-NH	2.7	4.0
8.35	3.21		Y-NH & Y-βH	1.8	2.7
7.08	8.33		Y-aroH1 & Y-NH	2.7	4.0
6.70	7.08		Y-aroH2 & Y-aroH1	1.6	2.5
3.21	7.07		Y-βH & Y-aroH1	2.0	3.0
7.09	4.24		Y-aroH1 & Y-αH	2.2	3.2
6.70	4.25		Y-aroH2 & Y-αH	3.9	5.8
6.70	3.23		Y-aroH2 & Y-βH	2.8	4.2
**C5**					
8.01	4.45		C5-NH & C5-αH	2.2	3.2
2.92	4.44		C5-βH2 & C5-αH	2.2	3.3
2.84	4.45		C5-βH1 & C5-αH	2.1	3.2

**G**					
7.57	4.38		G-NH & G-αH2	1.9	2.9
7.58	3.66		G-NH & G-αH1	1.9	2.9
**C6**					
2.88	2.33		C6-βH2 & C6-βH1	1.7	2.6
7.49	4.62		C6-NH & C6-αH	2.3	3.5
7.50	2.89		C6-NH & C6-βH2	2.1	3.2
7.50	2.34		C6-NH & C6-βH1	2.1	3.2
**C-terminal amide**					
6.75	7.39		amide-NH1 & amide-NH2	1.6	2.4
**cross residues**					
N(i)-N(i+1)					
8.00	8.53		D-NH & A-NH	3.4	5.1
8.16	8.52		S1-NH & A-NH	2.5	3.7
7.54	8.15		C2-NH & S1-NH	2.3	3.5
6.95	7.54		C3-NH & C2-NH	2.2	3.3
6.95	8.32		C3-NH & I1-NH	2.3	3.5
7.92	8.33		R-NH & I1-NH	2.4	3.5
7.91	8.11		R-NH & E1-NH	2.3	3.4
8.66	8.10		I2-NH & E1-NH	2.4	3.5
8.21	8.66		S2-NH & I2-NH	2.3	3.4
8.21	7.72		S2-NH & L1-NH	2.3	3.5
8.11	7.72		L2-NH & L1-NH	2.2	3.4
8.72	8.10		C4-NH & L2-NH	2.2	3.3
8.71	7.89		C4-NH & E2-NH	2.3	3.4
7.88	8.34		E2-NH & Y-NH	2.3	3.5
8.00	8.34		C5-NH & Y-NH	2.3	3.4
7.57	7.99		G-NH & C5-NH	2.4	3.6
N(i)-N(i+2)					
8.53	7.54		A-NH & C2-NH	3.3	5.0
α(i)-N(i+1)					
8.01	4.15		C1-AH & D-NH	1.9	2.8
8.53	4.79		A-NH & D-AH	1.8	2.7
3.95	8.14		A-αH & S1-NH	2.6	3.8
4.00	7.54		S-αH & C2-NH	2.4	3.6
6.96	4.17		C3-NH & C2-αH	2.8	4.2
8.33	4.37		I1-NH & C3-αH	2.8	4.2
7.92	3.75		R-NH & I1-αH	2.8	4.2
8.10	4.11	overlapped	E1-NH & R-αH; E1-NH & E1-αH; L2-NH & L2-αH; L2-NH & L1-αH; E1-NH & C2-αH; L2-NH & E1-αH	1.8	2.7
8.66	4.11		I2-NH & E1-αH	2.6	3.9
8.21	3.79	overlapped	S2-NH & I2-αH; S2-NH & I1-αH	2.5	3.7
7.73	4.13	overlapped	L1NH & S2-αH; L1-NH & L1-αH; L1NH & E1αH	1.9	2.8
8.10	4.11	overlapped	L2NH & L1αH; E1-NH & R-αH; E1-NH & E1-αH;	1.8	2.7

			L2NH & L2αH; E1NH & C2αH; L2-NH & E1-αH		
8.71	4.16	overlapped	C4-NH & L2-αH; C4-NH & S2-αH	2.3	3.4
7.88	3.72		E2-NH & C4-αH	2.7	4.0
8.34	3.97	overlapped	Y-NH & E2-αH; I1-NH & A-αH	2.1	3.1
8.00	4.27		C5-NH & Y-αH	2.4	3.6
7.50	4.35		C6-NH & G-αH2	2.5	3.8
7.50	3.67		C6-NH & G-αH1	2.1	3.2
α(i)-N(i+2)					
7.99	3.97		C5-NH & E2-αH	2.7	4.1
α(i)-N(i+3)					
7.92	4.16		R-NH & C2-αH	2.5	3.8
8.11	4.35		E1-NH & C3-αH	2.7	4.1
8.66	3.80	overlapped	I2-NH & I1-αH; I2-NH & I2-αH	2.2	3.3
8.21	4.13	overlapped	S2-NH & R-αH; S2-NH & S2-βH2; S2-NH & S2-αH	1.8	2.7
7.73	4.13	overlapped	L1-NH & E1-αH; L1-NH & L1-αH; L1-NH & S2-αH	1.9	2.8
8.11	3.80		L2-NH & I2-αH	2.4	3.7
8.71	4.16	overlapped	C4-NH & S2-αH; C4-NH & L2-αH	2.3	3.4
7.88	4.13	overlapped	E2-NH & L1-αH; E2-NH & S2-αH	2.3	3.4
4.15	8.36	overlapped	L2-αH & Y-NH; L1-αH & Y-NH	3.0	4.4
3.76	7.99	overlapped	C4-αH & C5-NH; N-TMB-C1-αH1 & D-NH	2.3	3.4
α(i)-N(i+4)					
8.34	3.97	overlapped	I1-NH & A-αH; Y-NH & E2-αH	2.1	3.1
7.92	4.00	overlapped	R-NH & S1-αH; R-NH & R-αH	2.1	3.2
8.10	4.11	overlapped	E1NH & C2αH; L2NH & L1αH; E1-NH & R-αH; E1-NH & E1-αH; L2NH & L2αH; L2-NH & E1-αH	1.8	2.7
8.66	4.35		I2-NH & C3-αH	3.3	5.0
8.21	3.79	overlapped	S2-NH & I1-αH; S2-NH & I2-αH	2.5	3.7
8.10	4.11	overlapped	L2-NH & E1-αH; E1NH & C2αH; L2NH & L1αH; E1-NH & R-αH; E1-NH & E1-αH; L2NH & L2αH	1.8	2.7
7.88	4.13	overlapped	E2-NH & S2-αH; E2-NH & L1-αH	2.3	3.4
4.15	8.36	overlapped	L2-αH & Y-NH; L1-αH & Y-NH	3.0	4.4
7.50	3.99		C6-NH & E2-αH	2.4	3.6
α(i)-N(i+5)					
6.74	3.97		AMIDE-NH2 & E2-αH	2.9	4.4
7.38	3.97		AMIDE-NH1 & E2-αH	2.8	4.2
α(i)-β(i+3)					
2.70	3.95		C3-βH2 & A-αH	2.4	3.6
2.02	4.16		R-βH & C2-αH	2.0	2.9
2.29	4.34		E1-βH1 & C3-αH	2.9	4.4
2.49	4.34		E1-βH2 & C3-αH	2.5	3.8
2.01	3.79	overlapped	I2-βH & I1-αH; I2-βH & I2-αH;	1.8	2.7
1.88	4.13	overlapped	L1-βH2 & E1-αH; L1-βH2 & L1-αH;	2.0	3.0
1.69	4.12	overlapped	L1-βH1 & E1-αH; L1-βH1 & L1-αH; L1-γH & L1-αH	1.8	2.7
2.18	3.82		L2-βH & I2-αH	2.8	4.3

2.62	4.17	overlapped	C4-βH1 & S2-αH; C2-βH2 & C2-αH	2.1	3.1
2.17	4.15	overlapped	E2-βH & L1-αH; L2-βH & L2-αH	2.1	3.1
4.16	3.21		L2-αH & Y-βH	2.8	4.2
β(i)-N(i+1)					
2.78	8.01		C1-βH1 & D-NH	2.6	4.0
8.53	2.87		A-NH & D-βH	2.1	3.2
8.15	1.42		S1-NH & A-βH	2.3	3.5
3.89	7.54		S1-βH & C2-NH	2.6	4.0
2.62	6.96		C2-βH2 & C3-NH	2.1	3.2
6.95	3.01		C3-NH & C2-βH1	2.3	3.4
8.33	2.72		I1-NH & C3-βH2	2.4	3.6
1.83	7.92	overlapped	I1-βH & R-NH; R-γH2 & R-NH	2.0	2.9
2.03	8.10		R-βH & E1-NH	1.9	2.9
8.67	2.30		I2-NH & E1-βH1	2.5	3.8
8.66	2.49		I2-NH & E1-βH2	2.5	3.7
8.21	2.01		S2-NH & I2-βH	2.0	3.1
7.72	3.96		L1-NH & S2-βH1	3.0	4.4
1.89	8.10		L2-NH & L1-βH2	2.2	3.3
1.69	8.10		L1-βH1 & L2-NH	2.2	3.3
8.71	2.18		C4-NH & L2-βH	2.4	3.6
7.88	2.62		E2-NH & C4-βH1	2.7	4.1
7.88	2.97		E2-NH & C4-βH2	2.4	3.6
2.24	8.36		E2-βH1 & Y-NH	3.2	4.8
2.16	8.36		E2-βH2 & Y-NH	2.7	4.0
8.00	3.21		C5-NH & Y-βH	2.3	3.4
7.39	2.87		AMIDE-NH1 & C6-βH2	3.0	4.5
7.38	2.34		AMIDE-NH1 & C6-βH1	3.0	4.5
**cap-related**					
N-cap					
2.49	3.79		E1-βH2 & N-TMB-C2αH2	2.3	3.4
3.21	3.72		C3-βH1 & N-TMB-C3αH1	2.5	3.7
3.20	3.96		C3-βH1 & N-TMB-C3αH2	2.5	3.7
7.05	7.99		N-TMB-C1C3aro & D-NH	2.8	4.3
8.00	7.27		D-NH & N-TMB-C1C2aro	2.6	4.0
7.05	3.20		N-TMB-C1C3aro & C3-βH1	2.7	4.0
7.05	3.96		N-TMB-C1C3aro & N-TMB-C3-αH2	1.9	2.9
7.27	3.79	overlapped	N-TMB-C1C2aro & N-TMB-C1αH; N-TMB-C1C2aro & N-TMB-C2αH2	1.6	2.4
7.27	2.87		N-TMB-C1C2aro & N-TMB-C1αH; N-TMB-C1C2aro & N-TMB-C2αH2	2.1	3.2
7.27	4.16		N-TMB-C1C2aro & C1-αH	2.7	4.0
7.27	2.63		N-TMB-C1C2aro & C2-βH2	2.7	4.1
6.94	3.82		N-TMB-C2C3aro & N-TMB-C2αH2	1.9	2.9
6.94	3.69	overlapped	N-TMB-C2C3aro & N-TMB-C3αH1; N-TMB-C2C3aro & N-TMB-C2αH1	2.0	3.0
6.94	2.49		N-TMB-C2C3aro & E1-βH2	2.7	4.1

6.94	2.30		N-TMB-C2C3aro & E1-βH1	3.0	4.6
6.95	3.97	overlapped	C3-NH & N-TMB-C3-αH2; N-TMB-C2C3-aroH & N-TMB-C3-αH2	1.9	2.9
6.95	3.17	overlapped	N-TMB-C2C3-aroH & C3-βH1; C3-NH & C3-βH1	2.1	3.2
6.95	4.35	overlapped	N-TMB-C2C3-aroH & C3-αH; C3-NH & C3-αH	1.8	2.6
3.76	7.99	overlapped	N-TMB-C1-αH1 & D-NH; C4-αH & C5-NH	2.3	3.4
C-cap					
3.55	2.88		C-TMB-C5αH1 & C5-βH	2.3	3.5
3.55	1.01		C-TMB-C5αH1 & L2-DH	2.4	3.6
2.32	3.68		C6-βH1 & C-TMB-C6αH2	1.8	2.8
7.17	8.70		C-TMB-C4C5aro & C4-NH	3.2	4.8
3.75	7.06	overlapped	C-TMB-C6-αH1 & C-TMB-C4C6aro; C-TMB-C4-αH1 & C-TMB-C4C6aro	1.7	2.5
3.64	7.06	overlapped	C-TMB-C6-αH2 & C-TMB-C4C6aro; C-TMB-C4-αH2 & C-TMB-C4C6aro	1.8	2.7
7.06	2.90		C-TMB-C4C6aro & C6-βH2	2.0	3.0
7.06	2.63		C-TMB-C4C6aro & C4-βH1	2.6	3.8
7.06	2.32		C-TMB-C4C6aro & C6-βH1	2.3	3.5
7.32	3.79	overlapped	C-TMB-C5C6aro & C-TMB-C6αH2; C-TMB-C5C6aro & C-TMB-C5αH2	1.7	2.6
3.62	7.30		C-TMB-C6αH1 & C-TMB-C5C6aro	2.1	3.2
3.55	7.30		C-TMB-C5αH1 & C-TMB-C5C6aro	2.3	3.5
7.31	2.87	overlapped	C-TMB-C5C6aro & C6-βH2; C-TMB-C5C6aro & C5-βH	2.1	3.1
7.31	2.34		C-TMB-C5C6aro & C6-βH1	3.0	4.5
3.73	7.16		C-TMB-C4αH2 & C-TMB-C4C5aro	2.0	2.9
3.64	7.16		C-TMB-C4αH1 & C-TMB-C4Caro	2.2	3.3
3.54	7.16		C-TMB-C5αH1 & C-TMB-C4C5aro	2.0	3.0
7.17	2.90		C-TMB-C4C5aro & C5-βH	2.2	3.4
7.17	2.64		C-TMB-C4C5aro & C4-βH1	2.8	4.2
others					
2.49	1.01		E1-βH2 & L2-δH	2.6	4.0
1.42	2.89		A-βH & D-βH	2.5	3.7
7.57	2.36		G-NH & C6-βH1	2.8	4.1
8.72	1.80		C4-NH & L2-γH	2.6	3.9
8.71	1.02		C4-NH & L2-δH	2.6	3.9

NOE Summary

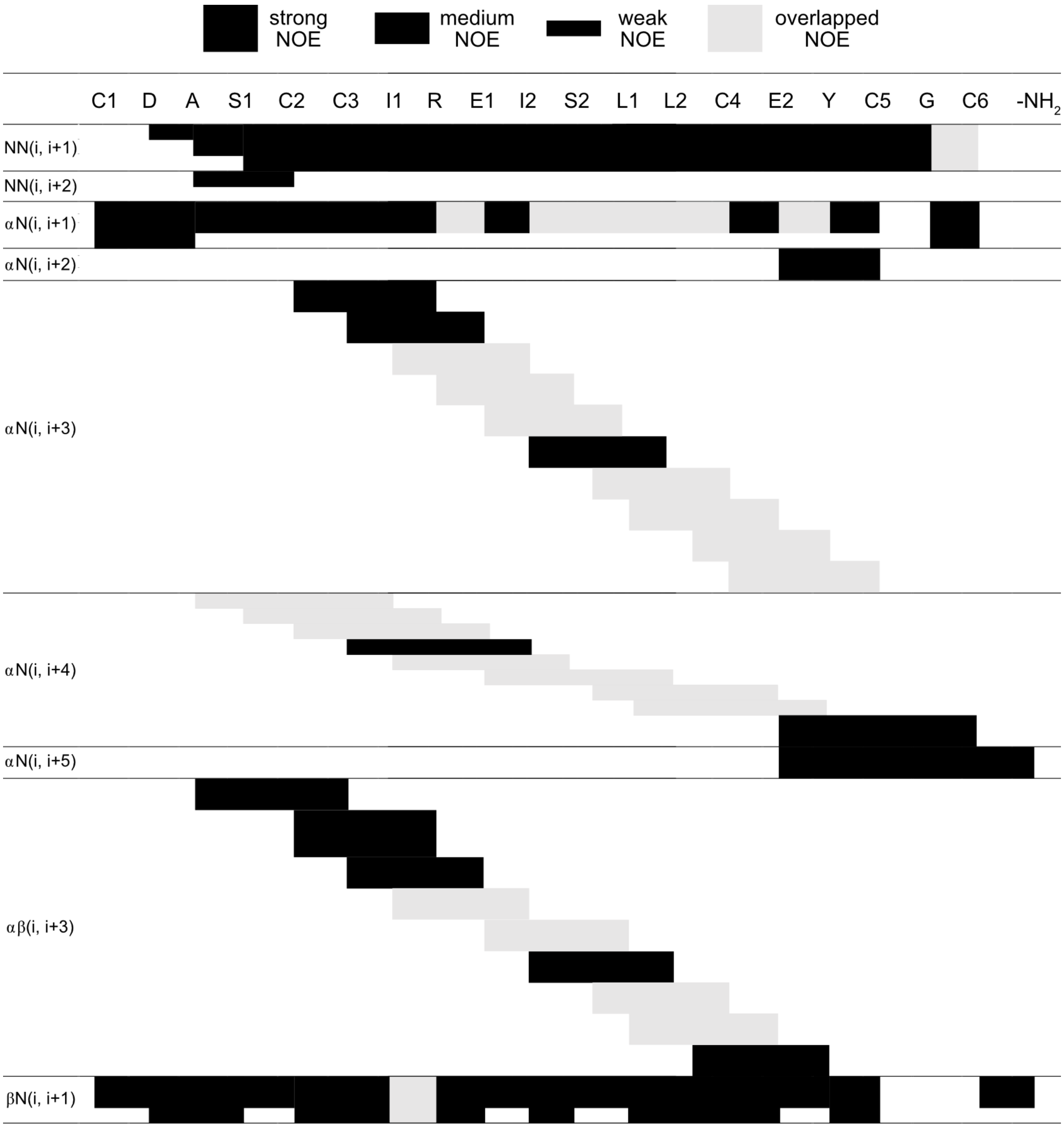

Figure S21 Characteristic cross-residue NOE in C-helix dual.

Ramachandran Plot of (θ, ψ) Angles

[C1-D-A-S1-C2-C3]$_{cyclo}$-I1-R-E1-I2-S2-L1-L2-[C4-E2-Y-C5-G-C6]$_{cyclo}$-NH$_2$

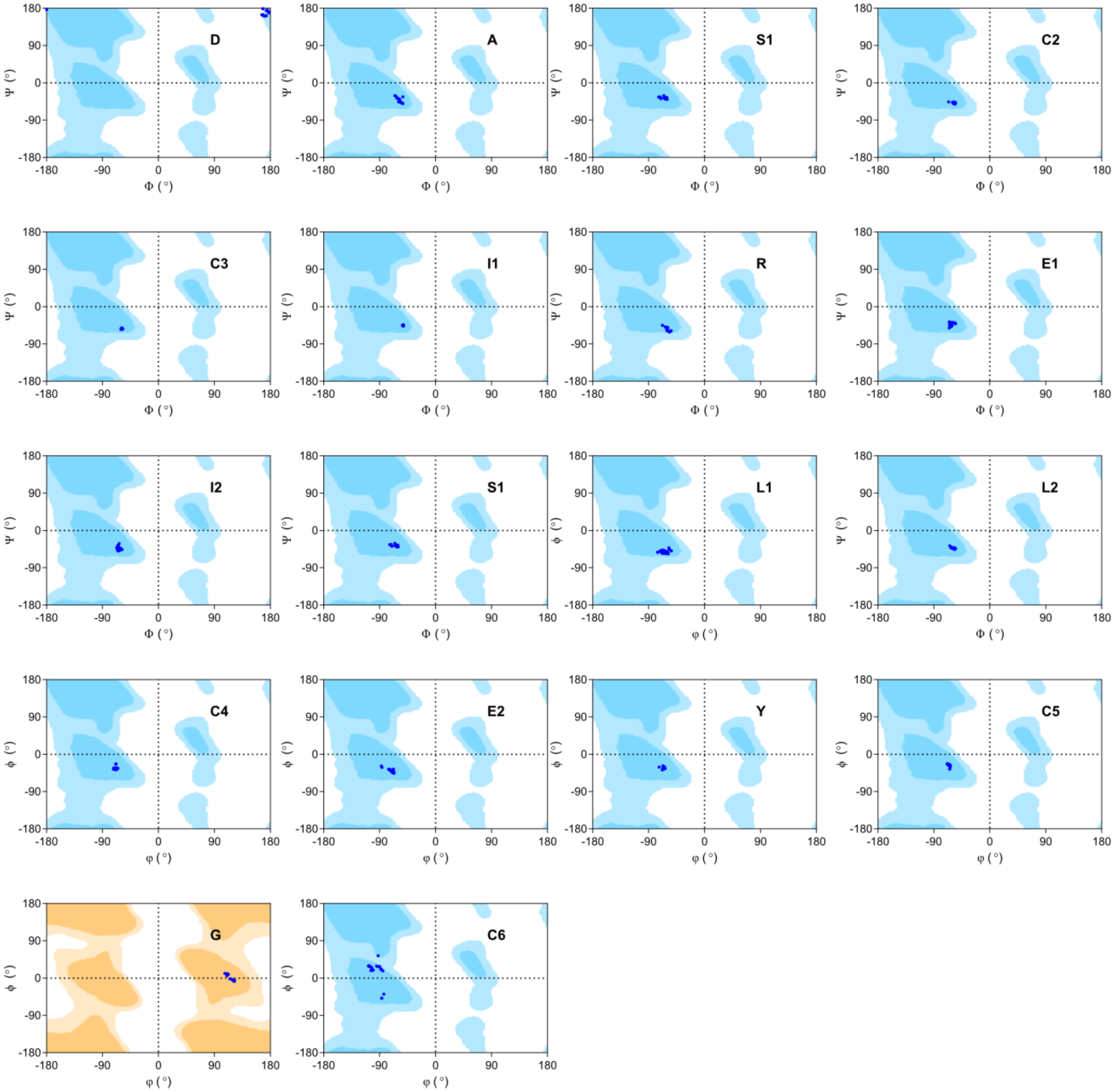

Figure S22 Dihedral angles (Φ,ψ) of residues in low energy conformers of C-helix dual. Blue/darker blue regions are general allowed/preferred regions for amino acids other than Gly and Pro. Orange/darker orange are allowed/preferred regions for Gly.

The Impacts of Shielding Effect from TMB to Nearby *H*s

As our previous manuscripts[8] have discussed, some *H*s at cap regions are near or underneath the TMB group, hence are affected by its shielding effect. Their proton chemical shifts are usually abnormally smaller than unaffected *H*s. For C-helix dual, similar observations were noticed. Two *H*s (C2-*NH*, C3-*NH*) at the BAMM region and three *H*s (C4-α*H*, G-*NH*, C6-*NH*) at the BSM region were found to have smaller chemical shifts than their colleagues. Besides, C1 is a non-helical residue (its cross-residue NOEs are more similar to β-structure), but it has a significantly negative Δδ of C1-α*H* in chemical shift index (Figure 4 in main text); this could also be explained that this *H* is affected by the shielding effect, hence is shifted to the upfield. After elucidation of the solution conformations of C-helix dual, simulated structures indicates that they are near or underneath the TMB groups, and those *H*s with more significant upfield shifts are unanimously closer to the TMB group (Δδ: C3-*NH* > C2-*NH*; C6-*NH* > G-*NH*), hence are more affected by the shielding effect. Therefore, experimental data from NMR spectra matches well with simulated solution conformations.

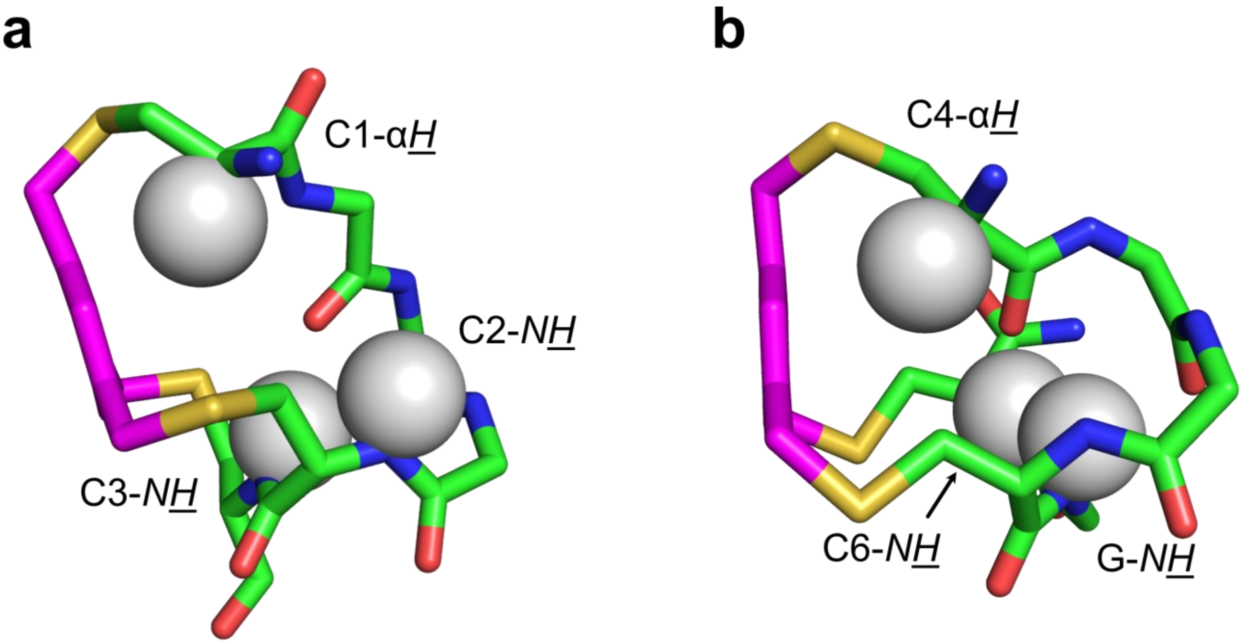

Figure S23 Side views of benzene ring and the six affected *H*s illustrate they are partially overlapped in the **a** BAMM *N*-cap and **b** BSM *C*-cap region.

H. In Vitro Peptide Stability in Human Serum

Male human serum (H-4522) was obtained from Sigma Aldrich (Missouri, USA). The tested peptide was mixed to make a final peptide concentration of 150 μM in 25 % human serum diluted in RPMI medium. Before the addition of the tested peptide, the diluted serum solution was temperature-equilibrated at 37 ± 1 °C for 15 min. The initial time is recorded as peptides were added. The mixture was incubated at 37 °C and an aliquot of 40 µL was removed at different time intervals. The aliquot was mixed with 160 μL cold methanol[14] and incubated at 4 °C for 15 min before centrifuging for 10 min to precipitate serum protein. The supernatant was analyzed in analytical RP-HPLC to calculate the amount of tested peptides, and in LCMS to analyze degradation products. The eluted peptides were detected by absorbance at 210 nm and quantified by their peak areas relative to the initial peak areas (0 h). Experiments were done twice, and the representative traces are shown below.

RP-HPLC Spectra and Potential Degradation Products of C-helix Peptides

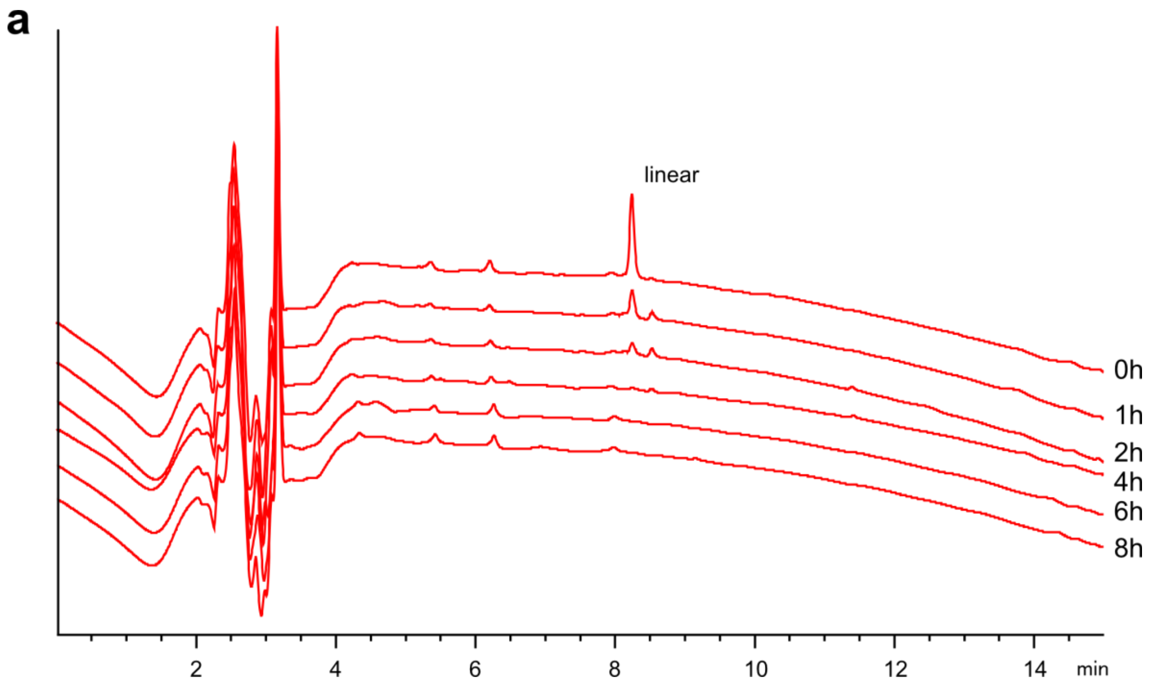

Figure S24 Overlaid RP-HPLC traces of **a** linear (0 - 8 hours), **b** BAMM, **c** BSM, **d** staple-1, **e** staple-2 from 0 to 48 hours, and **f** dual from 0 – 72 hours.

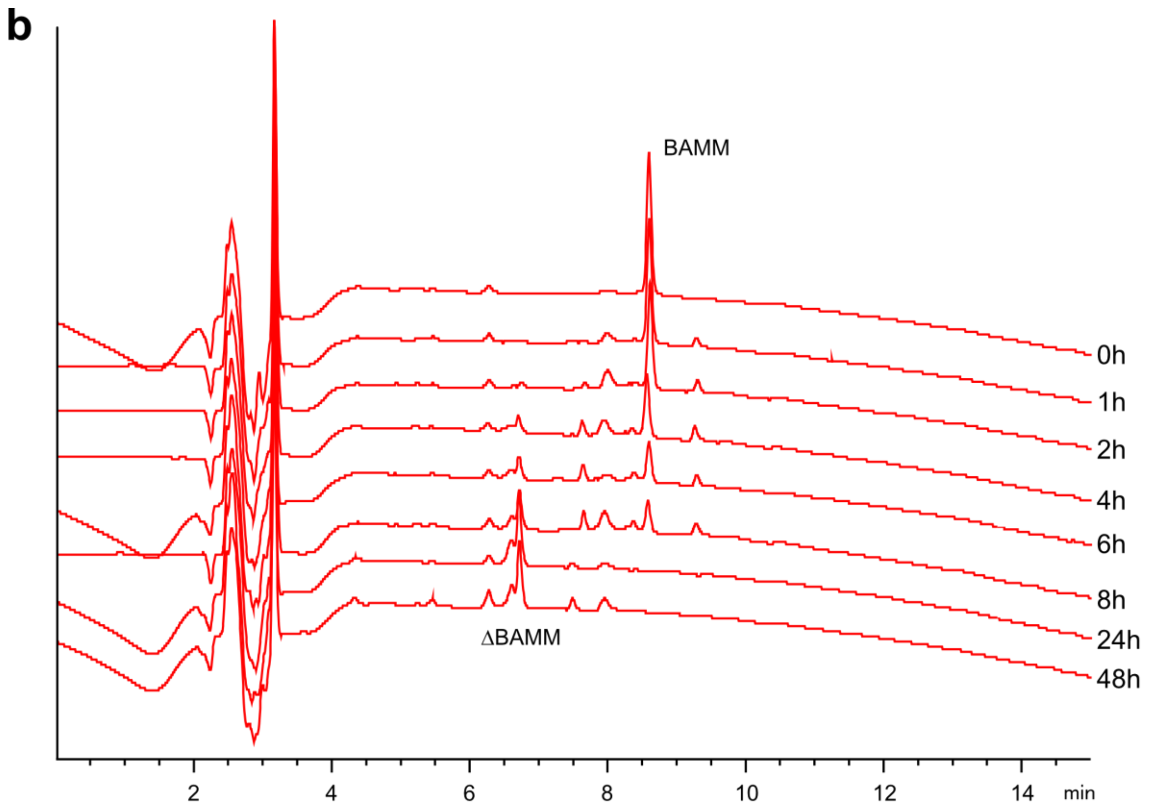

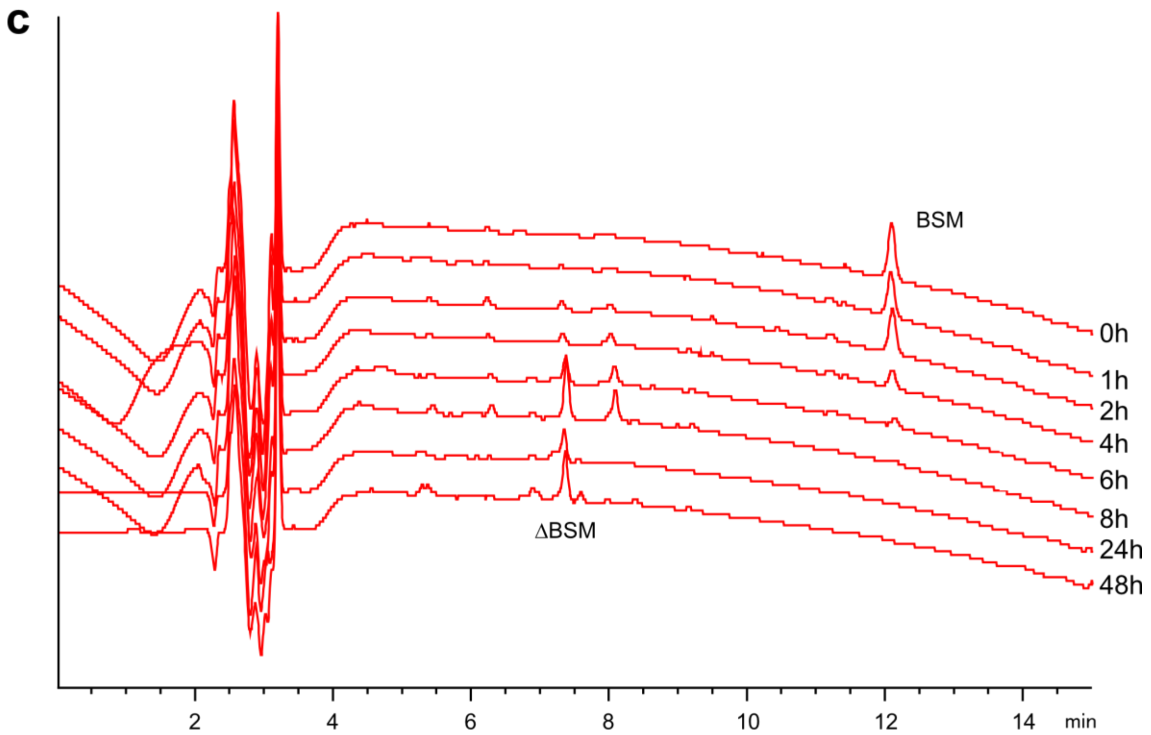

Figure S24 continued.

d

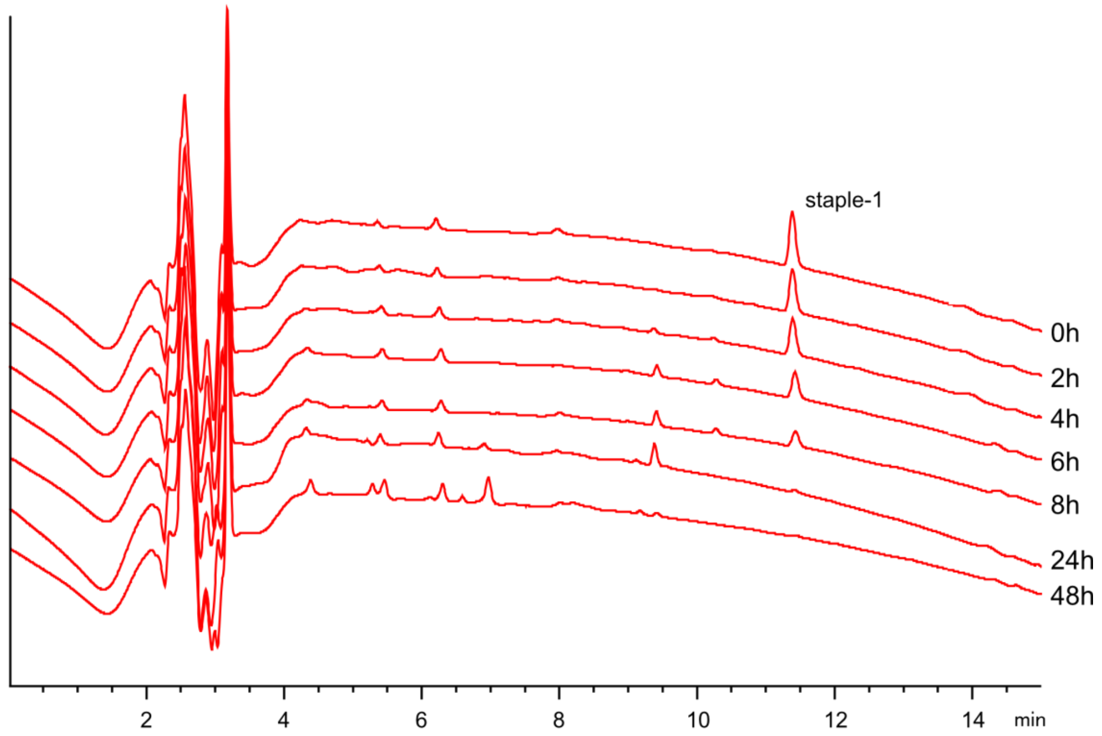

e

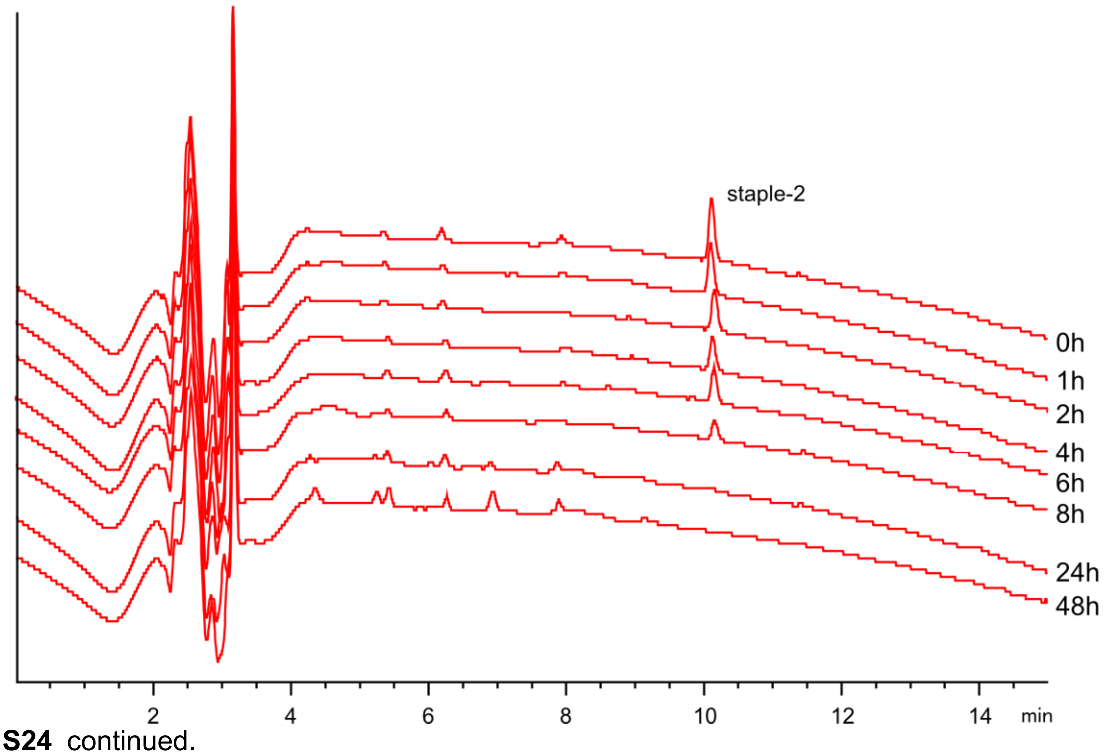

Figure S24 continued.

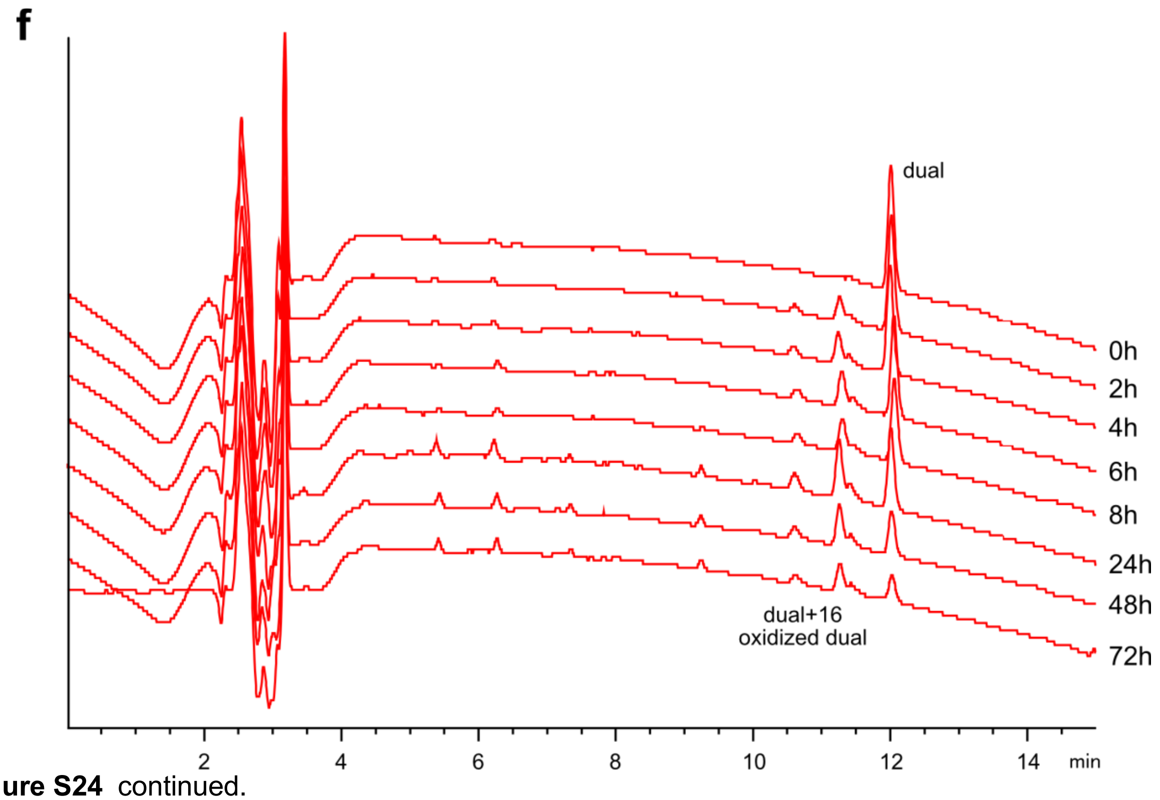

Figure S24 continued.

For linear peptide c-helix linear, no obvious remnants were found and it showed plain curves with several minor background peaks after 4 hours.

For BAMM, it gradually degraded to a stable intermediate, ΔBAMM, within 24 hours. ΔBAMM is highly stable in 25% human serum and did not obviously degrade to smaller fragments from 24 to 48 hours (Figure S25). Chemical formula of degradation product at 48 hours were speculated by data from LCMS.

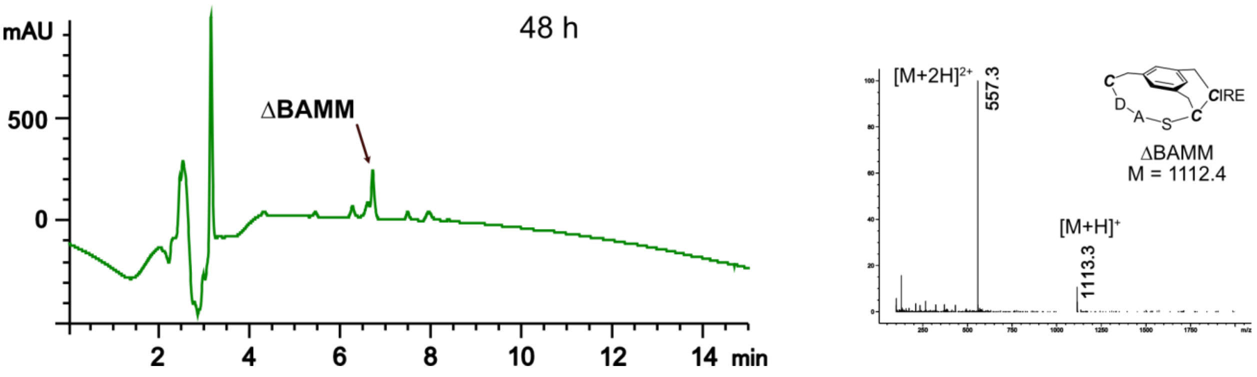

Figure S25 Analytical HPLC trace of BAMM degradation mixture at 48 hour time point, and the MS spectra of the major degradation product obtained from LCMS.

For BSM, it gradually degraded to one major product, ∆BSM. ∆BSM maintained a complete BSM bicyclic *C*-cap, and did not decrease in 48 hours. Chemical formula of degradation product can be speculated by mass spectra obtained from LCMS.

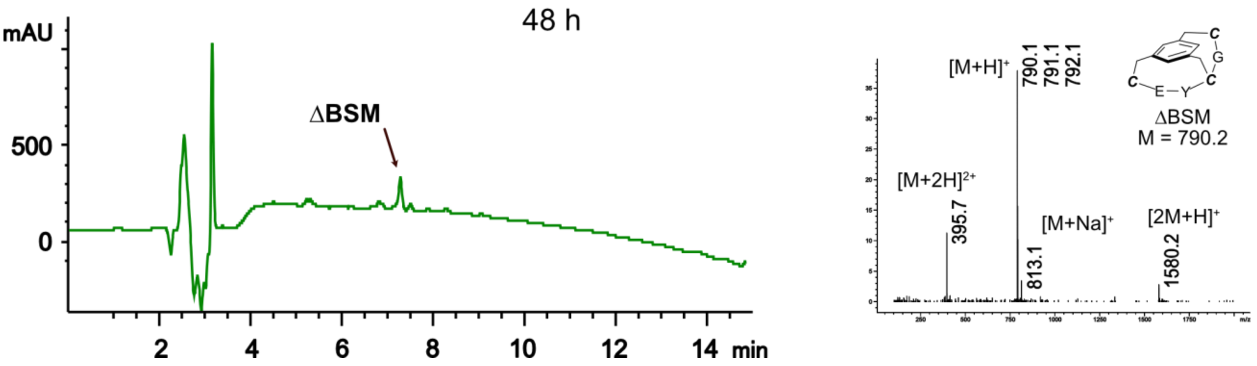

Figure S26 Analytical HPLC trace of BSM degradation mixture at 48 hour time point, and the MS spectra of the major degradation product obtained from LCMS.

For dual, it was highly stable in 48 hours. No detectable cleavage products were observed in LCMS. The only side product observed in LCMS is an oxidization product which had 16 Da mass more than dual. This peptide also showed similar oligomeric peaks in the LCMS, suggesting the backbone structures of dual was unchanged. The most vulnerable position for oxidization is the thiol ether in the cap regions; one of them were probably turned to sulfoxide in 48 hours.

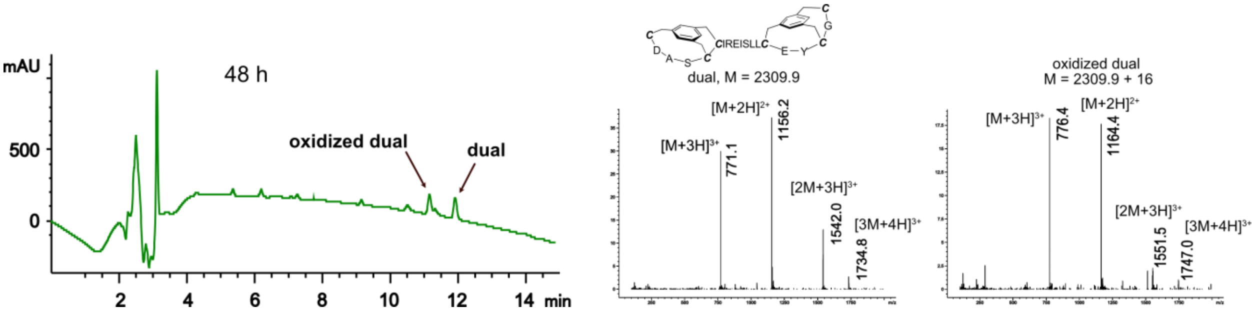

Figure S27 Analytical HPLC trace of dual degradation mixture at 48 hour time point, and the MS spectra of the major remnants obtained from LCMS.

For staple-1, multiple new peaks showed up on analytical HPLC after it was mixed with 25% human serum solution. At 48 hour time points, there were at least four major peaks with a few minor ones. At least five metabolic products were identified using LCMS, and their Molecular Mass were shown below. These degradation products can be classified into two classes: I, degraded peptides with intact ring; II, ring-open peptides. Initially we were unable to identify the peaks for the second classes, because we did not think the amides inside the hydrocarbon macrocycle could be hydrolyzed, but it was the only way to obtain these masses. We were surprised, and it suggested the hydrocarbon macrocycle was still partially flexible and allowed for hydrolysis ring opening. The elucidated sequences with their corresponding MS were shown below.

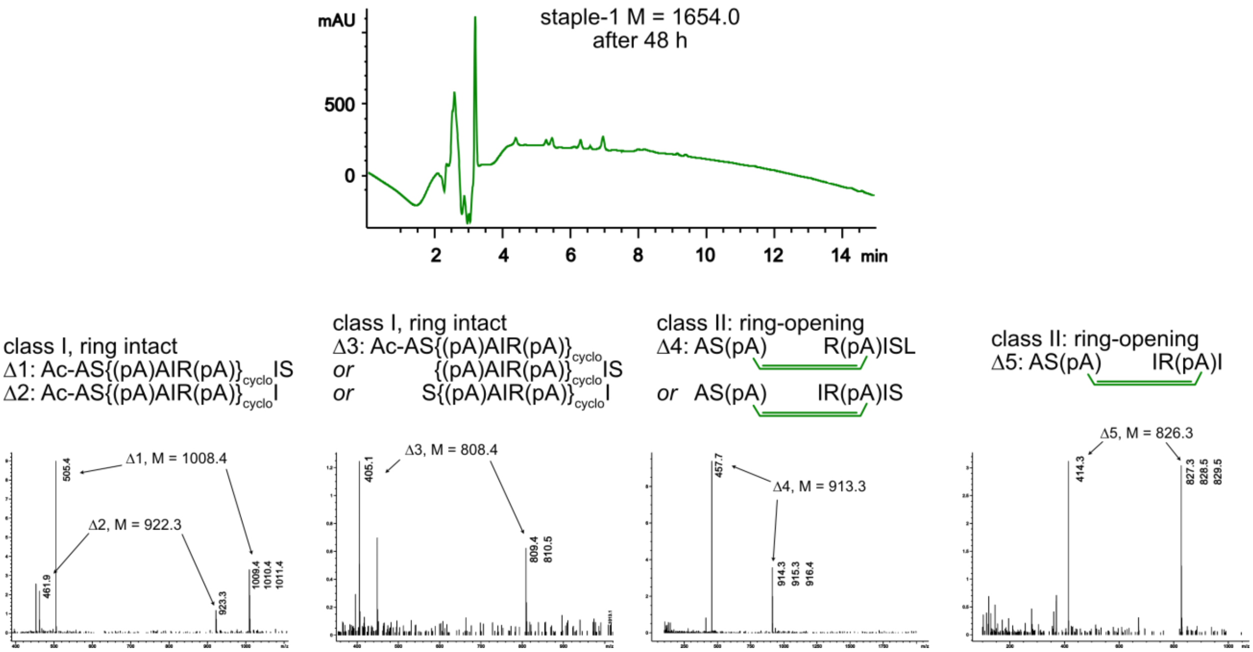

Figure S28 Analytical HPLC trace of staple-1 degradation mixture at 48 hour time point, and the MS spectra of the major remnants obtained from LCMS.

Similar situations happened for staple-2 peptide. Four metabolic products were identified in LCMS, which could be classified into two categories based on whether the hydrocarbon macrocycle was opened or not. The elucidated sequences with their corresponding MS were shown below.

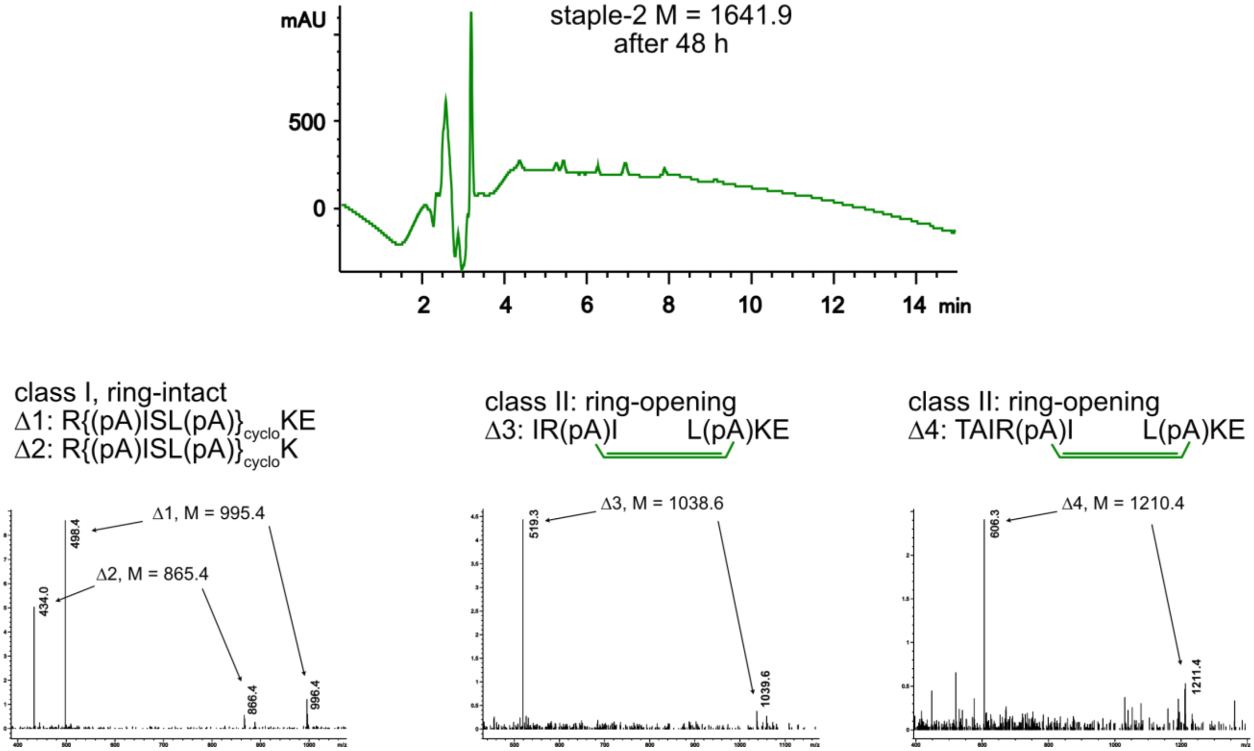

Figure S29 Analytical HPLC trace of staple-2 degradation mixture at 48 hour time point, and the MS spectra of the major remnants obtained from LCMS.

I. Fluorescence Polarization Assay

K_d of tested peptides were fitted based on following equations as previously reported in tutorials.[15,16]

- $P = f_{bound} * P_{bound} + f_{free} * P_{free}$
- $f_{bound} = \frac{a - \sqrt{a^2 - 4 * R_{total} * L_{total}}}{2 * L_{total}}$, $a = (K_d + R_{total} + L_{total})$
- $f_{bound} + f_{free} = 1$
    - $P = \frac{(I_{II} - I_I)}{(I_{II} + I_I)} = Y$, $R_{total} = X$
    - L_{total} *is the amount of tracer,* a constant
    - P_{bound}, P_{free} *and* K_d *are left for fitting in Graphpad Prism*

FITC labeled series peptides were prepared for assays associated with fluorescence. Their sequences and calculated binding affinities are in Table S5 below. Human Cyclin E with a His-GST tag, and cyclin A1 with a His tag were bought from Sino Biological. With limit amount of proteins, the highest final concentration of the protein was less than 3 μM in the assays. Linear, BAMM and BSM were not completely saturated, but staple peptides and dual managed to do so. C-helix linear was far away from the top boundary, so the equation above was unable to calculate K_d values for it. The others closer to at the top boundary were managed to obtain their K_d using this equation.

Table S5 Sequences and binding affinities of fluorescent labeled peptides.

Label	Sequence	K_d (μM)
linear-f	FITC-(β-Ala)$_3$-ASTAIREISLLKEY	-
BAMM-f	FITC-(β-Ala)$_3$-{CDASCC}$_{cyc}$IREISLLKEY	4.6 ± 2.4
BSM-f	FITC-(β-Ala)$_3$-ASTAIREISLL{CEYCGC}$_{cyc}$	4.9 ± 1.8
dual-f	FITC-(β-Ala)$_3$-{CDASCC}$_{cyc}$IREISLL{CEYCGC}$_{cyc}$	0.35 ± 0.06
staple-1-f	FITC-(β-Ala)$_3$-AS{(pA)AIR(pA)}$_{cyc}$ISLLKEY	0.13 ± 0.04
staple-2-f	FITC-(β-Ala)$_3$-ASTAIR{(pA)ISL(pA)}$_{cyc}$KEY	0.24 ± 0.10

The Procedure of Direct FP assay to Measure K_d of Fluorescent Peptides

Polarized fluorescence intensities were measured on Synergy H4 plate reader with excitation and emission wavelengths of 485 and 535 nm with 20 nm band widths, respectively. The optics position is Top 510 nm with Gain set as 100. Read Speed is normal and read height is 7 mm. Cyclin proteins were bought from Sino Biological and after reconstitution it should be used immediately. Leaving proteins in freezer for weeks could yield either precipitations or significantly increased background fluorescence polarizations. To each well, 10 μL 40 nM fluorescent peptides in PBS was added. Various concentrations of cyclin E or cyclin A1 were prepared (0.16 – 4.8 μM for cyclin E; 0.18 – 5.4 μM for cyclin A1) and 10 μL were added to each well. The plate was shaken in the dark for 3 h. Wells containing only the peptide and PBS buffer were used as a control. Each mixture was prepared in duplicates. K_d values and error bars of tested peptides were fitted by the equation above in GraphPad Prism.

Data of FP assays for the rest of compounds against cyclin E is shown below.

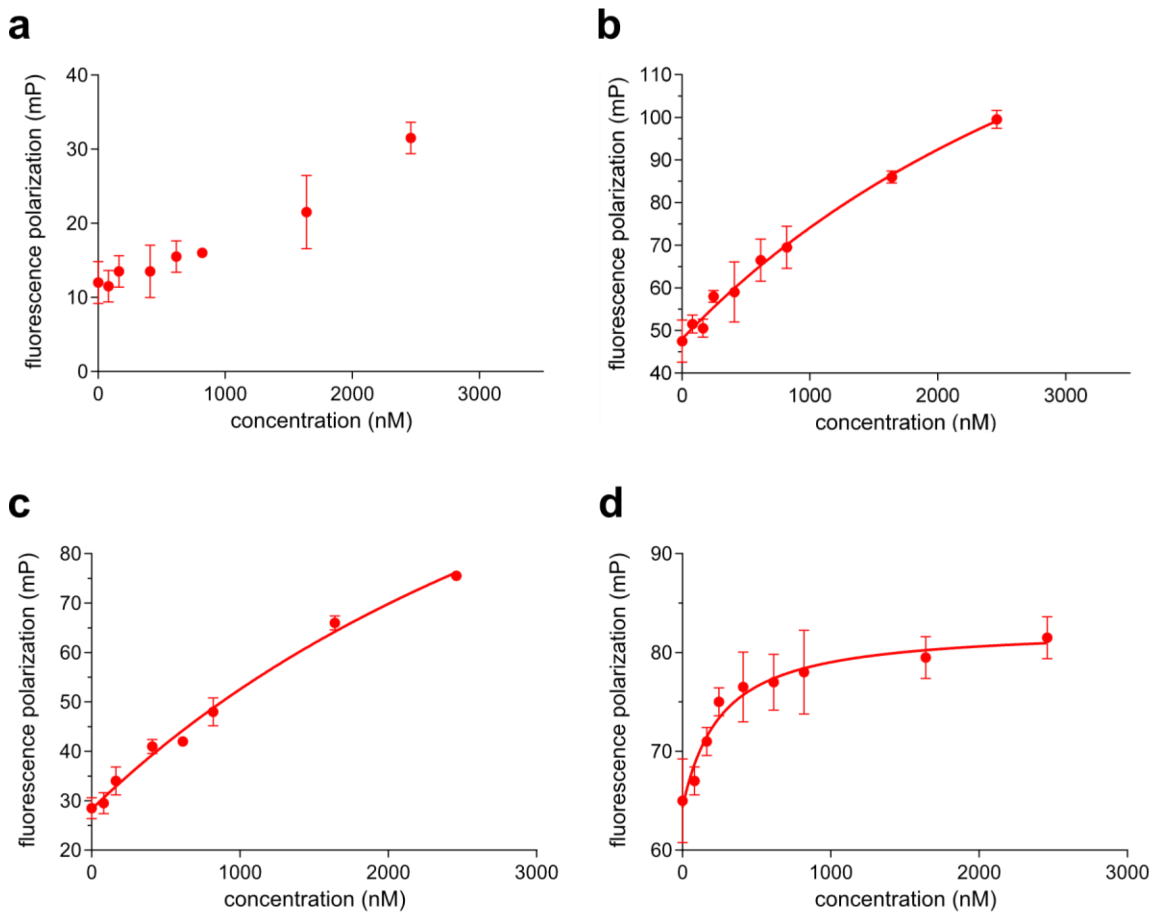

Figure S30 Raw data of fluorescence polarization assays for **a.** linear-f, **b.** BAMM-f, **c.** BSM-f, **d.** staple-2-f.

For cyclin A1, only the linear peptide was tested for it as a negative control.

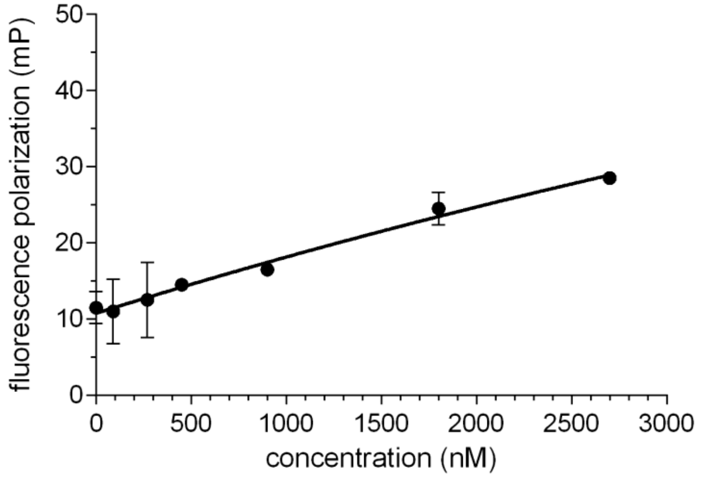

Figure S31 Raw data of fluorescence polarization assays for linear-f targeting cyclin A1.

J. Fluorescence Quenching Assay

Fluorescence quenching of the single tryptophan in cyclin E was measured by using a Cary Eclipse (Varian) equipped with a front-face fluorescence accessory at 25°C, and 10-nm excitation and 10 nm emission bandwidths. The excitation wavelength was 295 nm and the emission spectra were measured between 315 and 395 nm. Titrations were performed in a 0.7-ml quartz fluorescence cuvette containing 0.5 mL 0.82 μM protein in PBS buffer, pH 7.4, and by the successive addition of 0.5~1 μL compound stock solutions. After each addition, the cuvette was shaken (with cap on) to enable fast mixing, and there were 10 mins interval between two additions to ensure equilibrium of the mixture. The measurements were repeated at least three times at each concentration. Data was analyzed by plotting the relative fluorescence intensities at 338 nm at increasing concentrations of quencher. Dissociation equilibrium constant (K_d) values were determined from data fitted to the equation mentioned in Yammine et al.[17]

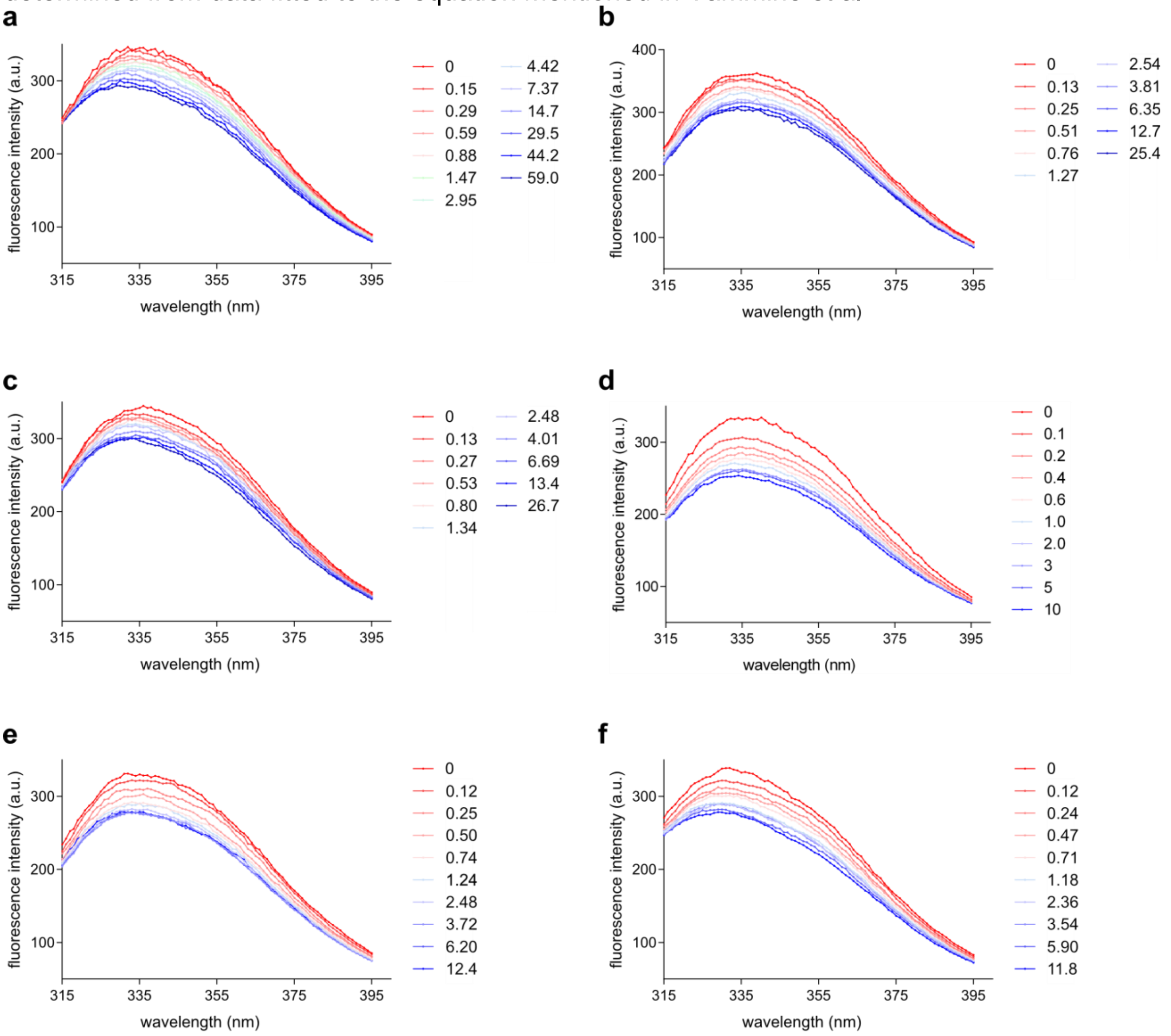

Figure S32 Raw data of fluorescence quenching assays for **a.** linear, **b.** BAMM, **c.** BSM, **d.** dual, **e.** staple-1 and **f.** staple-2.

K. Cell Culture and Cellular Uptake Assay

Cell Culture

HepG2 and MCF-7 cells were grown in Dulbecco's Modified Eagle's medium (DMEM) containing 10% fetal bovine serum (FBS) and 1% Penn Strep. MDA-MB-231 cells were grown in DMEM/F12 media with 10% FBS. Cells were grown in an incubator at 37°C, humidified atmosphere containing 5% CO2. Cells were grown in T-75 culture flask until 90% confluency before splitting into next passage.

Cellular Uptake Assay

Cells were seeded into a 24-well plate with a density of 200,000 cells/well and allowed to adhere overnight before treated with different concentration of compounds. After incubation at 37 °C for certain fixed time, the cells were washed with PBS twice and treated with 100 µL trypsin solution per well over 5 min at 37 °C for detachment. 200 µL media were added to each well and transferred to 96 well plates, which were analyzed by flow cytometer Cytoflex. The FITC channel was used to detect FITC labeled peptides. Live single cells were gated, and then the fluorescent intensity was recorded and input into Graphpad Prism 10 for analysis and graphic presentation. 0.1 % DMSO vehicle was used as a negative control to be subtracted as background fluorescence.

Confocal Imaging

Accumulation of FITC labeled peptides in cells was monitored by Leica SP8. Briefly, 50,000 cells/0.3 mL/well were seeded on 8 well µ-slides (ibidi #80826) and allowed to adhere overnight. The cells were incubated with fixed concentration of fluorescent peptides for 4 h or 24 h at 37 °C. The cells were then washed with PBS twice, and media was added for live cell imaging. The images were taken using 40x/0.75 water immersed objective. The samples were excited at 488 nm and detected at 500-600 nm for fluorescent channel. DIC channel was also recorded.

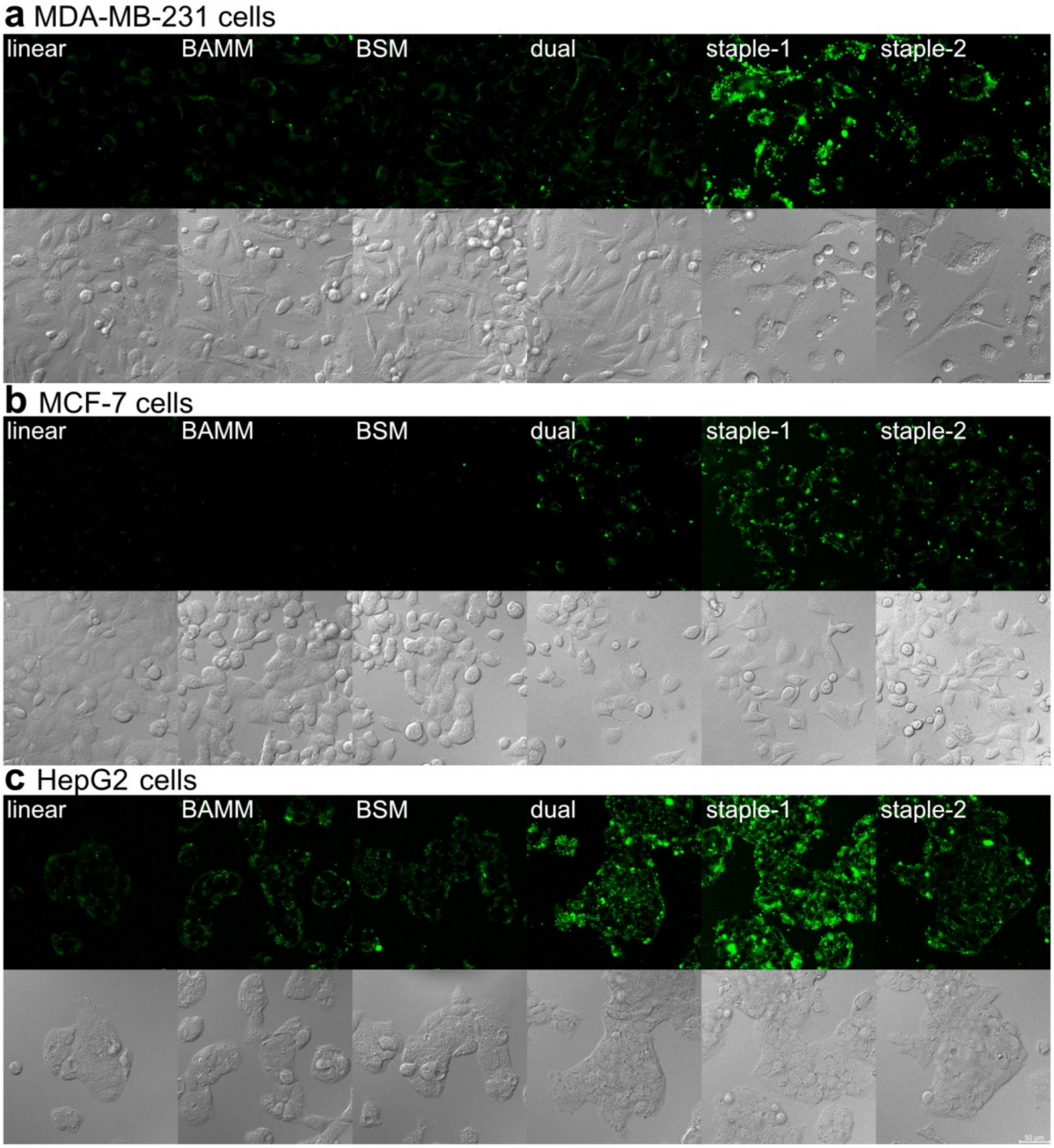

Figure S33 Confocal imaging of MDA-MB-231 (**a**), MCF-7 (**b**) and HepG2 (**c**) cells treated with linear, BAMM, BSM, dual, staple-1 and staple-2 (1 μM, 4 h) at 37°C, respectively.

Blotting Experiments for Cyclin E

Cells (MDA-MB-231, MCF7, HepG2) were seeded to a 24-well plate at a density of 250,000 cells/500 µL/well respectively, and allowed to adhere overnight. The cells were washed with DPBS twice and lysed with RIPA buffer containing 1% protease inhibitors (50 µL per well) on ice over 30 min. Lysate were then transferred to Eppendorf tubes and centrifuged at 19000 rpm for 30 min at 4°C. The supernatant was collected and the total protein concentration is determined using Pierce BCA protein assay kit (Cat# 23225). The following blotting experiments were conducted on Jess (https://www.proteinsimple.com/jess.html). Jess is a novel technology that automates the protein separation and immunodetection of traditional Western blotting. 0.8 mg/ml of cell lysates were loaded on Jess. Anti-Cyclin E polyclonal antibody (cat#10902-RP02 from Sinobiology) was used at 1/2000 dilution as the primary antibody whereas Anti-rabbit HRP antibody as the secondary antibody.

Another band above Cyclin E band was observed only in MDA-MB-231 cells (Figure S34**a**). We believe this is because it's a polyclonal antibody, which might bind to other proteins that may share the similar conformation as Cyclin E. This result is similar to the vendor product report where it also have multiple non-cyclin E bands on the gel (Figure S34**b**).

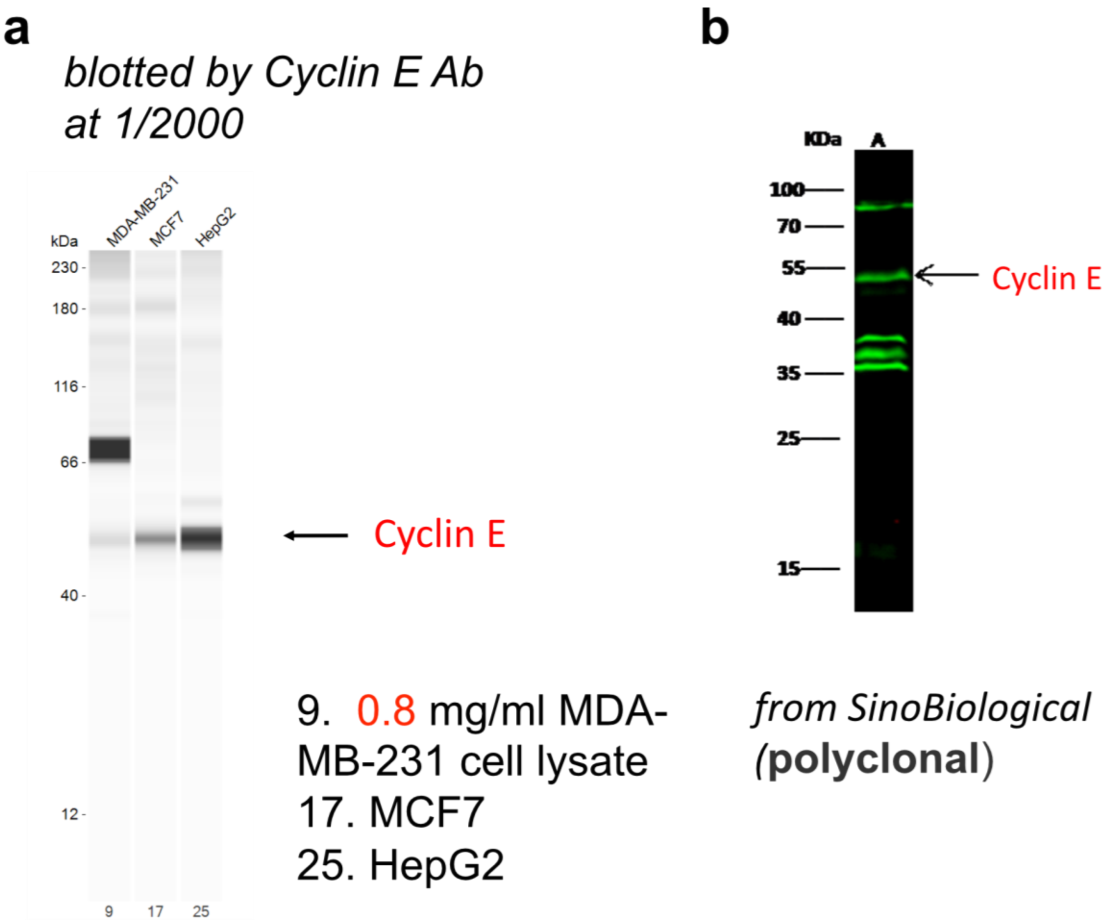

Figure S34 Blotting experiment on cyclin E in three different cell lines using Jess from our lab (**a**) and in U87 cells provided by Sino Biological (**b**).

Fluorescence Spectra of Tested Peptides from 50 to 200 nM

Stock solutions of the six fluorescent compounds were prepared by dissolving them in DMSO. Their concentrations were calibrated by measuring their absorbance (1uL stock + 3 mL PBS buffer mixture) at 495 nm, ε = 74000 ($M^{-1}cm^{-1}$) were used, and the concentration of stock solution is calculated by: c = (A*3001/74000) * 1000 (mM). Samples in 50 nM, 100 nM, 150 nM and 200 nM were prepared for the six fluorescent compounds. No higher concentrations were selected because the readings, fluorescence intensity (a.u.), exceed the top limit (1000 a.u.) for some peptides at higher (>200 nM) concentrations. Emission wavelength was set as 488 nm, and fluorescence spectra collected between 505 and 600 nm. Base lines were collected and subtracted by the other spectra to give data of the tested peptides shown below.

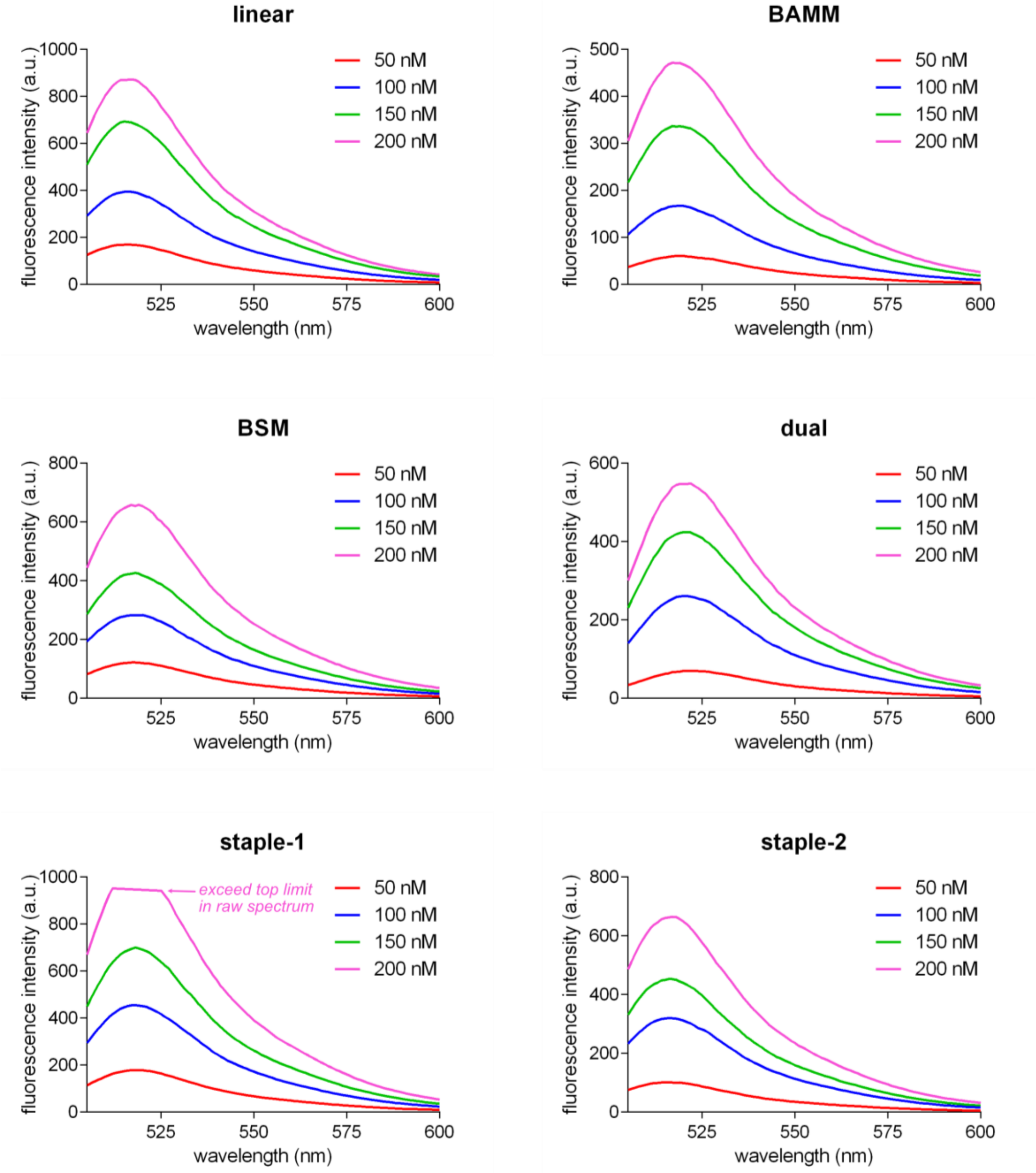

Figure. S35 Fluorescence spectra of six fluorescent compounds from 50 to 200 nM.

L. Characterization of Purified Peptides

Purity of peptides were measured in analytical HPLC runs using a Zorbax SB-C18 column (Agilent) with a 20 minute gradient between {5% solvent A (99.9% water, 0.1% TFA), 95% solvent B (99.9% acetonitrile, 0.1% TFA)}, and {95% solvent A, 5% solvent B}. Absorption traces at 210 nm were presented along with the retention time for different peptides. Expected masses of peptides were calculated from ChemDraw, and observed masses are $[M+H]^+$ or $[M+2H]^{2+}$ peaks from ESI-MS spectra.

linear retention time: 11.049 min calculated $[M+H]^+$: 1634.91 Observed $[M+H]^+$: 1634.9097

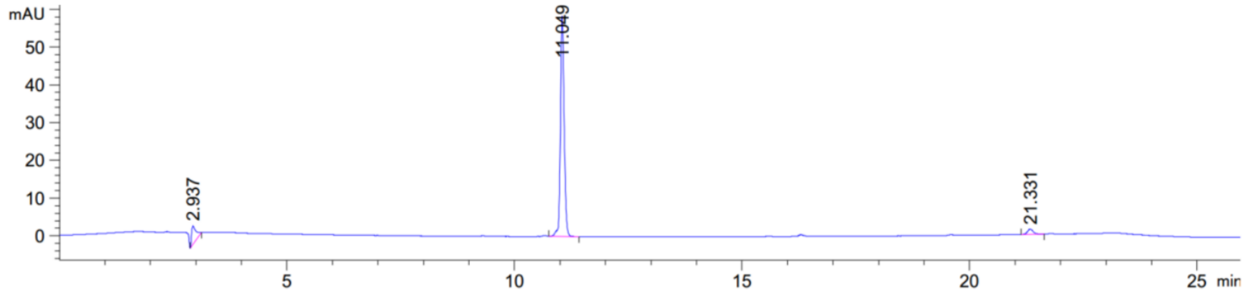

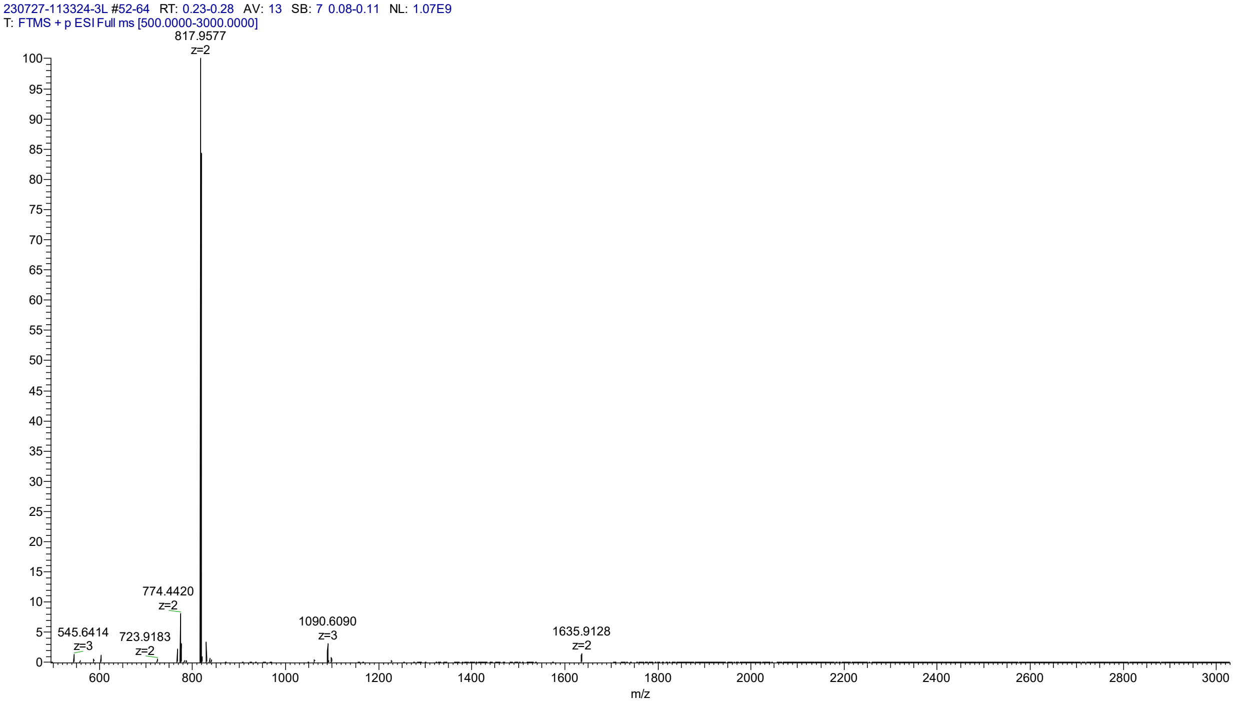

BAMM retention time: 11.394 min calculated $[M+H]^+$: 1958.92 Observed $[M+H]^+$: 1958.9244

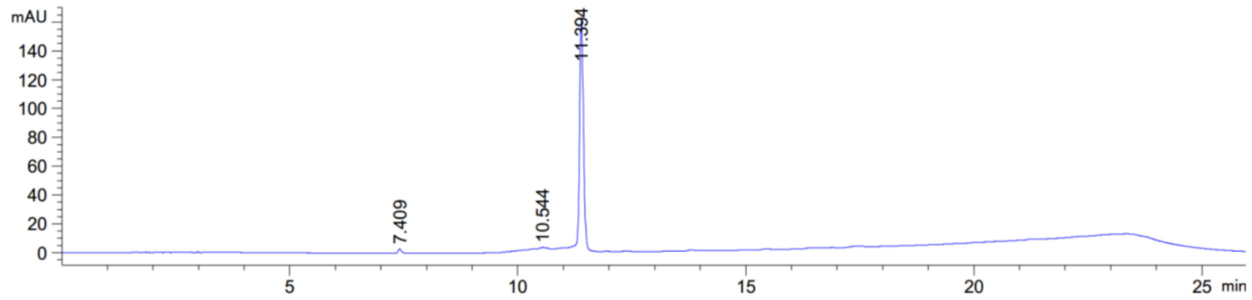

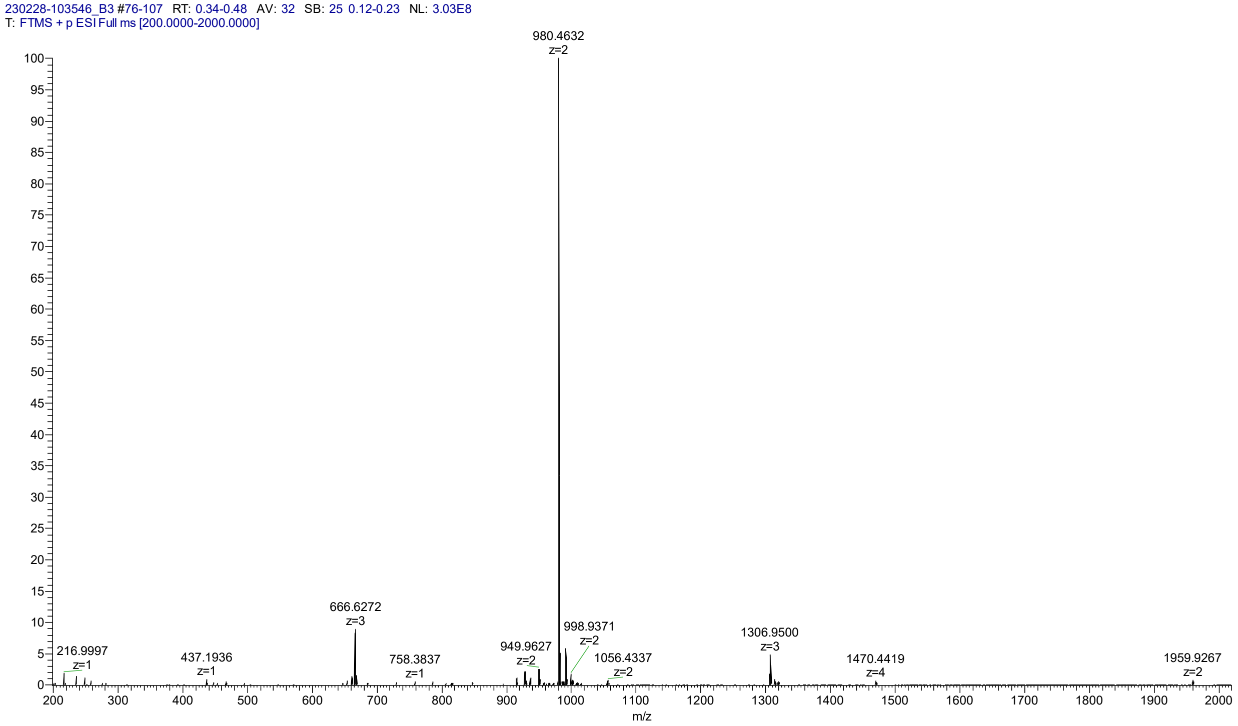

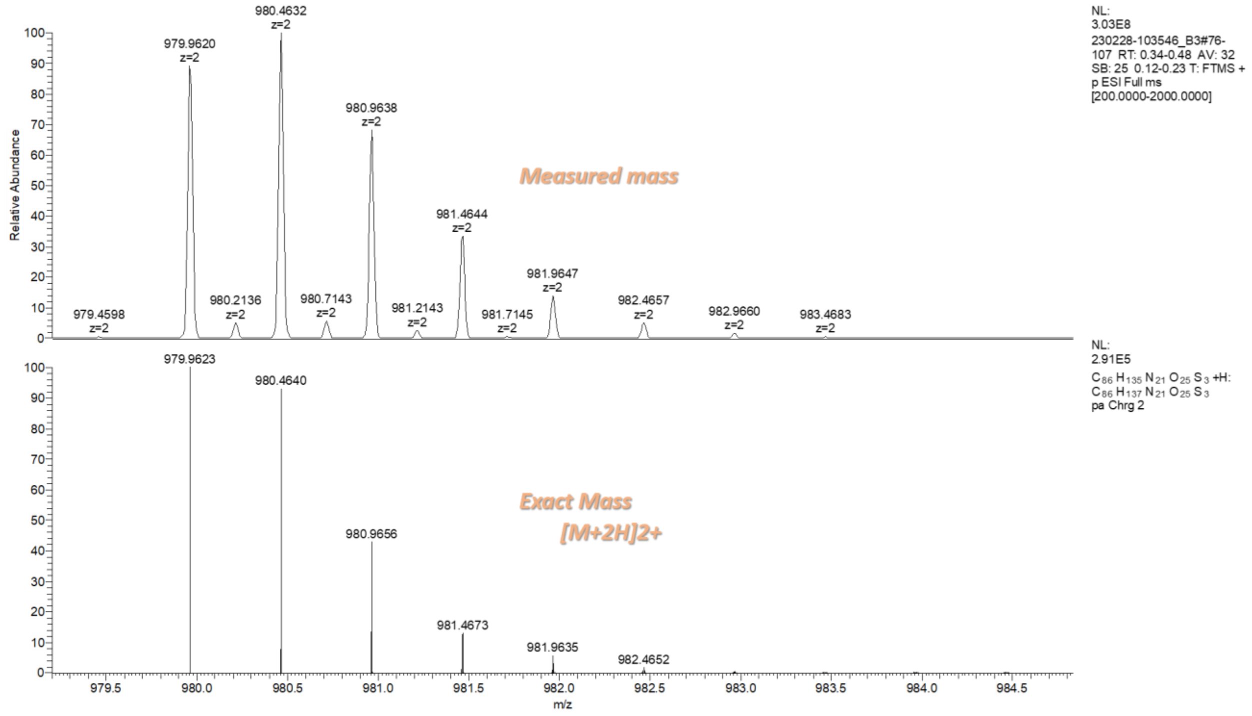

BSM retention time: 16.826 min calculated $[M+H]^+$: 1986.91 Observed $[M+H]^+$: 1986.9150 Observed $[M+2H]^{2+}$: 993.9597

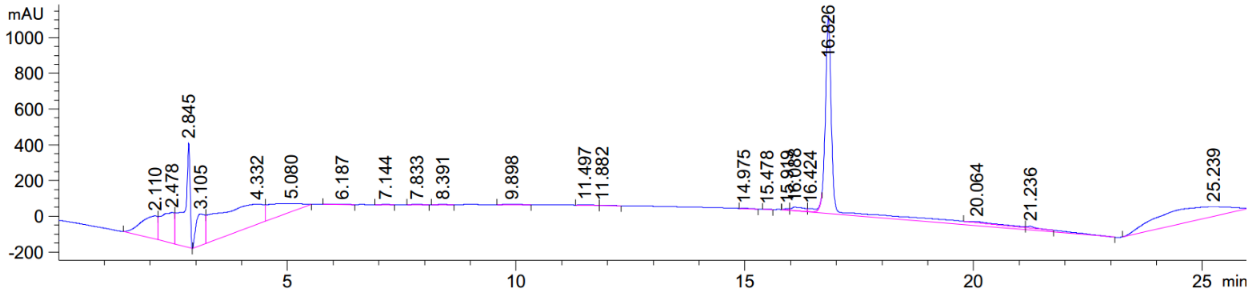

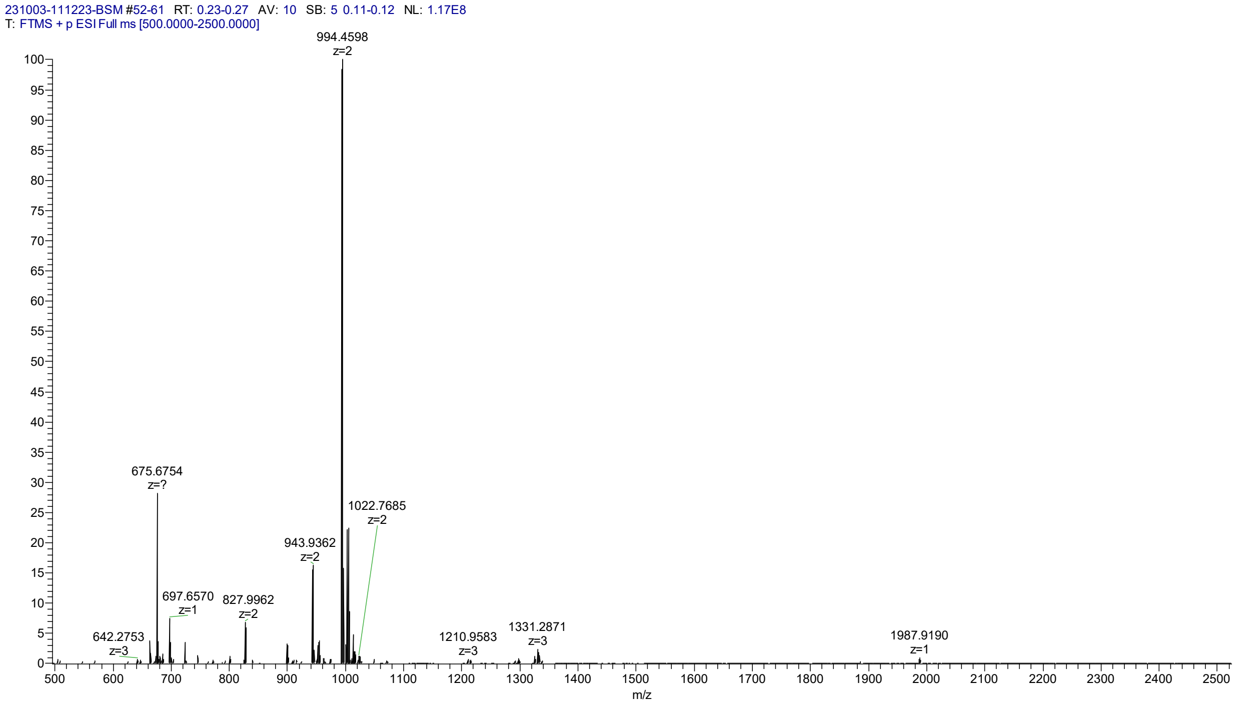

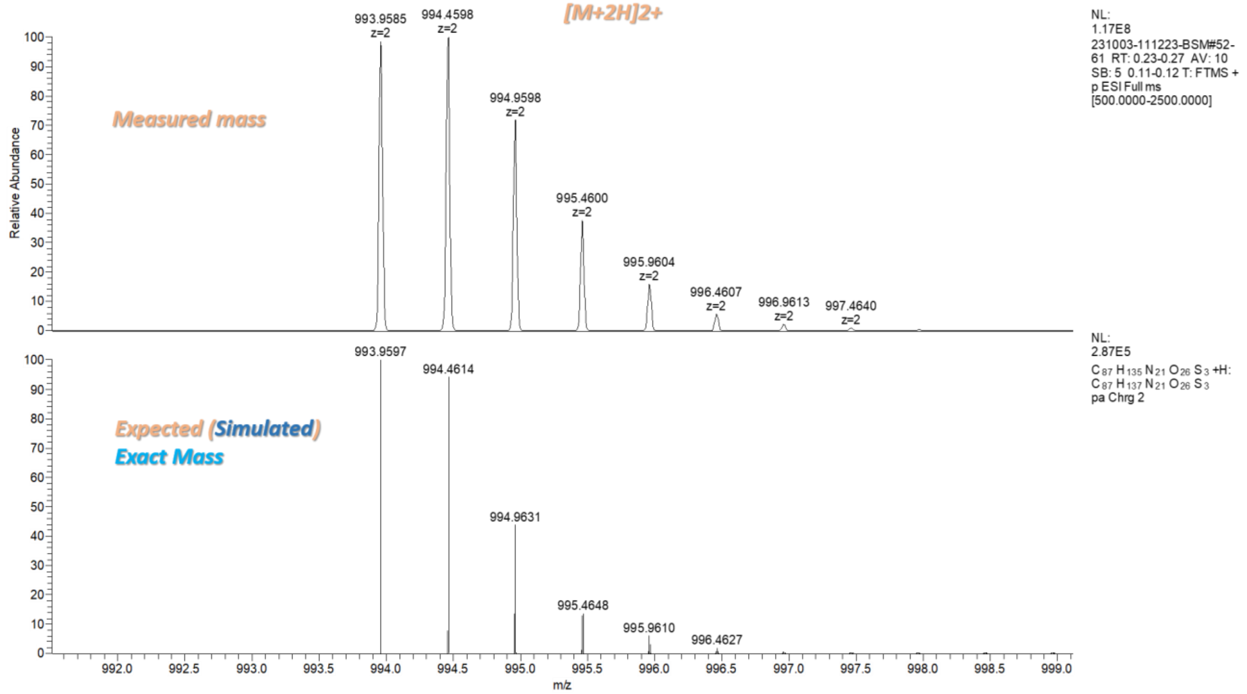

dual retention time: 16.891 min calculated $[M+H]^+$: 2310.92 Observed $[M+H]^+$: 2310.9261 Observed $[M+2H]^{2+}$: 1155.9612

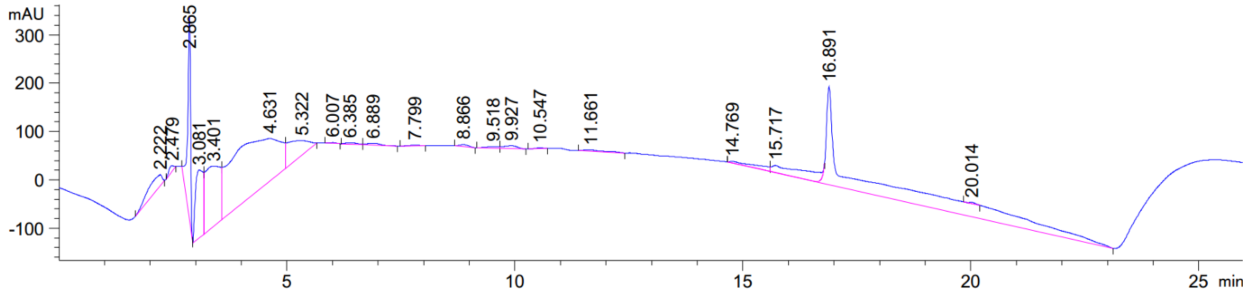
mAU
2.865
16.891
20.014
min

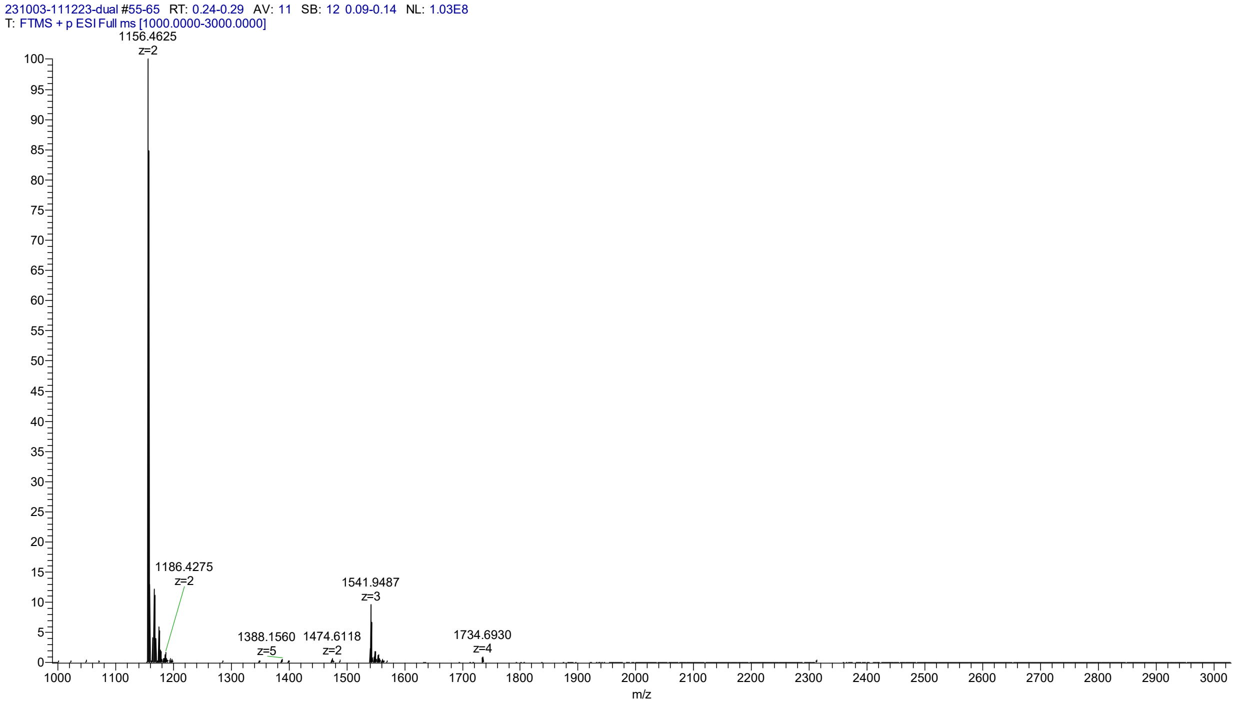
231003-111223-dual #55-65 RT: 0.24-0.29 AV: 11 SB: 12 0.09-0.14 NL: 1.03E8
T: FTMS + p ESI Full ms [1000.0000-3000.0000]
1156.4625
z=2
1186.4275
z=2
1541.9487
z=3
1388.1560
z=5
1474.6118
z=2
1734.6930
z=4
m/z

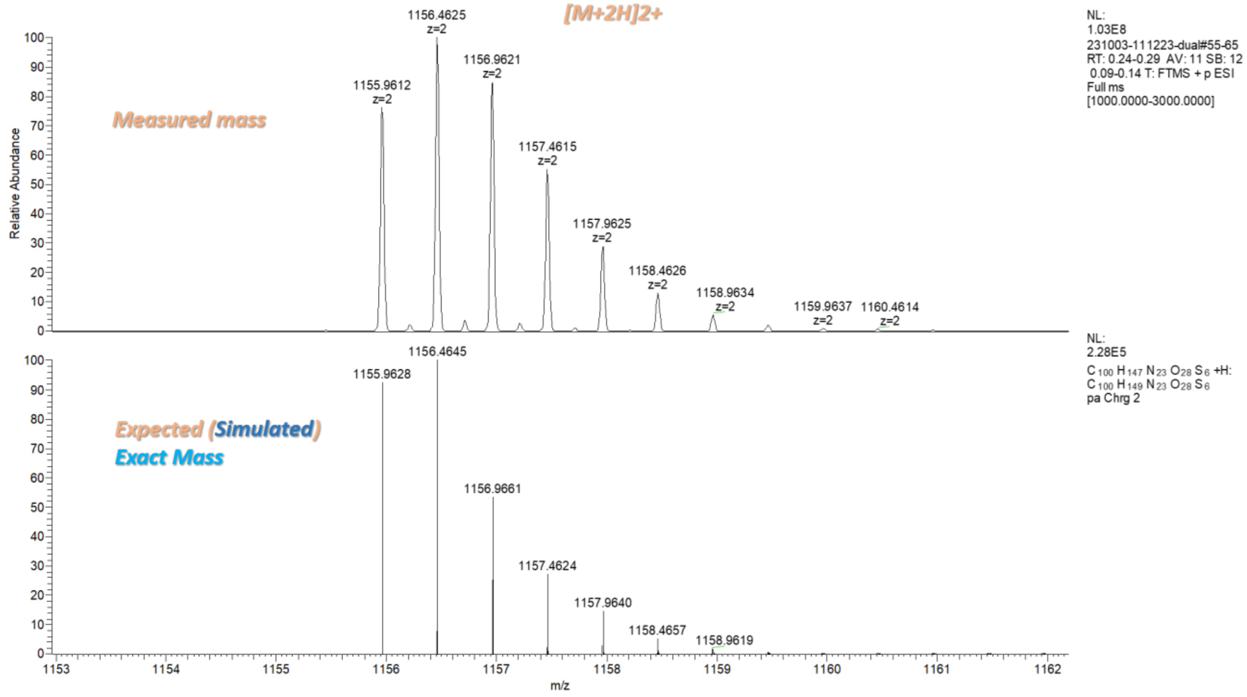
[M+2H]2+
Measured mass
Relative Abundance
1156.4625
z=2
1156.9621
z=2
1155.9612
z=2
1157.4615
z=2
1157.9625
z=2
1158.4626
z=2
1158.9634
z=2
1159.9637
z=2
1160.4614
z=2
NL:
1.03E8
231003-111223-dual#55-65
RT: 0.24-0.29 AV: 11 SB: 12
0.09-0.14 T: FTMS + p ESI
Full ms
[1000.0000-3000.0000]
1156.4645
1155.9628
1156.9661
1157.4624
1157.9640
1158.4657
1158.9619
Expected (Simulated)
Exact Mass
NL:
2.28E5
C 100 H 147 N 23 O 28 S 6 +H:
C 100 H 149 N 23 O 28 S 6
pa Chrg 2
m/z

staple-1 retention time: 15.863 min calculated $[M+H]^+$: 1654.99 Observed $[M+H]^+$: 1654.9877 Observed $[M+2H]^{2+}$: 827.9964

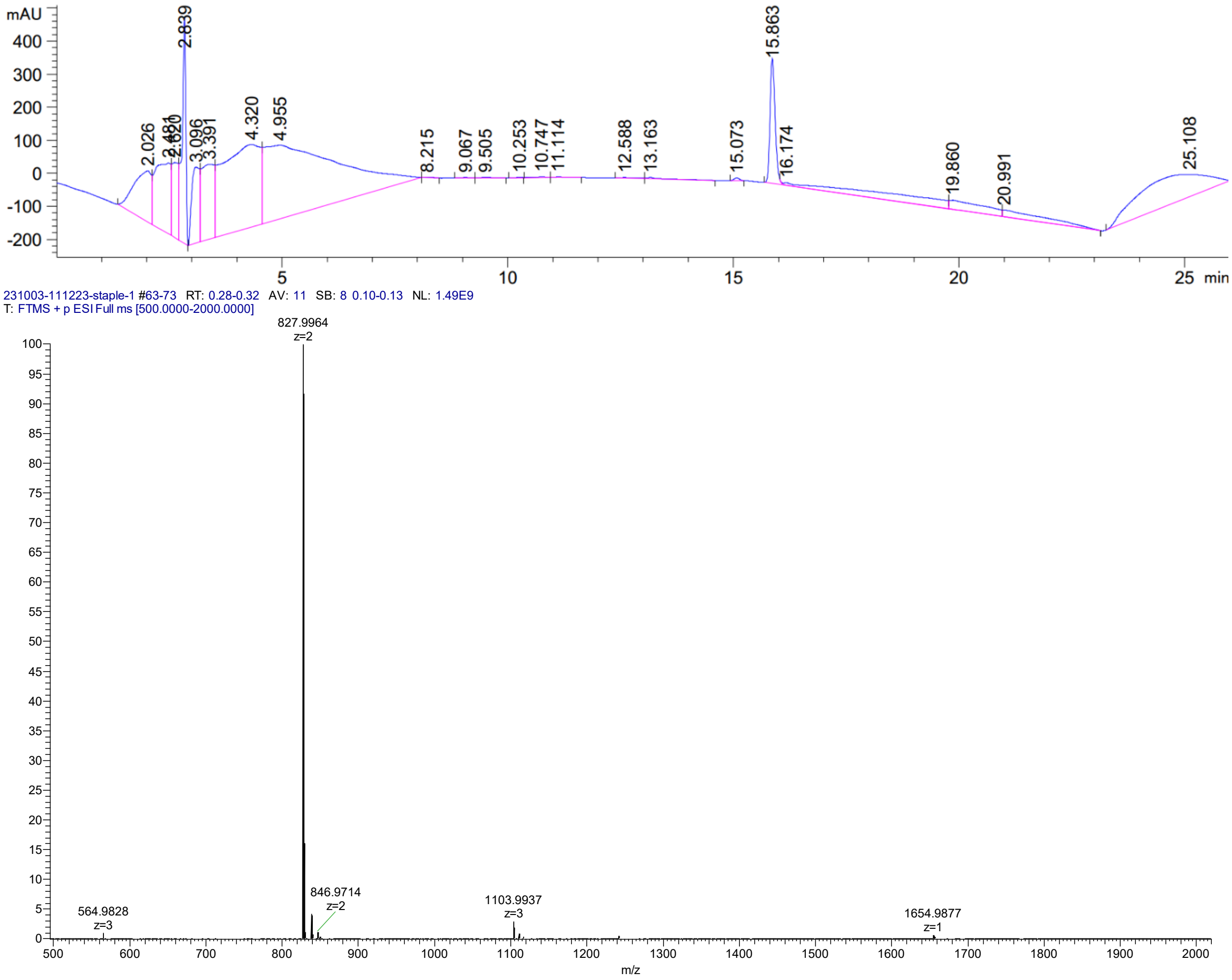

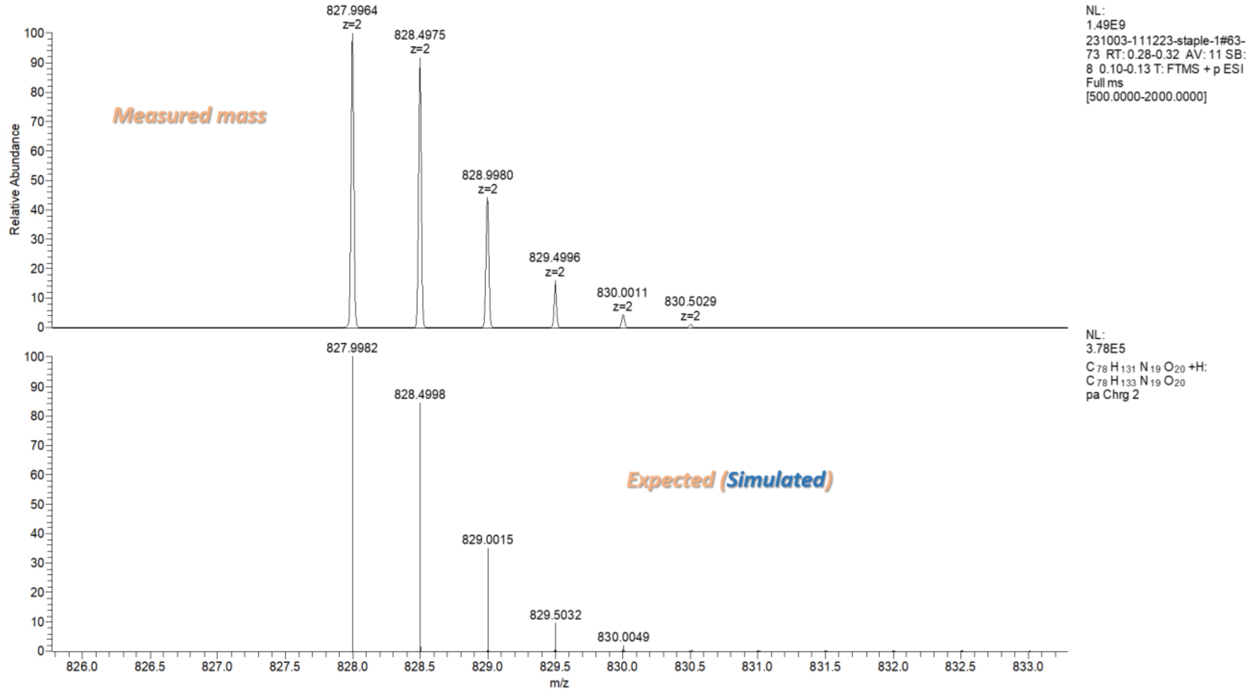

staple-2 retention time: 13.971 min calculated $[M+H]^+$: 1642.95 Observed $[M+H]^+$: 1642.9500 Observed $[M+2H]^{2+}$: 821.9782

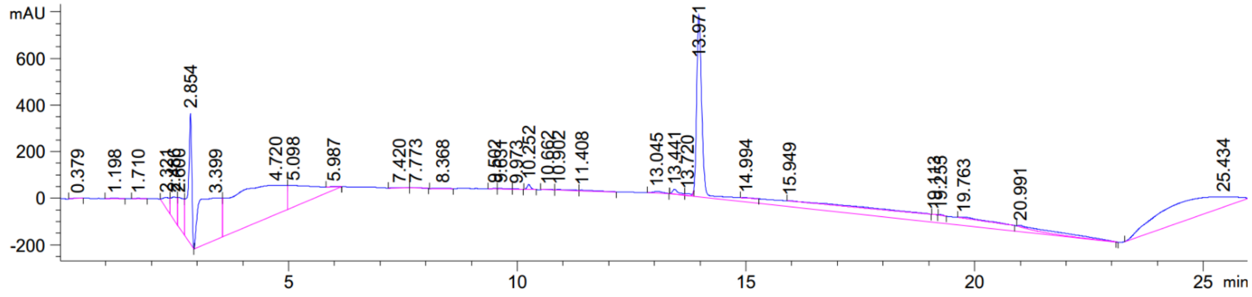

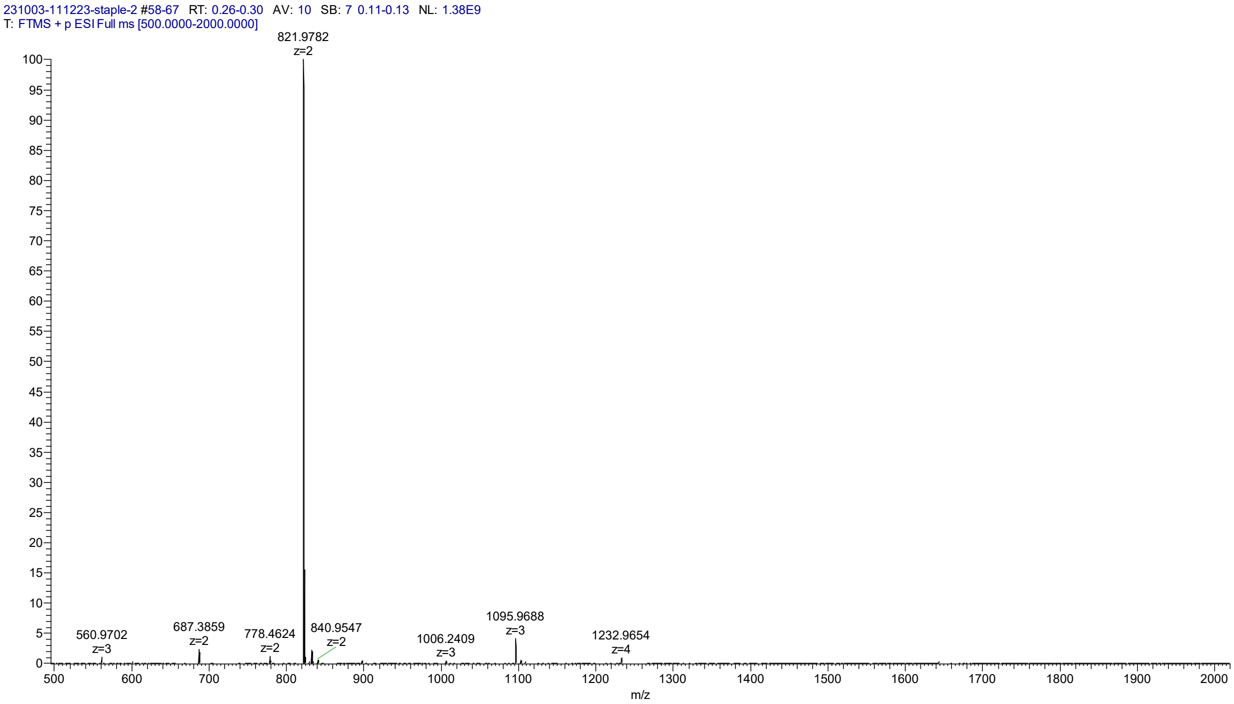

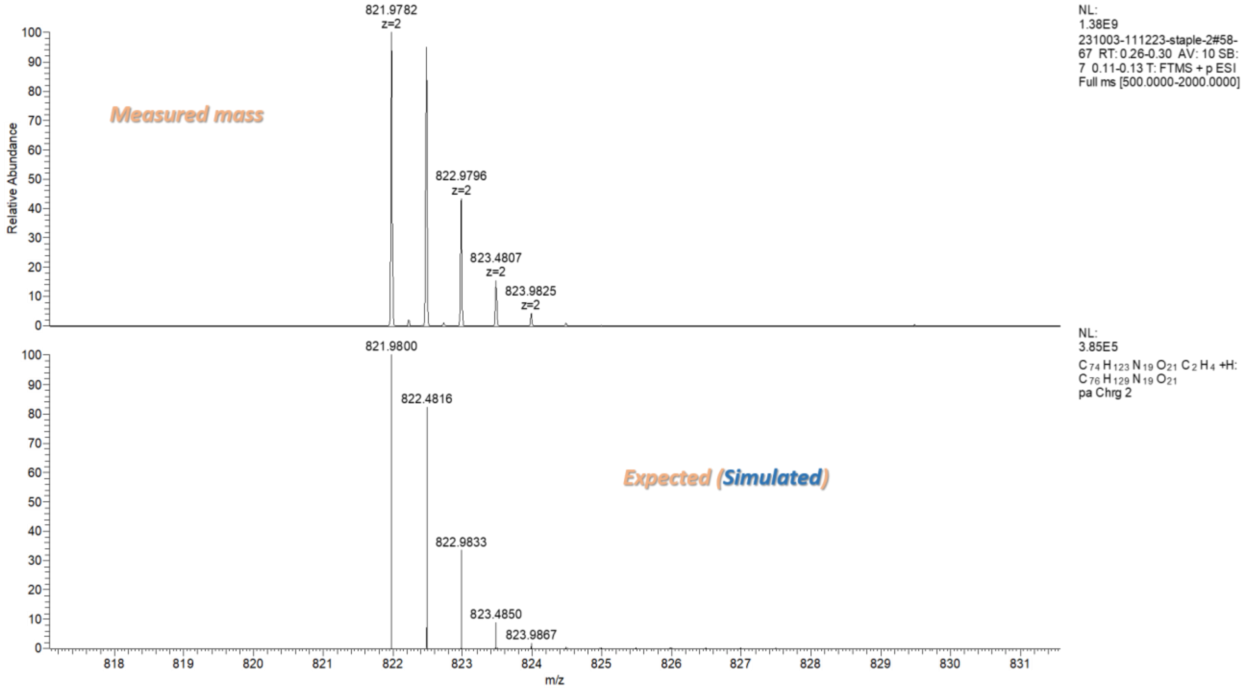

linear-f retention time: 11.932 min calculated $[M+H]^+$: 2195.05 calculated $[M+2H]^{2+}$: 1098.03 Observed $[M+H]^+$: 2195.0451 Observed $[M+2H]^{2+}$: 1098.0259

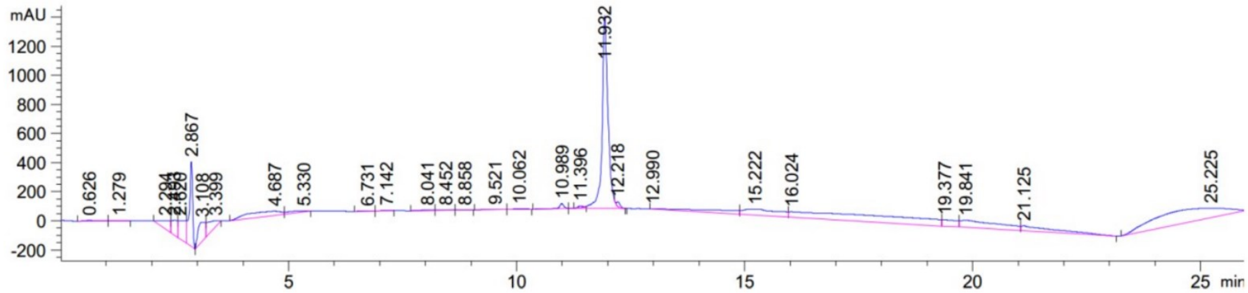

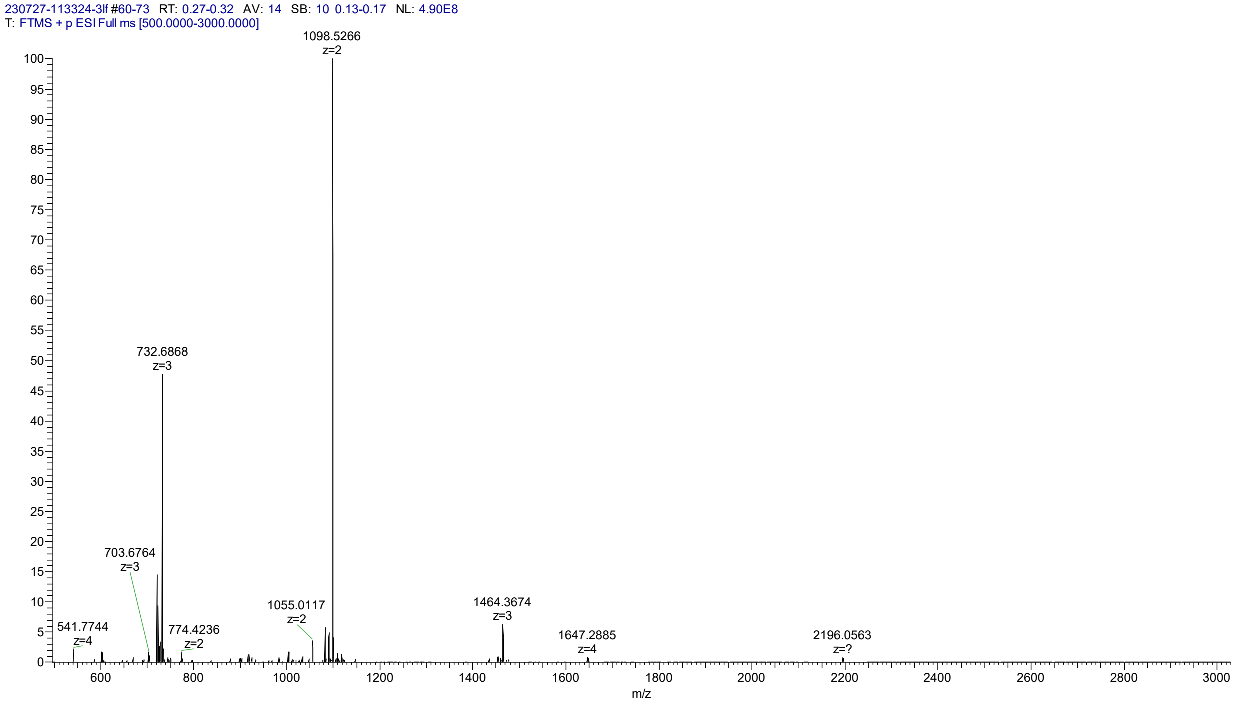

BAMM-f retention time: 13.173 min calculated $[M+2H]^{2+}$: 1281.04 calculated $[M+3H]^{3+}$: 854.36 Observed $[M+2H]^{2+}$: 1281.0407 Observed $[M+3H]^{3+}$: 854.3612

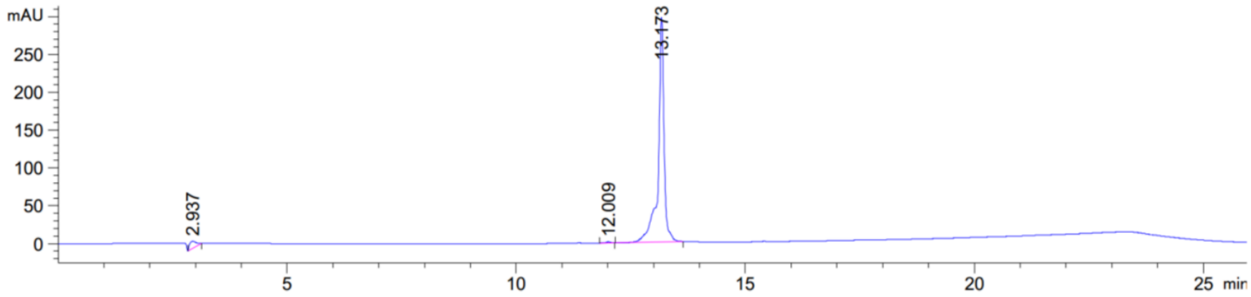

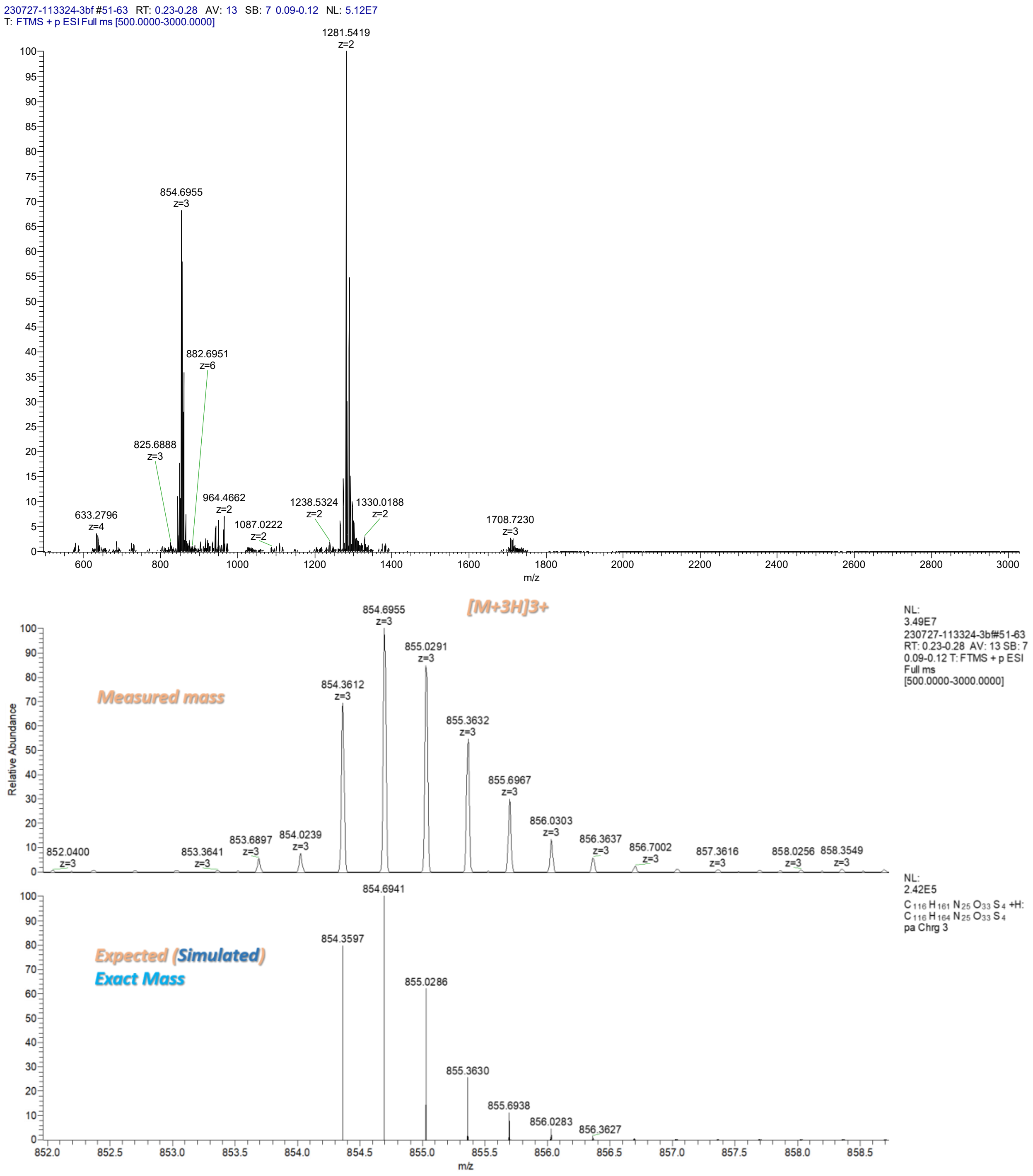
230727-113324-3bf #51-63 RT: 0.23-0.28 AV: 13 SB: 7 0.09-0.12 NL: 5.12E7
T: FTMS + p ESI Full ms [500.0000-3000.0000]
1281.5419 z=2
854.6955 z=3
882.6951 z=6
825.6888 z=3
964.4662 z=2
633.2796 z=4
1087.0222 z=2
1238.5324 z=2
1330.0188 z=2
1708.7230 z=3
m/z
[M+3H]3+
Measured mass
Relative Abundance
NL: 3.49E7
230727-113324-3bf#51-63
RT: 0.23-0.28 AV: 13 SB: 7
0.09-0.12 T: FTMS + p ESI
Full ms
[500.0000-3000.0000]
852.0400 z=3
853.3641 z=3
853.6897 z=3
854.0239 z=3
854.3612 z=3
854.6955 z=3
855.0291 z=3
855.3632 z=3
855.6967 z=3
856.0303 z=3
856.3637 z=3
856.7002 z=3
857.3616 z=3
858.0256 z=3
858.3549 z=3
NL: 2.42E5
C 116 H 161 N 25 O 33 S 4 +H:
C 116 H 164 N 25 O 33 S 4
pa Chrg 3
Expected (Simulated)
Exact Mass
854.3597
854.6941
855.0286
855.3630
855.6938
856.0283
856.3627
m/z

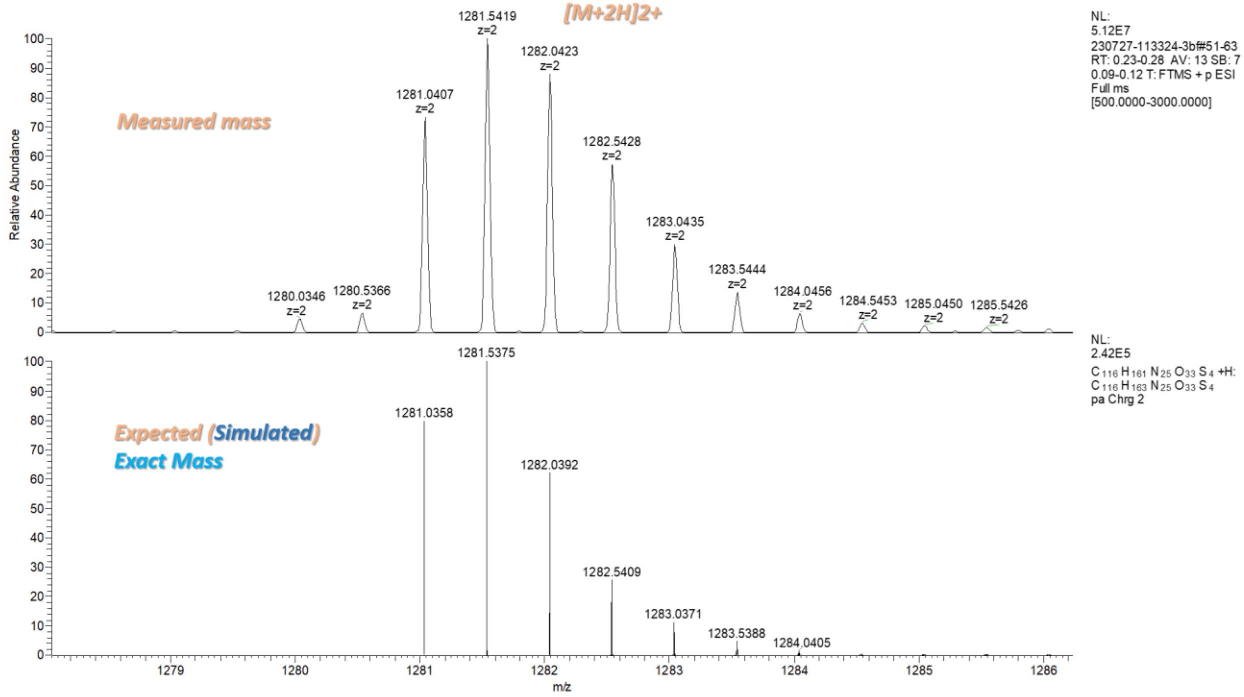

BSM-f retention time: 16.722 min calculated $[M+H]^+$: 2547.05 Observed $[M+H]^+$:2547.0611 Observed $[M+2H]^{2+}$: 1274.0270

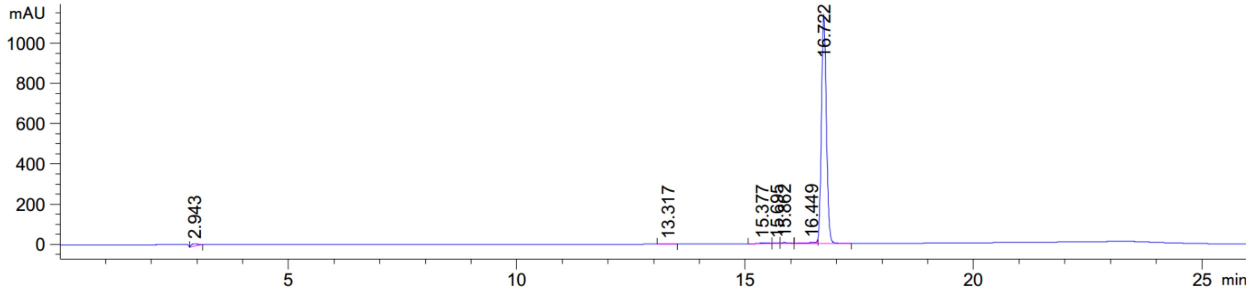

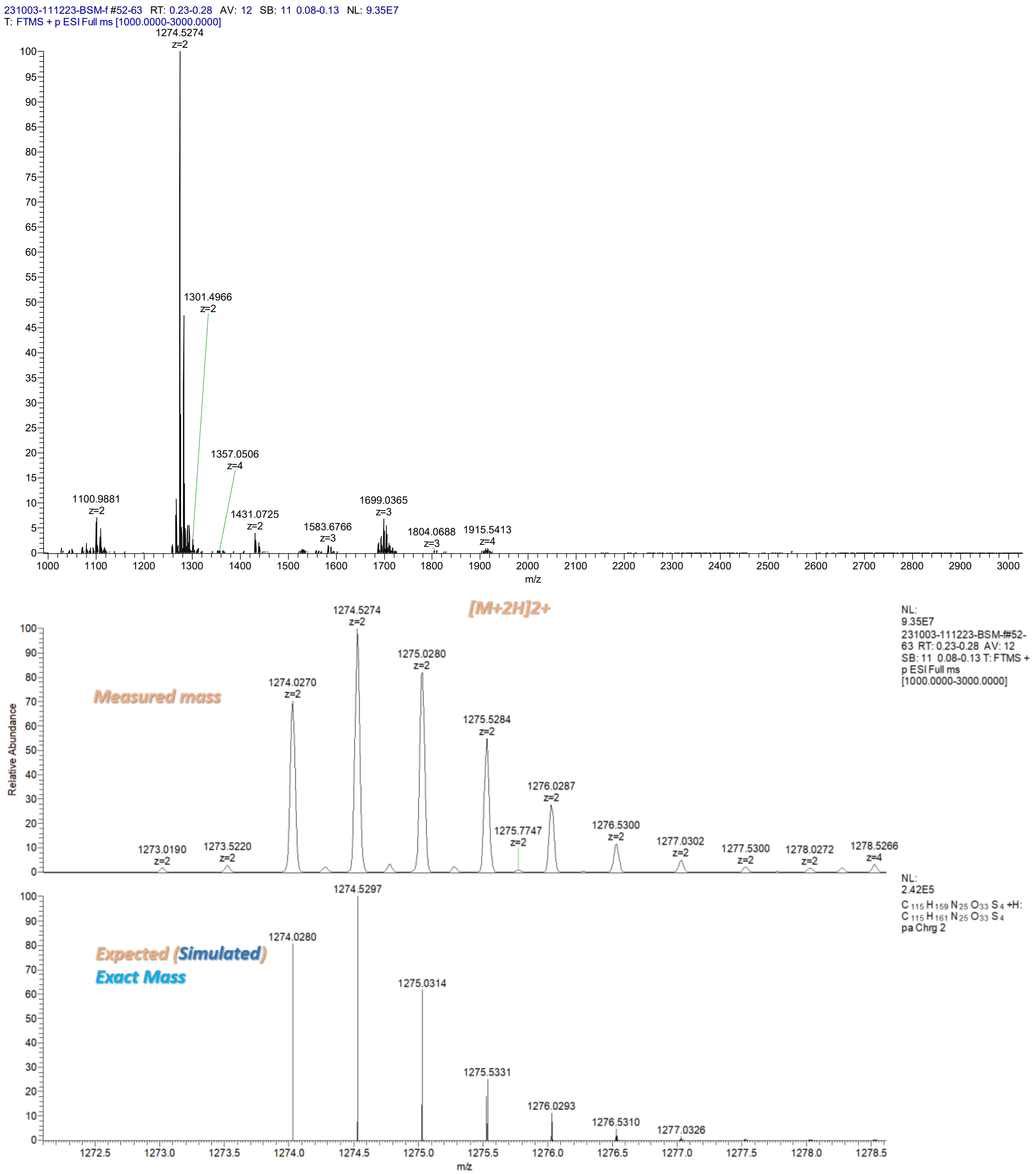

dual-f retention time: 18.477 min calculated $[M+H]^+$: 2913.07 Observed $[M+H]^+$: 2913.0829 Observed $[M+2H]^{2+}$: 1457.0368

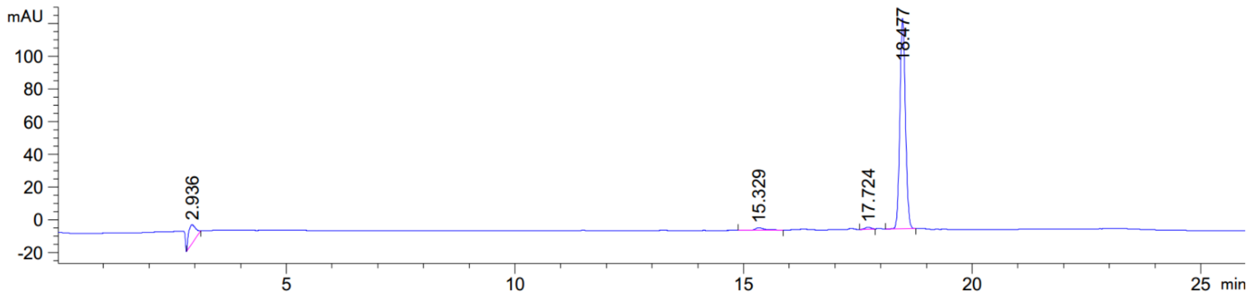
mAU
100
80
60
40
20
0
-20
2.936
15.329
17.724
18.477
5
10
15
20
25
min

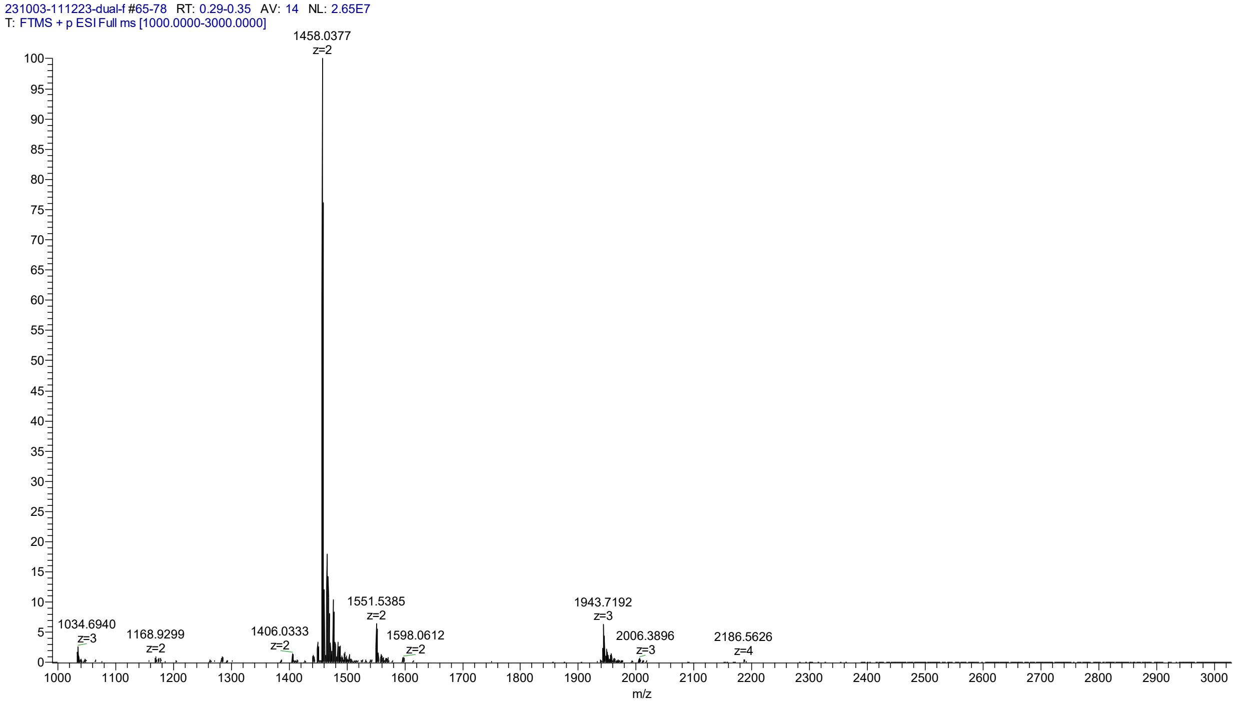
231003-111223-dual-f #65-78 RT: 0.29-0.35 AV: 14 NL: 2.65E7
T: FTMS + p ESI Full ms [1000.0000-3000.0000]
1458.0377
z=2
1034.6940
z=3
1168.9299
z=2
1406.0333
z=2
1551.5385
z=2
1598.0612
z=2
1943.7192
z=3
2006.3896
z=3
2186.5626
z=4
m/z

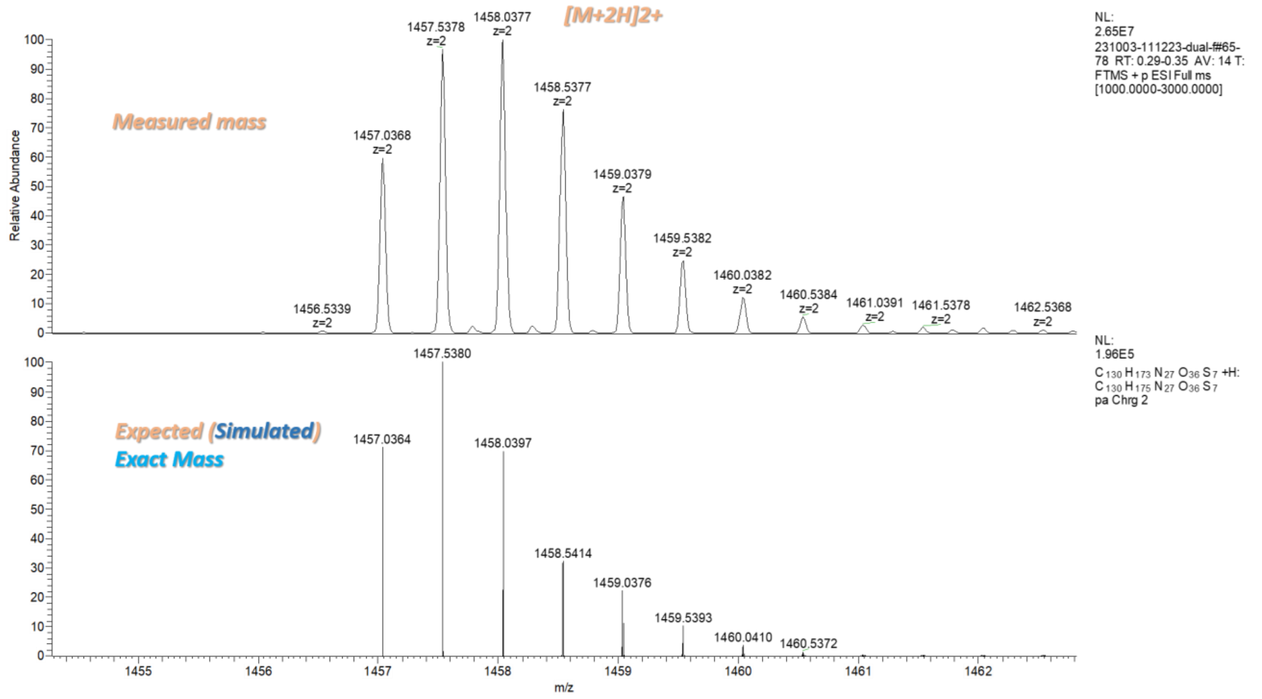
[M+2H]2+
Measured mass
Relative Abundance
1456.5339
z=2
1457.0368
z=2
1457.5378
z=2
1458.0377
z=2
1458.5377
z=2
1459.0379
z=2
1459.5382
z=2
1460.0382
z=2
1460.5384
z=2
1461.0391
z=2
1461.5378
z=2
1462.5368
z=2
NL:
2.65E7
231003-111223-dual-f#65-78 RT: 0.29-0.35 AV: 14 T: FTMS + p ESI Full ms [1000.0000-3000.0000]
Expected (Simulated)
Exact Mass
1457.0364
1457.5380
1458.0397
1458.5414
1459.0376
1459.5393
1460.0410
1460.5372
NL:
1.96E5
C 130 H 173 N 27 O 36 S 7 +H:
C 130 H 175 N 27 O 36 S 7
pa Chrg 2
m/z

staple-1-f retention time: 15.348 min calculated $[M+H]^+$: 2215.13 Observed $[M+H]^+$: 2215.1284 Observed $[M+2H]^{2+}$: 1108.0650

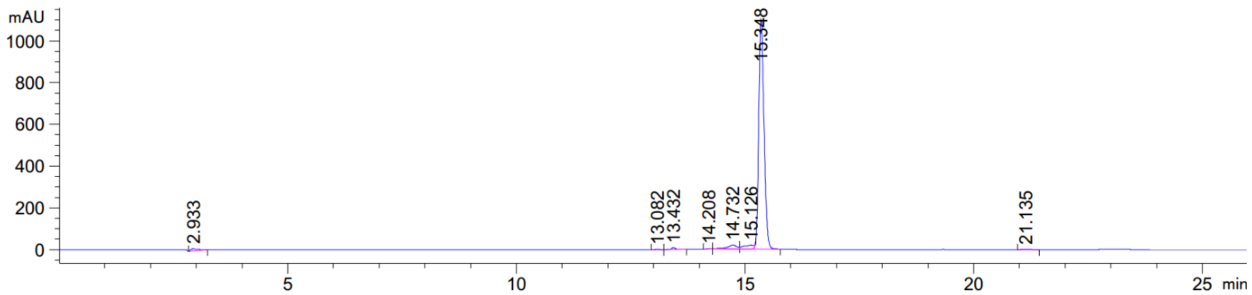

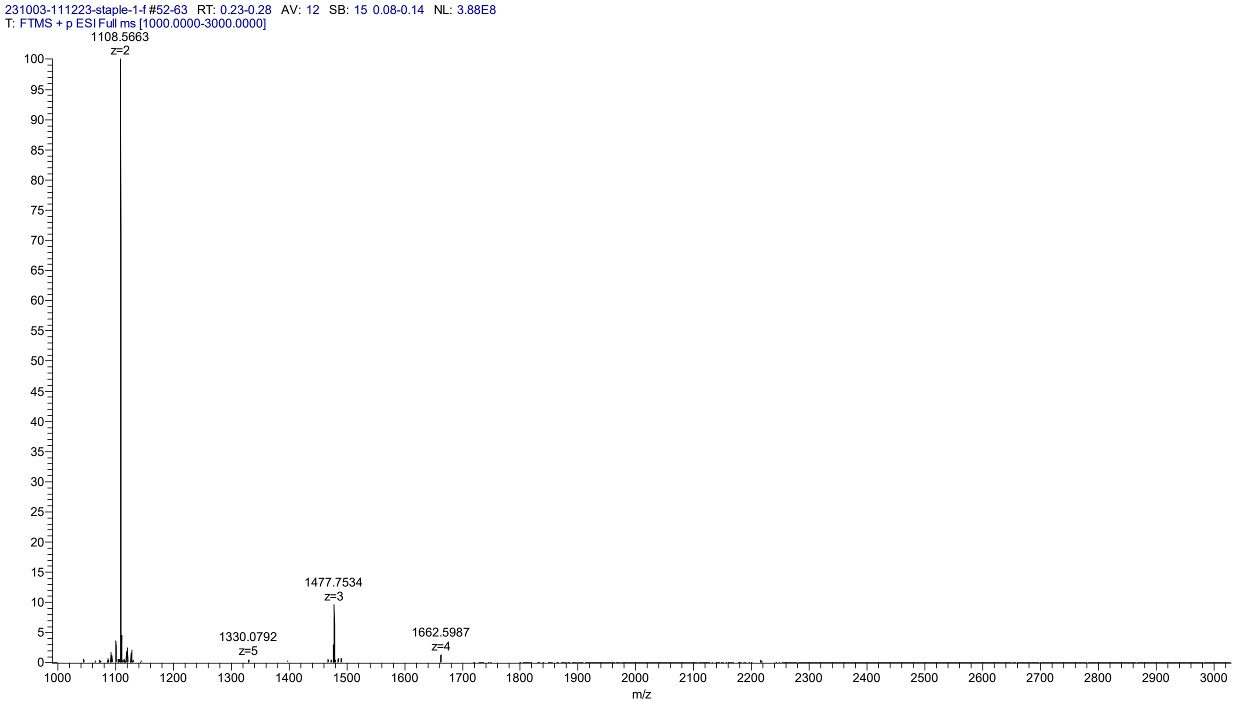

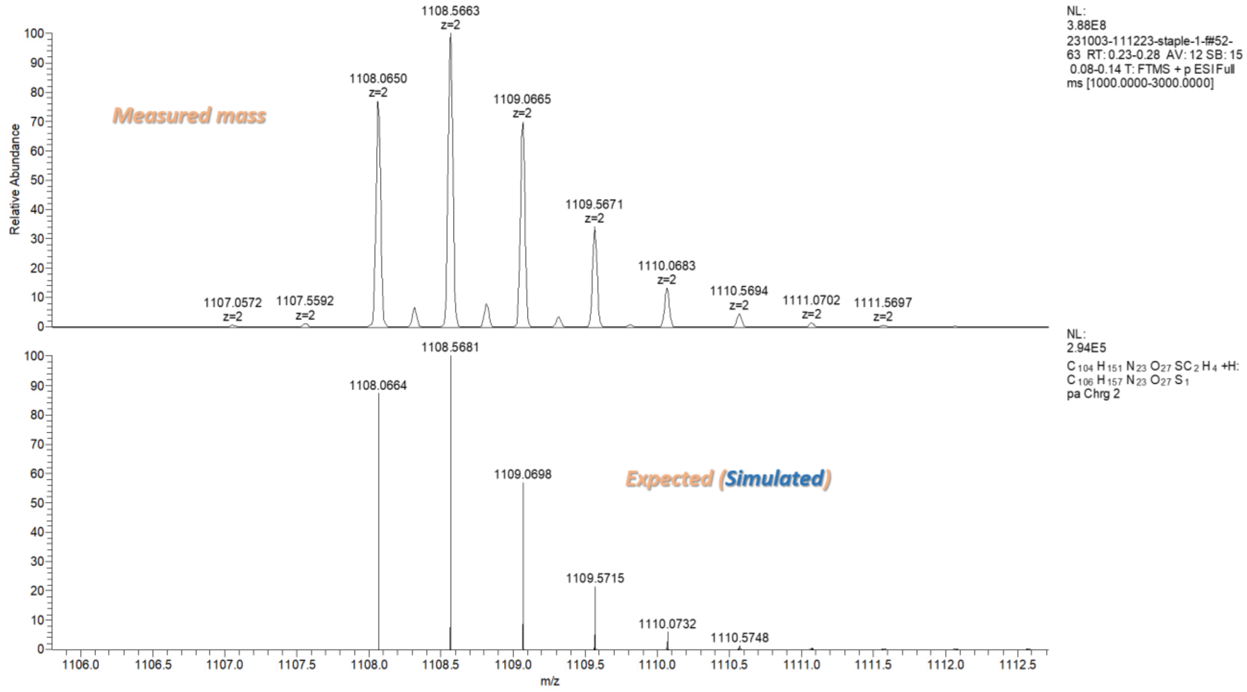

staple-2-f retention time: 14.130 min calculated $[M+H]^+$: 2203.09 Observed $[M+H]^+$: 2203.1036 Observed $[M+2H]^{2+}$: 1102.0471

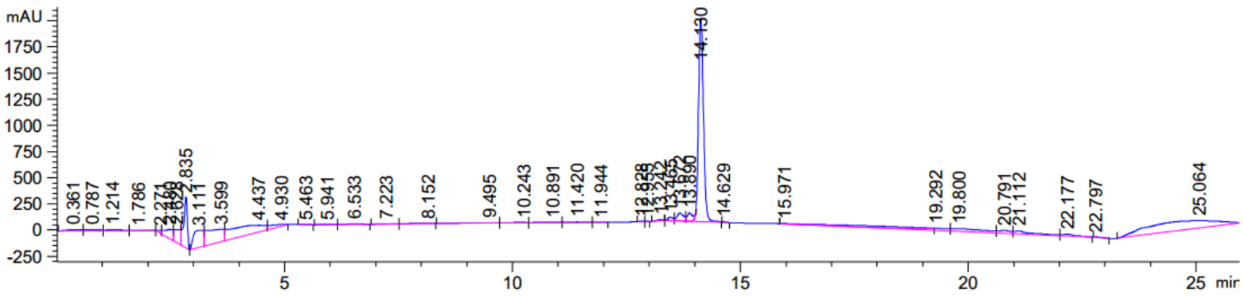

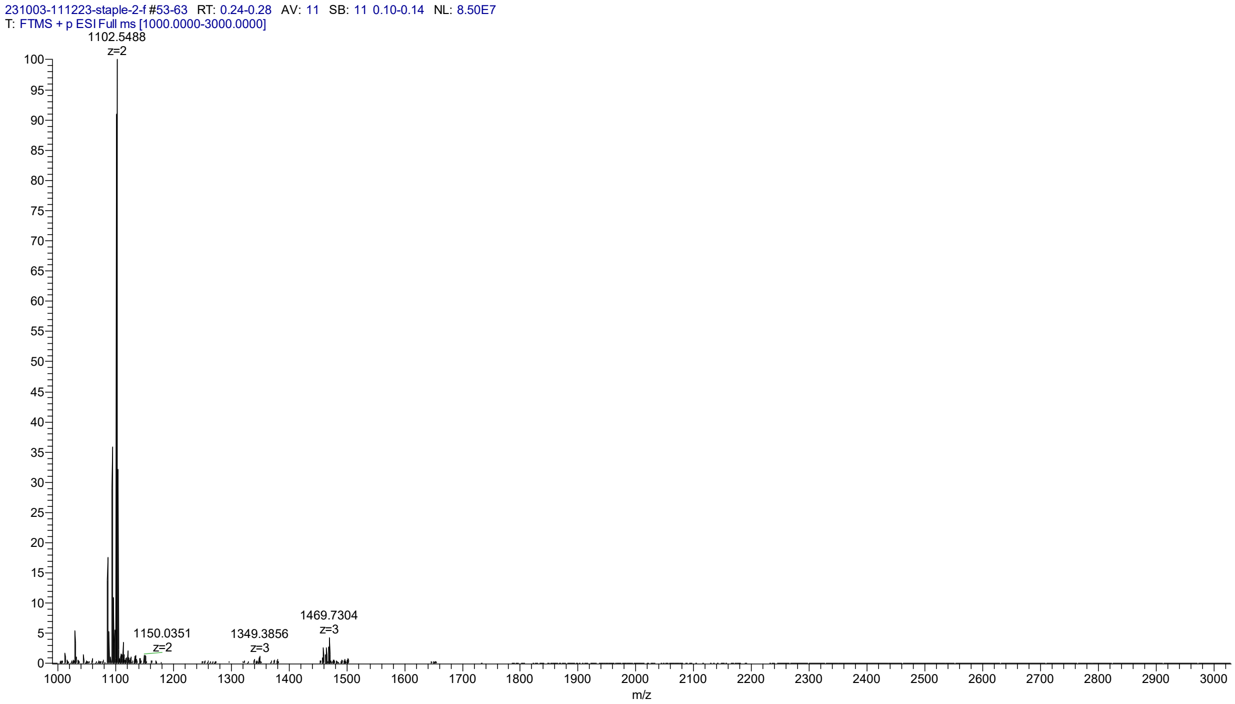
231003-111223-staple-2-f #53-63 RT: 0.24-0.28 AV: 11 SB: 11 0.10-0.14 NL: 8.50E7
T: FTMS + p ESI Full ms [1000.0000-3000.0000]
1102.5488
z=2
1150.0351
z=2
1349.3856
z=3
1469.7304
z=3
m/z

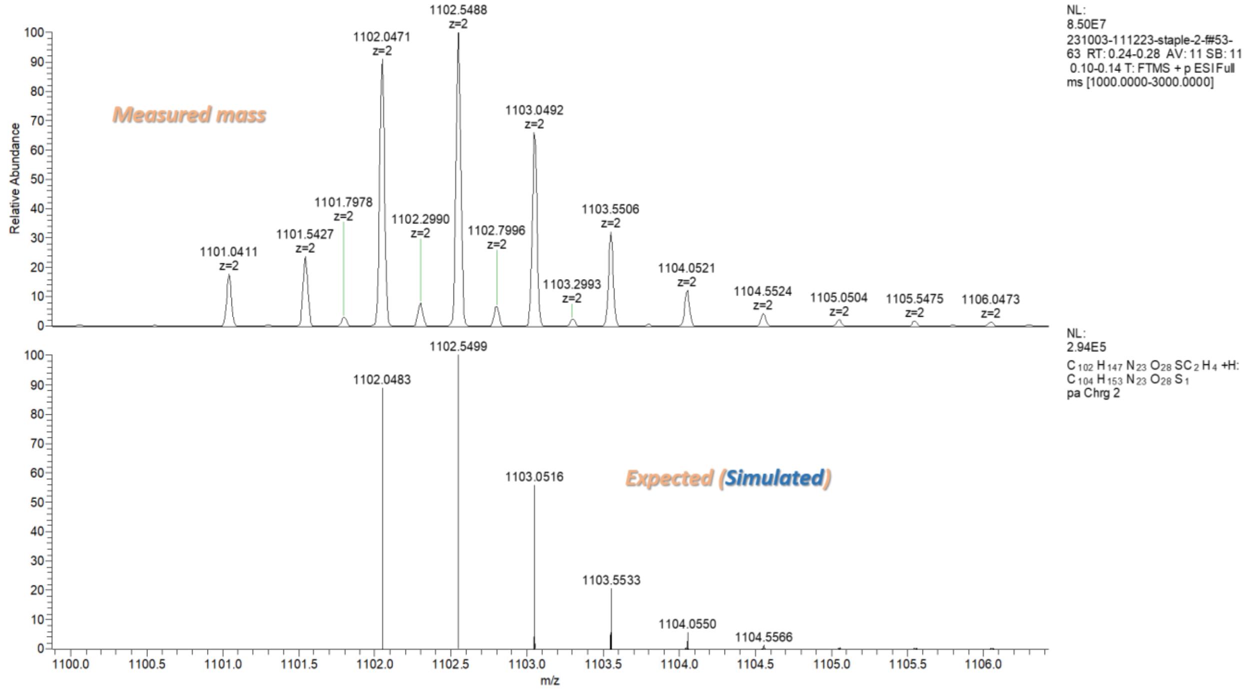
Measured mass
Relative Abundance
1101.0411
z=2
1101.5427
z=2
1101.7978
z=2
1102.0471
z=2
1102.2990
z=2
1102.5488
z=2
1102.7996
z=2
1103.0492
z=2
1103.2993
z=2
1103.5506
z=2
1104.0521
z=2
1104.5524
z=2
1105.0504
z=2
1105.5475
z=2
1106.0473
z=2
NL:
8.50E7
231003-111223-staple-2-f#53-63 RT: 0.24-0.28 AV: 11 SB: 11 0.10-0.14 T: FTMS + p ESI Full ms [1000.0000-3000.0000]
Expected (Simulated)
1102.0483
1102.5499
1103.0516
1103.5533
1104.0550
1104.5566
NL:
2.94E5
C 102 H 147 N 23 O 28 SC 2 H 4 +H:
C 104 H 153 N 23 O 28 S 1
pa Chrg 2
m/z

TMB-3C retention time: 8.429 min calculated $[M+H]^+$: 604.15 Observed $[M+H]^+$: 604.1449

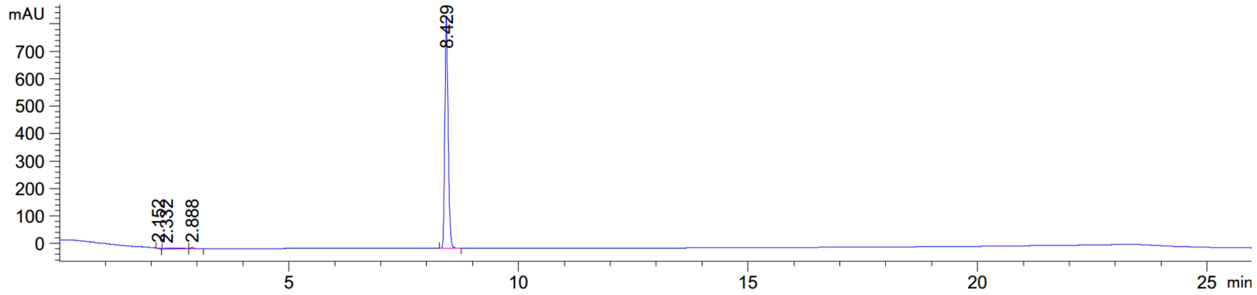

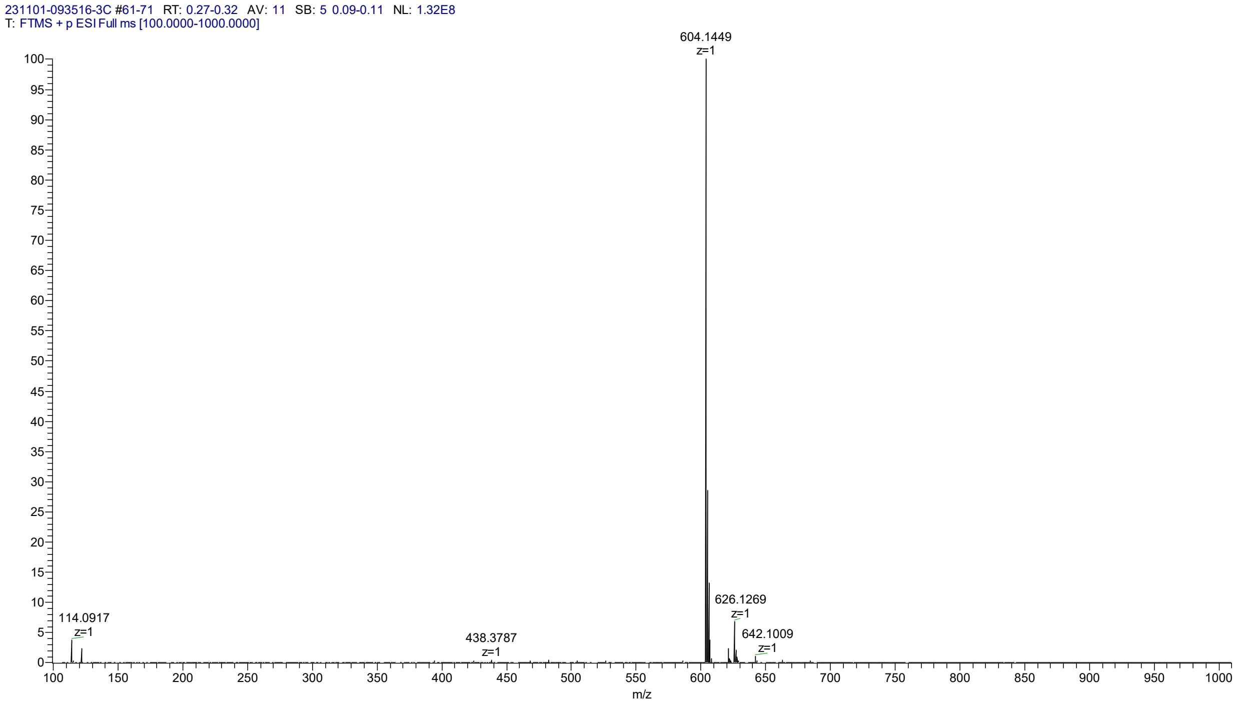

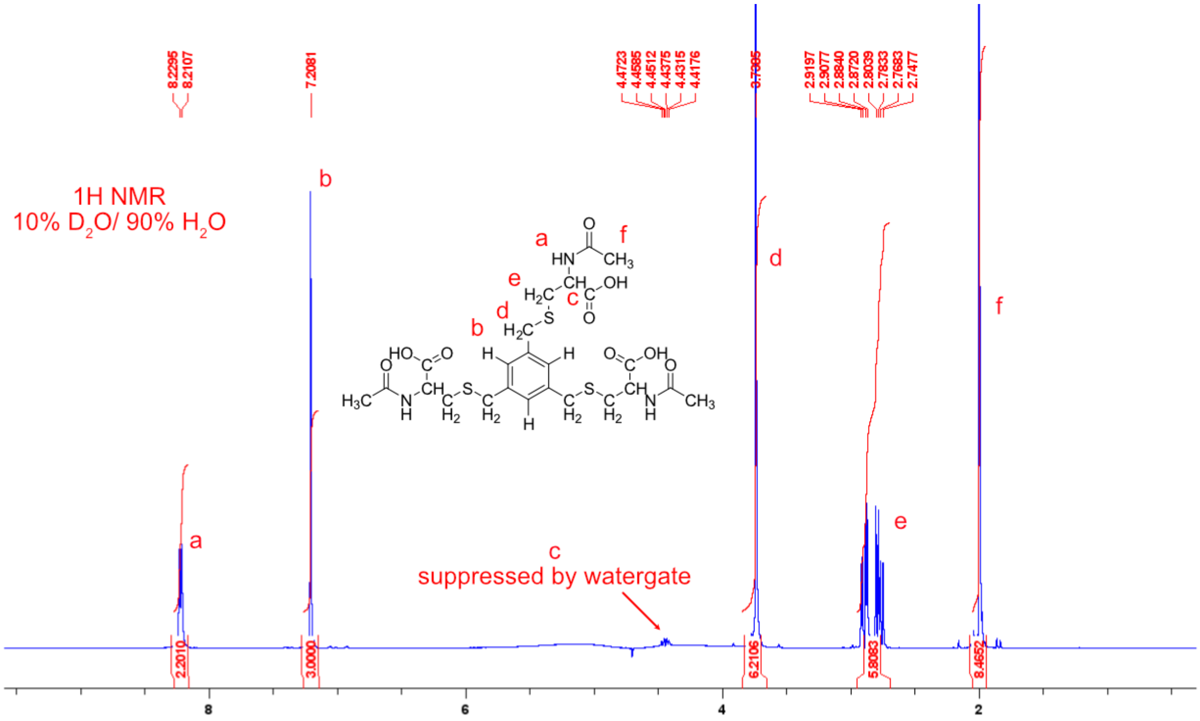
1H NMR
10% D2O/ 90% H2O
8.2295
8.2107
7.2081
4.4723
4.4585
4.4512
4.4375
4.4315
4.4176
3.7365
2.9197
2.9077
2.8840
2.8720
2.8039
2.7833
2.7683
2.7477
a
b
c
d
e
f
c
suppressed by watergate
2.2010
3.0000
6.2106
5.8083
8.4652
8
6
4
2

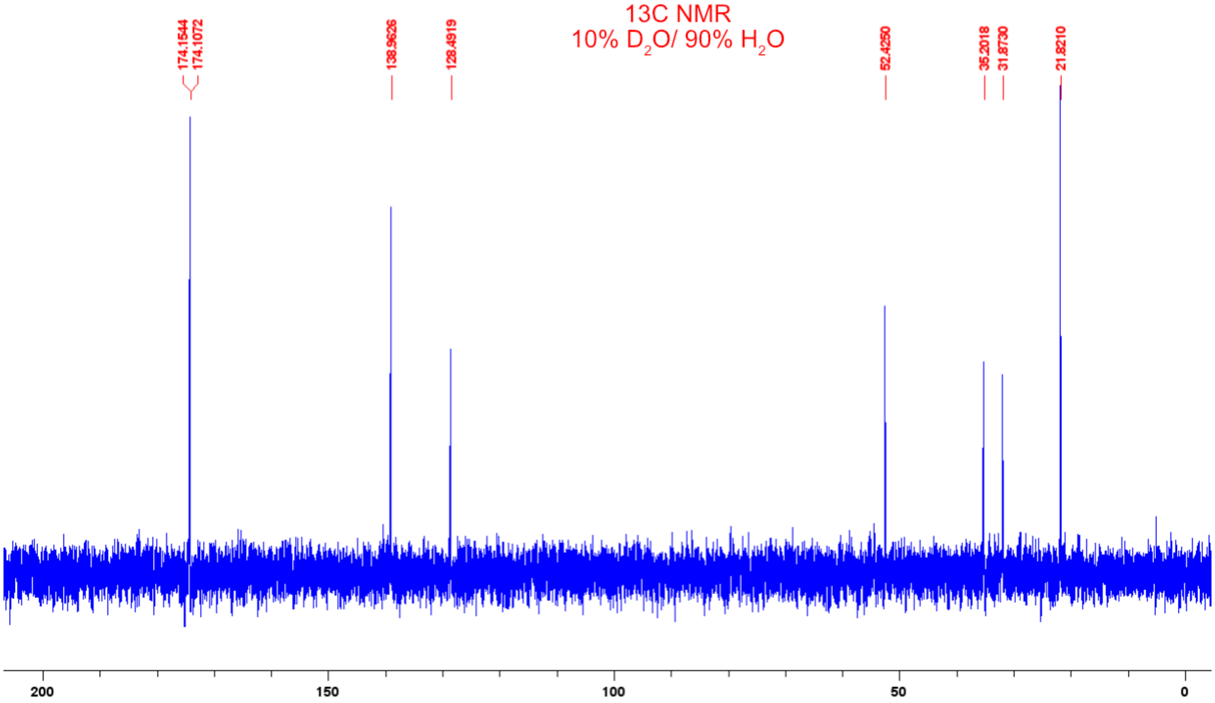
13C NMR
10% D2O/ 90% H2O
174.1544
174.1072
138.9626
128.4919
52.4250
35.2018
31.8730
21.8210
200
150
100
50
0

M. References

1 Kim, Y. W., Grossmann, T. N. & Verdine, G. L. Synthesis of all-hydrocarbon stapled α-helical peptides by ring-closing olefin metathesis. *Nat. Protoc.* **6**, 761-771, doi:10.1038/nprot.2011.324 (2011).

2 Jo, H. *et al.* Development of α-Helical Calpain Probes by Mimicking a Natural Protein-Protein Interaction. *J. Am. Chem. Soc.* **134**, 17704-17713, doi:10.1021/ja307599z (2012).

3 Li, D. & Elbert, D. L. The kinetics of the removal of the N-methyltrityl (Mtt) group during the synthesis of branched peptides. *J. Pept. Res.* **60**, 300-303, doi:10.1034/j.1399-3011.2002.21018.x (2002).

4 Luo, P. Z. & Baldwin, R. L. Mechanism of helix induction by trifluoroethanol: A framework for extrapolating the helix-forming properties of peptides from trifluoroethanol/water mixtures back to water. *Biochemistry* **36**, 8413-8421, doi:DOI 10.1021/bi9707133 (1997).

5 Rohl, C. A. & Baldwin, R. L. Deciphering rules of helix stability in peptides. *Energetics of Biological Macromolecules, Pt B* **295**, 1-26 (1998).

6 Hicks, M. R., Holberton, D. V., Kowalczyk, C. & Woolfson, D. N. Coiled-coil assembly by peptides with non-heptad sequence motifs. *Fold Des.* **2**, 149-158, doi:Doi 10.1016/S1359-0278(97)00021-7 (1997).

7 Demeler, B. UltraScan - A Comprehensive Data Analysis Software Package for Analytical Ultracentrifugation Experiments. *Analytical Ultracentrifugation: Techniques and Methods*, 210-230, doi:Book_Doi 10.1039/9781847552617 (2005).

8 Mi, T. X., Nguyen, D. & Burgess, K. Bicyclic Schellman Loop Mimics (BSMs): Rigid Synthetic C-Caps for Enforcing Peptide Helicity. *ACS Cent. Sci.* **9**, 300-306, doi:10.1021/acscentsci.2c01265 (2023).

9 Martins, J. M., Ramos, R. M., Pimenta, A. C. & Moreira, I. S. Solvent-accessible surface area: How well can be applied to hot-spot detection? *Proteins* **82**, 479-490, doi:DOI 10.1002/prot.24413 (2014).

10 Moreira, I. S. The Role of Water Occlusion for the Definition of a Protein Binding Hot-Spot. *Curr. Top. Med. Chem.* **15**, 2068-2079, doi:Doi 10.2174/1568026615666150519103733 (2015).

11 Wishart, D. S., Sykes, B. D. & Richards, F. M. The Chemical-Shift Index - a Fast and Simple Method for the Assignment of Protein Secondary Structure through NMR-Spectroscopy. *Biochemistry* **31**, 1647-1651, doi:DOI 10.1021/bi00121a010 (1992).

12 Zerbe, O. & Bader, R. *Peptide NMR*, <https://www.chem.uzh.ch/zerbe/PeptidNMR.pdf>

13 Olanders, G., Alogheli, H., Brandt, P. & Karlén, A. Conformational analysis of macrocycles: comparing general and specialized methods. *J. Comput. Aid. Mol. Des.* **34**, 231-252, doi:10.1007/s10822-020-00277-2 (2020).

14 Pace, J. R. *et al.* Stapled β-Hairpins Featuring 4-Mercaptoproline. *J. Am. Chem. Soc.* **143**, 15039-15044, doi:10.1021/jacs.1c04378 (2021).

15 *Fluorescence Polarization Technical Resource Guide*. <https://research.fredhutch.org/content/dam/stripe/hahn/methods/biochem/beacon_fluorescence_guide.pdf> (2006).

16 Salangros, A., Tamarelle, D. & Onado, V. What Methods to Estimate Kd and Ki in Fluorescence Polarization? Methodology and Examples. <https://ncs-conference.org/download/slide/20120925_Tuesday/1100_1230_bioassays/4_Salangros_Tamarelle.pdf>

17 Yammine, A., Gao, J. L. & Kwan, A. H. Tryptophan Fluorescence Quenching Assays for Measuring Protein-ligand Binding Affinities: Principles and a Practical Guide. *Bio-Protocol* **9**, doi:10.21769/BioProtoc.3253 (2019).
